# Clinical investigation on nebulized human umbilical cord MSC-derived extracellular vesicles for pulmonary fibrosis treatment

**DOI:** 10.1038/s41392-025-02262-3

**Published:** 2025-06-04

**Authors:** Meng Li, Huaping Huang, Xiaofei Wei, Huajuan Li, Jun Li, Bingchen Xie, Yuze Yang, Xingyue Fang, Lei Wang, Xiaona Zhang, Heyu Wang, Mengdi Li, Yuting Lin, Dezhi Wang, Yinyin Wang, Tongbiao Zhao, Jianqiu Sheng, Xinbao Hao, Muyang Yan, Lu Xu, Zhijie Chang

**Affiliations:** 1 State Key Laboratory of Membrane Biology, School of Medicine, Institute of Precision Medicine, Tsinghua University, Beijing, 100084 China; 2 Department of Respiratory Diseases, The First Affiliated Hospital of Hainan Medical University, Haikou, Hainan China; 3 Beijing cord blood bank, Beijing, China; 4 Jinfeng Laboratory, High-tech Zone, Chongqing, China; 5 School of Biomedical Engineering, Capital Medical University, Beijing, China; 6 Department of Hematology, The First Affiliated Hospital, Hainan Medical University, Haikou, Hainan China; 7 Medical School of Chinese PLA, Chinese PLA General Hospital, Beijing, China; 8 Beijing Institute for Stem Cell and Regenerative Medicine, Beijing, China; 9 First Medical Center, Chinese PLA General Hospital, Beijing, China; 10 Precision medicine institute, Changgeng Hospital, Tsinghua University, Beijing, China

**Keywords:** Mesenchymal stem cells, Respiratory tract diseases

## Abstract

Mesenchymal stromal cell-derived extracellular vesicles (MSC-EVs) are recognized as a promising strategy for cell-free therapy, however, their therapeutic role in pulmonary fibrosis remains unrevealed. Here, we report the safety and efficacy of MSC-EVs from human umbilical cord (hUCMSC-EVs) evaluated in mouse models and pulmonary fibrosis patients. We established a rigorous system to produce high-quality of hUCMSC-EVs, characterized by miRNA, protein, and metabolite profiles. When administered via nebulization, hUCMSC-EVs predominantly accumulated in murine lungs and ameliorated bleomycin-induced pulmonary fibrosis, with increased survival rate (from 20% to 80%), restored lung volume, and attenuated injury severity accompanied by elevated oxyhemoglobin saturation and improved pulmonary function evaluations. We performed a phase l clinical trial involving twenty-four patients in a randomized, single-blind, and placebo-controlled study to treat pulmonary fibrosis (MR-46-22-004531, ChiCTR2300075466). All participants tolerated the nebulized hUCMSC-EVs well, with no serious adverse events. Patients receiving the combined therapy of nebulized hUCMSC-EVs and routine treatment demonstrated significant improvements in both lung function indices (forced vital capacity and maximal voluntary ventilation) and respiratory health status (as measured by the Saint George’s Respiratory Questionnaire and Leicester Cough Questionnaire. Overall, patients upon the additional therapy with nebulized hUCMSC-EVs gained significant benefits compared with those accepted only routine treatment. Remarkably, two patients with advanced post-inflammatory pulmonary fibrosis exhibited clinically significant regression on serial CT scans after hUCMSC-EVs therapy. These findings suggest that nebulized hUCMSC-EVs could be used as a promising therapeutic strategy for treating pulmonary fibrosis diseases.

## Introduction

Pulmonary fibrosis is a chronic and progressive interstitial lung disease characterized by the abnormal accumulation of scar tissues in the pulmonary parenchyma.^1^ This disease is featured with fibrotic transformation, which induces thickening and rigidity in lung tissues, ultimately impeding their physiological functionality.^2^ As the disease progresses, the capacity for efficient oxygen exchange diminishes gradually, precipitating symptoms like dyspnea, persistent cough, and fatigue.^3^ The process of pulmonary fibrosis has been recognized as a cascade of inflammation involving the activation of immune cells such as macrophages^4^ and T-cells.^5^ The activated macrophages and T cells secrete a bunch of cytokines and chemokines, which aggregate the inflammation by recruiting more immune cells. At the same time, these cytokines boost the resistant fibroblast or activate the quiescent fibroblasts to secrete extracellular matrix such as fibronectin, collagens and MMPs. The accumulation of extracellular matrix reconstitutes the normal lung organs and destroys the alveolar to block the exchanges of oxygen.^6,7^ Currently, pulmonary fibrosis is attributed to be a multifaceted disease, caused by diverse factors such as environmental exposures, autoimmune disorders, genetic predispositions, certain pharmacological agents, and viral infections.^8^ In particular, viral infections, including but not limited to influenza viruses and coronaviruses, have been implicated as major pathogenic factors contributing to the development of pulmonary fibrosis. Viral infections may directly damage lung tissue, trigger immune responses, or activate fibroblasts, thereby boosting lung inflammation and fibrosis.^9^ These challenges may last for a long time and cause damage, which further exacerbates the inflammation for the patients. Therefore, the chronic inflammation gradually becomes severe and worsens the condition of the patients, which makes these diseases incurable.

Despite significant improvements in comprehending the pathogenesis of pulmonary fibrosis, the quest for efficacious treatments for this incurable disease persists.^10^ Current therapeutic modalities predominantly revolve around symptom management to decelerate disease progression, rather than to directly ablate the fundamental causes that orchestrate the fibrosis.^11^ For instance, dexamethasone, pirfenidone,^12^ and nintedanib^13^ are widely used in the early stages of pulmonary fibrosis, but the disease often continues to progress. These treatments repress the chronic inflammation by regulating the immune responses, including the activation of macrophages, T cells, and fibroblasts. Unfortunately, all these treatments could not cure the disease but only slow down the progress of fibrosis. Therefore, more innovative treatment paradigms are needed to treat the disease and simultaneously to foster the regeneration of lung tissues.

Human umbilical cord mesenchymal stem cells (UCMSCs) have been widely reported to the therapy of different chronic diseases, including pulmonary fibrosis.^14^ Many studies demonstrated that UCMSCs were easy to prepare and had a wide range of safety in the therapy window. Recently, reports showed that UCMSCs had a preference in the treatment of pulmonary disease and acute respiratory distress syndrome.^15^ Studies also indicated that the major role of UCMSCs functions to regulate the immune system in a variety of inflammatory diseases.^16^ Our previous study confirmed that USMSCs retained superior anti-fibrotic abilities in treating lung damage.^17^ However, more clinical practices realized some shortages of MSCs, including their potential differentiation in vivo, immune responses in long-term treatment, and difficulties in storage and delivery.^18^ Therefore, human umbilical cord mesenchymal stem cell-derived extracellular vesicles (hUCMSC-EVs), small membrane-bound particles, have been developed for the therapy of diseases, as many studies revealed the MSCs function via their EVs in vivo.^19,20^ These vesicles, characterized by the specific molecules such as CD9, CD63, and CD81, exert potent therapeutic effects through their cargo of bioactive molecules, including proteins, nucleic acids (in particular, mRNA, tRNA, rRNA, snoRNA, snRNA and other RNAs), and metabolites including lipids.^21^ hUCMSC-EVs have been shown to possess anti-inflammatory, anti-fibrotic,^22^ and regenerative properties due to their rich compotents.^23^ These effects appear similar to the functions of UCMSCs, which regulate the immune responses by macrophages and T cells during the process of chronic diseases via different components, including their secreted cytokines.^24^ Additionally, the accessibility of UCMSC-EVs for preparation, storage, and delivery to the bedside offers distinct advantages over cell-based therapies, along with their ability to reduce the associated risk of pulmonary embolism.^25^ In recent years, hUCMSC-EVs have been used as a promising strategy for the treatment of pulmonary fibrosis in mouse models.^26–28^

Although MSC-EV injection is preferred for the treatment of other diseases, including liver diseases^29^ and cardiovascular diseases,^30^ recent advancements in drug delivery techniques have enabled the transition from traditional intravenous injection to nebulized inhalation as a specifically targeted and efficient route of administration.^31–34^ Nebulized delivery allows for the direct deposition of therapeutic agents in the lung, maximizing their local concentration and minimizing systemic adverse effects. This approach demonstrates a potential for improving therapeutic outcomes and enhancing patient compliance in the management of pulmonary fibrosis. In this study, we provided a procedure for manufacturing hUCMSC-EVs with quality control, and performed a phase l clinical trial to treat pulmonary fibrosis (MR-46-22-004531, ChiCTR2300075466), with a series of preclinical studies in mice. Our results demonstrate that nebulized hUCMSC-EVs are effective in ameliorating the symptoms of pulmonary fibrosis with no adverse side effects in patients, highlighting the potential of nebulized MSC-EVs as a novel therapeutic approach.

## Result

### The manufacturing and characterization of hUCMSC-EVs

**Fig. 1:**
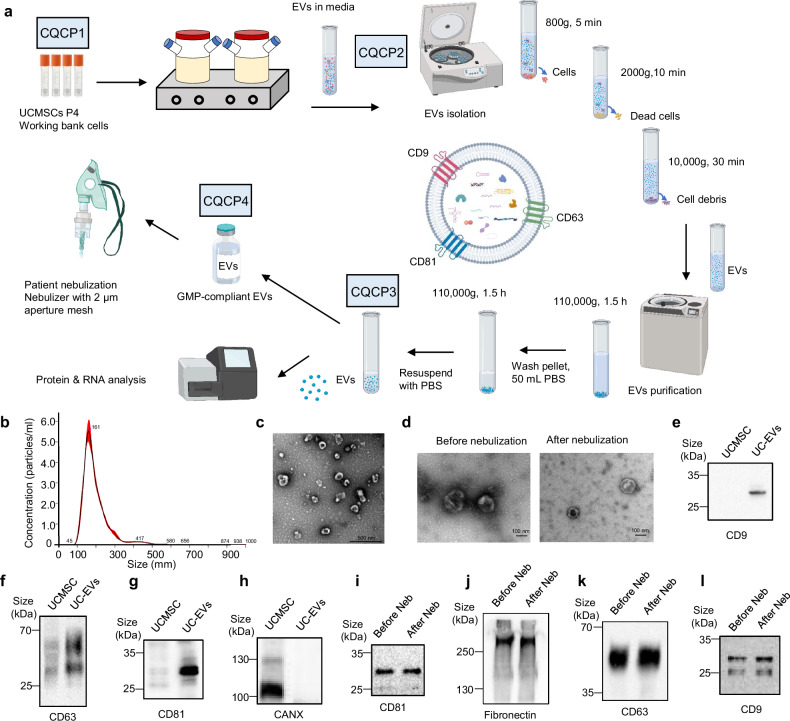
The manufacture and characterization of hUCMSC-EVs. **a** hUCMSC-EVs manufacturing process and critical quality control points (CQCP). UCMSCs were prepared from a working bank with culture for 4 passages (P4). CQCPs were performed at the different stages of the cell expansion and EV isolation. The final EVs were stored for the patient application with nebulizer. The major manufacturing parameters were labeled. This figure was created using MedPeer (www.medpeer.cn). **b** The concentration and size distribution of hUCMSC-EVs. hUCMSC-EVs were determined by Nanoparticle Tracking Analysis (NTA). The specific EV sizes were labeled. The distribution curve (black) was fit from the examined value (red). **c** Representative electron microscopic (EM) photograph of hUCMSC-EVs, scale bar=500 nm. hUCMSC-EVs were prepared for the image by EM. **d** Representative electron microscopic photographs of hUCMSC-EVs before and after nebulization. hUCMSC-EVs were collected from condensation after nebulization. scale bar = 100 nm. Levels of EV markers. Western blots were performed for the protein levels of EV marker including CD9 (**e**), CD63 (**f**), CD81 (**g**), and CANX (**h**). The EV marker alteration after nebulization. Western blots showed the protein markers in the hUCMSC-EVs before and after nebulization (Neb). CD81 (**i**), fibronectin (**j**), CD63 (**k**) and CD9 (**l**) were examined

To ensure reliable, safe, and consistently stable extracellular vesicles (EVs), we established a working cell bank and developed an industrialized process (Fig. 1a). A comprehensive system was implemented to strictly maintain good manufacturing practice (GMP) criteria at all stages as described in the Materials and Methods. Nanoparticle Tracking Analysis (NTA) results indicated that hUCMSC-EVs exhibited a size distribution ranging from 50 to 400 nm in diameter (Fig. 1b). The morphology of hUCMSC-EVs was observed using transmission electron microscopy examination (Fig. 1c). We examined the morphology of EVs before and after nebulization using electron microscopy and observed no morphological changes (Fig. 1d). Furthermore, hUCMSC-EVs demonstrated an enrichment in specific markers, including CD9, CD81, and CD63, but not in CANX, a marker expressed in UCMSCs (Fig. 1e–h). A Western blot analysis showed no difference in their total amount of EV marker proteins before and after nebulization, indicating that the nebulizer has no impact on the morphology or quality of EVs (Fig. 1i–l). These observations validated the successful manufacture and production of hUCMSC-EVs.

### Quality control at critical processing points

To ensure the quality of hUCMSC-EVs, critical quality control points (CQCPs) were implemented for the working bank cells, cell culture supernatant, purified hUCMSC-EVs, and nebulized hUCMSC-EVs, respectively (Fig. 1a, Table 1). Firstly, CQCP 1 was established during the generation of the working cell bank by confirming the status of hUCMSCs without deletions, translocations, mutations, and cell short tandem repeat (STR) alterations (Supplementary Fig. 1a). Secondly, CQCP 2 was performed for the cell culture supernatant to avoid any potential contamination of mycoplasma and exogenous virus (data not shown). Subsequently, CQCP 3 was performed on purified hUCMSC-EVs to test the mycoplasma, exogenous virus, sterility, and antibiotic residue (data not shown). Simultaneously, an NTA analysis was performed to confirm the particles between 50 and 400 nm in a constitution of over 90% total EVs (Supplementary Fig. 1b). Additionally, to assess the biological activity of hUCMSC-EVs, the NF-κB signaling pathway was examined in co-cultured cells (Supplementary Fig. 1c), and their immunomodulatory effects were confirmed by the inhibition of Th1/2/17 responses and promotion of Treg cells (Supplementary Fig. 1d). Finally, CQCP 4 was applied to the nebulized hUCMSC-EVs by rechecking mycoplasma, exogenous virus, and sterility to ensure their non-contamination status (data not shown). Furthermore, the endotoxin was examined to allow its level with a threshold set below 0.25 EU/ml. An NTA analysis was performed to warrant that the working concentration of hUCMSC-EVs remained within ±20% of the labeled level (data not shown). Overall, all of the CQCPs were rigorously operated to guarantee the quality of hUCMSC-EVs with expected concentrations and biological activities.

**Table 1:** Quality control at critical processing points

Parameter	Release criteria	Method
CQCP1: working bank cell		
Cell karyotyping	Normal with no deletions, translocations and mutations	Karyotyping system
Cell STR profiling	No cross-contamination	Short Tandem Repeats alignment
CQCP2: cell culture supernatant		
Mycoplasma	Negative	qPCR
Exogenous virus	Negative	According to ChP
CQCP3: purified EVS		
Mycoplasma	Negative	qPCR
Exogenous virus	Negative	According to ChP
Sterility	Negative	Microbial culture system
Particle analysis	50-400 nm particles > 90%	NTA
Identity marker	CD9 + , CD63 + , CD81 + , CANX −	WB
Antibiotic residue	Negative	ELISA
Biological activity	Inhibition of NF-κB signaling pathway in co-cultured cells	Luciferase
Immune response	Inhibit Th1/2/17 and promote Treg	FACS
Protein concentration	-	BCA
CQCP4: EVs nebulization drug		
Mycoplasma	Negative	qPCR
Exogenous virus	Negative	According to ChP
Sterility	Negative	Microbial culture system
Endotoxin	< 0.25 EU/ml	Limulus assay
Particle analysis	Labeled amount ± 20%	NTA

### RNA contents of hUCMSC-EVs

**Fig. 2:**
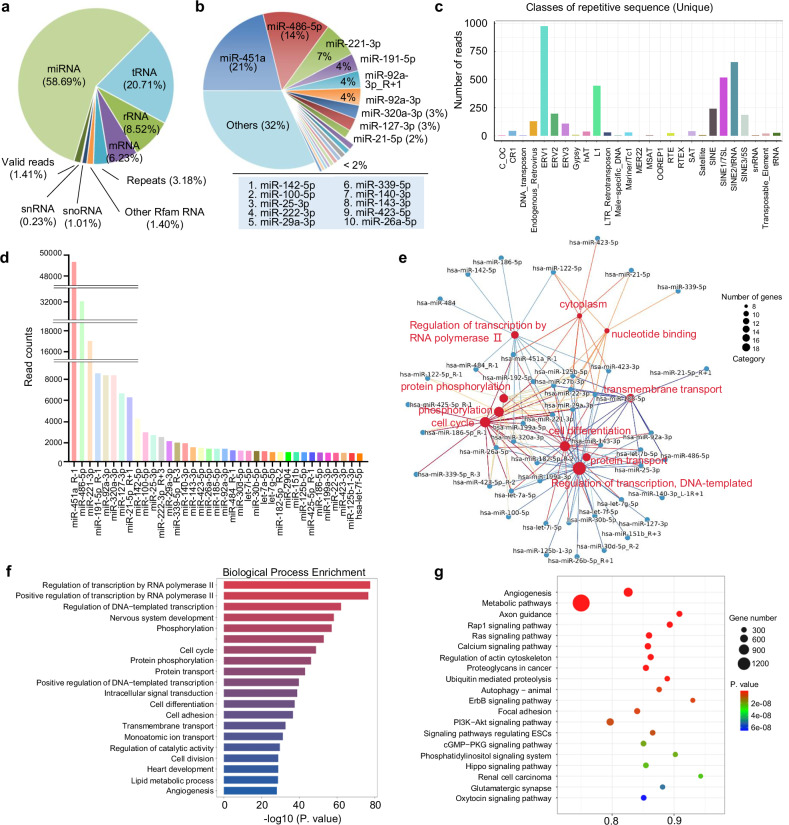
RNA contents of hUCMSC-EVs. **a** A composition analysis for the common non-coding and coding RNAs. **b** The pie chart shows the top 20 miRNAs, which collectively account for 68% of the total miRNAs present in hUCMSC-EVs. **c** An analysis of prototypic sequences representing repetitive sequence. **d** Total reads of the top 37 miRNAs are shown. All miRNAs were normalized to positive and negative controls. **e** An enrichment map of biological processes targeted by the top 37 miRNAs in hUCMSC-EVs. Nodes represent the individual genes of significantly enriched biological processes and the miRNAs which target them, connected by the edges. Genes related to the biological processes are shown as small red circles, miRNA genes are shown in blue circles. miRNAs targeting similar gene clusters are located in close proximity to one another. Each color line represents a biological process. **f** Enriched biological processes and **g** enriched KEGG pathways targeted by the miRNAs

As RNAs in particular miRNAs have been attributed as main functional molecules in EVs,^35^ we performed a comprehensive small RNA deep sequencing to profile the RNA abundance for hUCMSC-EVs. The results showed that various RNAs were present in hUCMSC-EVs, with miRNAs as the predominant type (58.69%), followed by tRNAs (20.72%), rRNAs (8.53%), repetitive elements (3.18%), snoRNAs (1.01%), snRNAs (0.23%), and a small portion of coding mRNAs (6.23%) (Fig. 2a, Supplementary Data 1). Over 1400 unique miRNAs were identified, with the top 20 accounting for 68% of total miRNA reads (Fig. 2b, Supplementary Data 2), while the remaining miRNAs contributed only 32% of the reads. Notably, 3.18% of the RNAs corresponded to repetitive elements such as transposons, retrotransposons, and endogenous retroviruses with unclear functions (Fig. 2c). These findings highlight the RNA diversity in hUCMSC-EVs. Focusing on miRNAs, we ranked the top 37 by read counts (Fig. 2d). Gene Ontology analysis indicated that these miRNAs mainly regulate biological processes such as phosphorylation, cell differentiation, and transmembrane transport (Fig. 2e). Further analysis revealed that their target genes are involved in transcription, cell cycle, protein transport, nervous system development, and phosphorylation (Fig. 2f). Pathway analysis showed these miRNAs involvement in angiogenesis and metabolic pathways through regulation of cell mobility and vascular formation. Additionally, they targeted Rap1, Ras, and PI3K−Akt signaling pathways, as well as actin cytoskeleton regulation (Fig. 2g), suggesting potential roles in fibrosis modulation. Altogether, these analyses suggest that hUCMSC-EVs participate in inflammation regulation and tissue repair in pulmonary fibrosis.

### Protein composition of hUCMSC-EVs

**Fig. 3:**
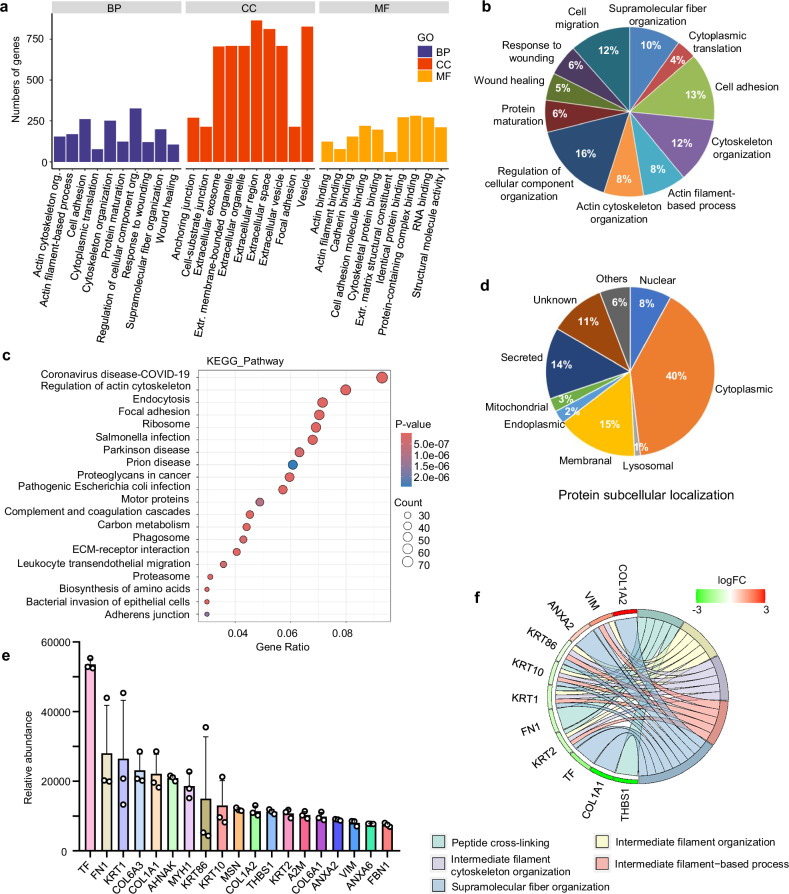
Protein composition of hUCMSC-EVs. **a** Gene clusters associated with the hUCMSC-EV proteins. BP: biological process, CC: cellular component, and MF: molecular function. **b** A gene ontology pie chart of biological process associated with the hUCMSC-EV proteins. **c** An enriched KEGG bubble chart targeted by the hUCMSC-EV proteins. **d** A pie chart of protein subcellular location of all proteins in the hUCMSC-EVs. **e** Relative abundance of 19 of extracellular proteins identified in the hUCMSC-EVs. **f** A gene ontology chord chart of biology process associated with the top 10 proteins

We next investigated whether proteins contribute to the functions of hUCMSC-EVs by performing a proteomic analysis. A total of 1409 proteins were identified by LC-MS. GO analysis revealed their involvement in biological processes (BP), cellular components (CC), and molecular functions (MF) (Fig. 3a, Supplementary Data 3). Key functional categories included cellular component organization, wound healing, cell adhesion, and supramolecular fiber organization (Fig. 3b). KEGG analysis showed enrichment in pathways such as actin cytoskeleton regulation, endocytosis, and focal adhesion (Fig. 3c). We further classified the proteins by subcellular localization: 40% were cytoplasmic, 15% membrane-associated, and 14% secreted (Fig. 3d). To identify the core functional proteins, we focused on those comprising over 80% of the total protein content, yielding 19 highly abundant proteins (Fig. 3e). Notably, many of these proteins were involved in supramolecular fiber organization (COL1A2, VIM, ANXA2, TF, COL1A1, FN1) and cytoskeleton organization (KRT1, COL6A3, COL1A1, KRT86, KRT10) (Fig. 3f). Given that most of them are extracellular matrix components associated with fibrosis, our findings suggest that hUCMSC-EVs may deliver functional proteins that contribute to tissue remodeling and regulation of targeted cell behavior.

### Metabolomics analysis of hUCMSC-EVs

**Fig. 4:**
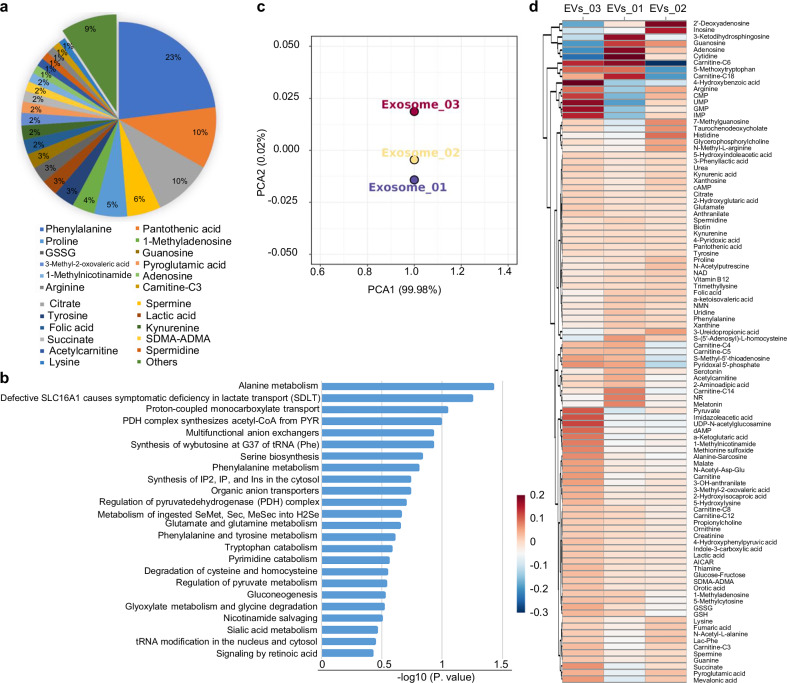
Metabolomics analyses of hUCMSC-EVs. **a** The pie chart shows the proportion of all metabolites. **b** The bar chart represents the metabolomics functional analysis, showing the metabolic pathways involving EVs metabolite **c** A PCA plot displays the distribution of three samples across the first two principal components (PCA1 and PCA2), which explain 99.98% and 0.02% of the variance, respectively. Each point represents a single sample. **d** A heatmap represents the correlation among three samples

To address whether other components contribute to the function of hUCMSC-EVs, we analyzed their metabolic profile. MS analysis identified 104 metabolites (Fig. 4a, Supplementary Data 4), with the top 23 accounting for 91% of the total abundance, while the remaining 89 were present at low levels (Fig. 4a, green section). Functional annotation revealed enrichment in amino acid metabolism, including glutamine metabolism and gluconeogenesis (Fig. 4b). To evaluate the consistency of hUCMSC-EVs preparations, we performed Principal Component Analysis (PCA) using three independent batches. PCA1 values from three batches were nearly identical, and PCA2 values showed only minor variation, indicating high reproducibility (Fig. 4c). Heatmap analysis further confirmed that metabolite composition across the three batches remained highly similar (Fig. 4d). Together, these findings indicate that hUCMSC-EVs carry a defined set of metabolites enriched in key metabolic pathways and exhibit high batch-to-batch consistency. These results support the quality and stability of our hUCMSC-EVs preparation and suggest that metabolites may contribute to their biological function.

### Bio-distribution of nebulized hUCMSC-EVs

**Fig. 5:**
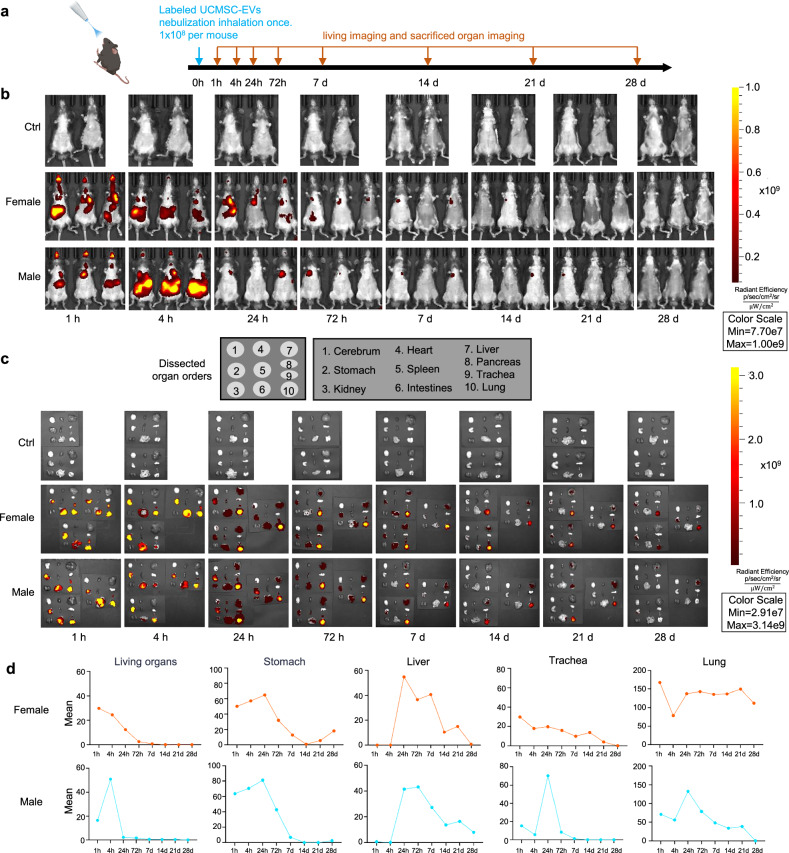
Bio-distribution of nebulized hUCMSC-EVs. **a** A schematic representation of the animal experiments is provided. On day 1, mice were administered intratracheally with labeled UCMSC-EVs (1×10^8^ per mouse, *n* = 72). The living imaging and sacrificed organ imaging were collected according to the time point. The image was created using MedPeer. **b** Biodistribution of DiR-labeled hUCMSC-EVs in vivo continuously until 28 d post-nebulization. **c** Biodistribution of DiR-labeled hUCMSC-EVs in vitro continuously until 28 d post-nebulization. The list of terms represents the layout of isolated organs. **d** Statistical curves depicting the time-dependent distribution of signals from both in vivo and ex vivo imaging. Data include separate analyses for male and female groups, as well as signal distribution across different organs

To search for the application of hUCMSC-EVs in the treatment of lung diseases, we determined to use nebulization administration. For this purpose, we traced the distribution of hUCMSC-EVs labeled with fluorescent dye (1×10^8^ particles per mouse) in C57BL/6 mice after nebulization inhalation over a period of 28 days (Fig. 5a). A whole body fluorescent examination showed that hUCMSC-EVs entered into the chest and abdomen within 1 h and reached at the highest level at 4 h (Fig. 5b). Detailed dissection of the organs showed that hUCMSC-EVs distributed in the trachea, lung, stomach, intestines, and liver but not in the cerebrum, heart, spleen, and kidney (Fig. 5b). Interestingly, we observed that the hUCMSC-EVs was concentrated on the lung, stomach and liver at 24 h after nebulization inhalation (Figs. 5c, d, 24 h). It appeared that the fluorescent signal gradually became faint after 72 h and diminished at day 21 for female and day 28 for male (Fig. 5d). However, the signal was detectable in the lung at day 28 when analyzed in the dissected organs (Fig. 5c, d). All these results suggest that hUCMSC-EVs enter into the body and are mainly maintained in the lung after inhalation.

### Safety and efficacy of nebulized hUCMSC-EVs in preventing injury-induced lung fibrosis in mice

**Fig. 6:**
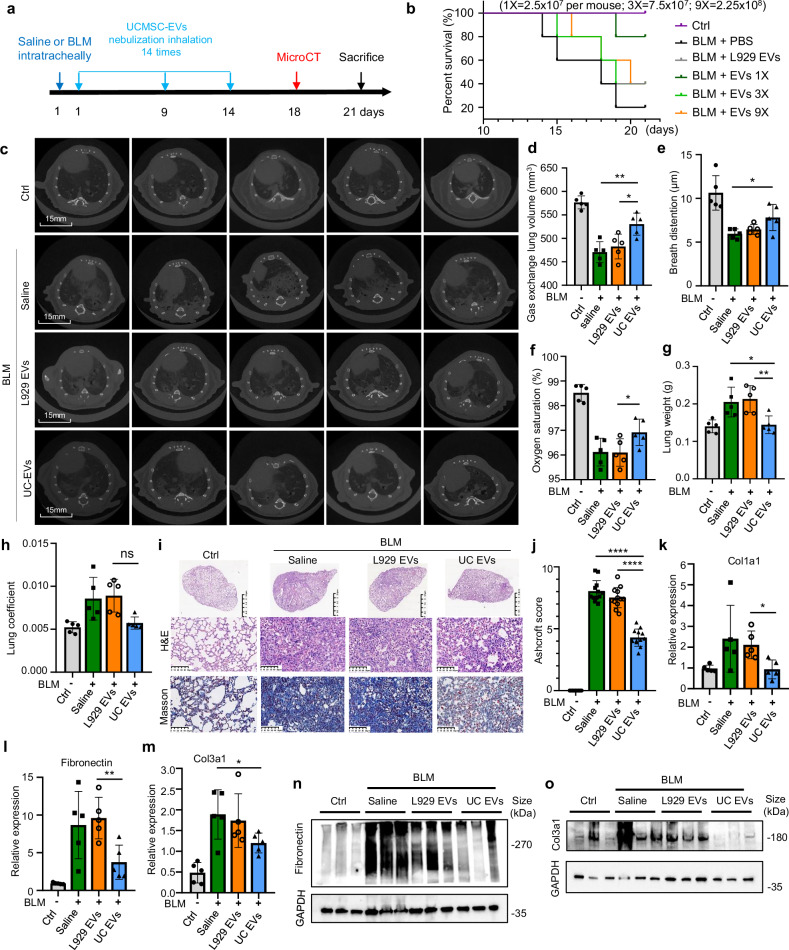
Efficacy of nebulized hUCMSC-EVs in treating injury-induced lung fibrosis in mice. **a** A schematic representation of the animal experiments is provided. On day 1, mice were administered intratracheally with bleomycin (BLM; 1.5/2.5 mg/kg body weight) or an equivalent volume of saline solution. After 2 h, three groups of mice challenged with BLM underwent nebulization with hUCMSC-EVs at three different doses (2.5 × 10^7^, 7.5 × 10^7^, 2.25 × 10^8^ particles), while one group of BLM-challenged mice and the control group (without BLM challenge) received the same volume of saline treatment. Mice were randomly assigned to 5 groups (*n* = 5 per group). **b** Kaplan-Meier survival curves of mice receiving different interventions (BLM; 2.5 mg/kg body weight). **c** Micro-CT images of lungs from mice in the experiment. The images were taken at 18th day post-injury. (BLM; 1.5 mg/kg body weight, UC-EVs represents 2.5 × 10^7^, 1 × EVs) **d** Lung volumes evaluated based on the three-dimensional reconstruction data from micro-CT; *n* = 5, p values were calculated. **e** Breath distention and **f** Oxygen saturation levels were measured by the MouseOx Small Animal Vital Signs Monitor. P values were calculated by two two-tailed t-test; *n* = 5. **g** The lung weight histogram depicts the weight of entire lung tissue in each group. **h** The lung coefficient was determined as wet lung weight (g) divided by total body weight (g). **i** Representative histological lung sections from mice at 21 days post-injury stained with H&E and Masson’s trichrome. Scale bars, 500 μm or 50 μm. **j** Quantitative evaluation of fibrotic severity with the Ashcroft score in lungs of mice receiving different interventions. The Ashcroft scores were calculated based on the H&E staining. The severity of fibrotic alterations in each section was assessed as the mean score in the observed microscopic fields. Ten fields per section were selected and the scores were marked by two evaluators and averaged as the final values. Quantitative PCR analyses for the relative mRNA levels of Col1a1 (**k**), Fibronectin (**l**) and Col3a1 (**m**), in the lung tissues of mice. Western blots of Fibronectin (**n**) and Col3a1 (**o**) in the lungs (*n* = 3). GAPDH was used as a loading control. Molecular weight was labeled

To evaluate the safety and efficacy of nebulized hUCMSC-EVs for pulmonary fibrosis, we conducted a preclinical study in mice. Different dosages (2.5 × 10^7^, 7.5 × 10^7^, and 2.25 × 10^8^ particles) of nebulized hUCMSC-EVs were administered to bleomycin (BLM)-induced (2.5 mg/Kg) C57BL/6 mice 14 times over two weeks, followed by a micro-CT scan on 18^th^ day (Fig. 6a). Safety assessments (Supplementary Data 5) included ALT, AST, Glu, HDL, TC, BUN, CREA, Hb, LDL, TG, LYM, NEUT, PLT, RBC, WBC, and IL-6 (Supplementary Fig. 2a–2p, Supplementary Data 5). ALT was most affected by bleomycin treatment, while LYM, NEUT, and WBC showed trends toward normalization after hUCMSC-EV therapy. Other parameters showed no significant changes, and no adverse effects were observed. For efficacy, Kaplan-Meier survival analysis demonstrated that hUCMSC-EVs (2.5 × 10⁷ particles per mouse) significantly improved survival rates and median survival time in bleomycin-induced lung injury (Fig. 6b). Next, in a preventive model using the optimal dose (1x) (2.5 × 10⁷ particles per mouse) micro-CT analysis showed reduced tissue density, bronchiectasis, and septal thickening in mice treated with hUCMSC-EVs compared to controls (saline and L929-EVs) (Fig. 6c). Importantly, nebulized hUCMSC-EVs effectively halted fibrosis progression, as evidenced by a marked reduction in the fibrotic lesion area (Fig. 6c). Furthermore, three-dimensional reconstruction analyses based on micro-CT images revealed a marked decrease in lung volume following BLM challenge (Fig. 6d, column 2 vs. 1), which was substantially restored by nebulized hUCMSC-EV treatment. Moreover, three-dimensional reconstruction of micro-CT images revealed a significant decrease in lung volume post-BLM challenge, which was largely restored by hUCMSC-EV treatment (Fig. 6d). Additionally, improvements in breath distention (Fig. 6e) and oxygen saturation (Fig. 6f) were observed. Lung weight (Fig. 6g) and lung coefficient (Fig. 6h) also improved after therapy. Histological analyses using H&E staining revealed that hUCMSC-EVs alleviated BLM-induced pneumonic lesions, including loss of alveolar architecture, septal thickening, enlarged alveoli, and increased inflammatory cell infiltration. Nebulized hUCMSC-EVs significantly reduced these pathological changes (Fig. 6i, H&E). Statistical analysis showed a significant decrease in the Ashcroft score (Fig. 6j), and Masson staining demonstrated reduced collagen accumulation in the lungs after hUCMSC-EV treatment (Fig. 6i, bottom). Furthermore, hUCMSC-EV treatment decreased Col1a1, Fibronectin, and Col3a1 expression (Fig. 6k–m), with reduced fibronectin and Col3a1 levels observed at the protein level (Fig. 6n, o). These data suggest that nebulized hUCMSC-EVs have therapeutic potential for pulmonary fibrosis.

### The role of hUCMSC-EVs on the therapy of lung fibrosis in mice

**Fig. 7:**
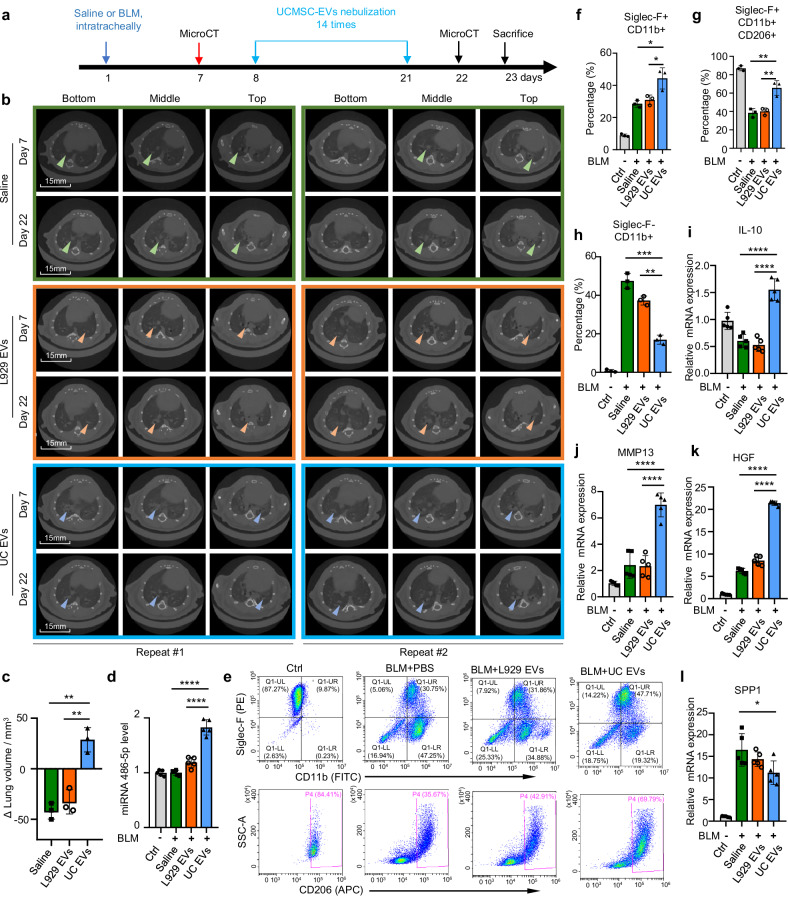
The role of EVs on the therapy of lung fibrosis. **a** A schematic representation of the animal experiment. On Day 1, mice received an intratracheal administration of bleomycin (BLM; 1-2 mg/kg body weight). On Day 7, all mice underwent micro-CT imaging and were randomly assigned to three groups (*n* = 3 per group). On Day 8, mice were treated via nebulization with hUCMSC-EVs or L929-EVs (2.5 × 10⁷ particles) or an equivalent volume of saline solution. On Day 22, micro-CT imaging was performed again to assess fibrosis changes at the same anatomical locations before and after treatment. **b** Representative micro-CT images on the same cross-section of lungs from all mice at the 7th day post-injury and at the 22th day after BLM-challenge. Two repeats were presented. **c** An illustration of the alterations in lung volumes observed in response to therapeutic interventions. Lung volumes were calculated both before and after treatment. A negative value indicates a decrease in lung volume post-treatment. (*n* = 3). **d** Stem-loop PCR analysis of the relative mRNA levels of miR-486-5p in mouse lung tissues. **e** Representative flow cytometry pseudo color plots showing expression of Siglec-F, CD11b, and CD206 in alveolar macrophages from different groups of mice. **f** Proportions of Siglec-F+, CD11b+ macrophage from bronchoalveolar lavage fluid. **g** Proportions of Siglec-F+, CD11b+ and CD206 macrophage from bronchoalveolar lavage fluid. **h** Proportions of Siglec-F-, CD11b+ macrophage from bronchoalveolar lavage fluid. Quantitative PCR analyses for the relative mRNA levels of IL-10 (**i**), MMP13 (**j**), HGF (**k**), and SPP1 (L) in the alveolar macrophages of mice

To investigate whether hUCMSC-EVs could mitigate the fibrotic process, we treated mice of BLM-induced fibrosis, covering both the proliferative and fibrotic phases. In such a case, 1-2 mg/kg of BLM was administered and the fibrosis onset was confirmed through micro-CT imaging on day 7 (Fig. 7a). Based on fibrosis severity, mice were randomly categorized into mild, moderate, and severe fibrosis groups, and subsequently assigned to three treatment conditions: saline nebulization, L929-EVs nebulization, or hUCMSC-EVs nebulization. From day 8 to 21, mice received 14 rounds of treatments, followed by a micro-CT scan on day 22 to assess fibrosis progression (Fig. 7b). The results showed that the fibrosis continued to progress in BLM-challenged mice treated with saline and L929-EVs (Fig. 7b, green and orange boxes). Intriguingly, mice treated with hUCMSC-EVs showed significant reduction in fibrosis (Fig. 7b, blue boxes). Notably, fibrotic lesions in different lung regions, including basal, middle, and apical sections, were markedly reduced with hUCMSC-EV treatment, a trend consistently observed across experimental replicates (Fig. 7b). Three-dimensional reconstruction revealed that while the PBS-treated group exhibited further lung volume decline, the MSC-EV-treated group displayed a substantial lung volume increase (Fig. 7c). These results suggest that nebulized hUCMSC-EVs effectively attenuate the progression of BLM-induced pulmonary fibrosis in mice.

To explore the mechanism underlying the therapeutic effects of hUCMSC-EVs, we analyzed miRNA expression in lung tissues and identified significant upregulation of miRNA-486-5p in the hUCMSC-EV-treated group (Fig. 7d). As miRNA-486-5p was reported to suppress the inflammatory responses by targeting macrophages,^36,37^ which are crucial in pulmonary fibrosis,^38^ we further investigated the involvement of alveolar macrophages in the response to hUCMSC-EVs. Bronchoalveolar lavage (BAL) was collected from mice challenged with BLM and treated with EVs and analyzed via flow cytometry. We observed increased levels of alveolar mononuclear cell populations, as indicated by Siglec-F+/CD11b+ cells,^39^ in all the groups of mice following BLM challenge compared with that in the healthy mice (Fig. 7e, top panel, Ctrl vs BLMs; Fig. 7f). While the total number of alveolar mononuclear cells remained high after hUCMSC-EV treatment (Figs. 7e, 30.75%, 31.86% and 47.71% vs 9.87%; top right panel), the percentage of M2 macrophages (CD206+) increased significantly from 35.67~42.91% to 69.79% (Fig. 7e, bottom panel; Fig. 7g). Simultaneously, the percentage of M2 macrophages in healthy mice remained at 84.41%, but nebulized hUCMSC-EVs elevated the M2 macrophage population in the BLM-challenged mice (Fig. 7e–g). On the other hand, nebulized hUCMSC-EVs dramatically decreased the Siglec-F-/CD11b+ cells (Fig. 7e, h). These results suggest that the role of hUCMSC-EVs on the fibrosis might be due to the elevated miRNA-486-5p and increased M2 macrophages.

To identify key genes expressed in macrophages associated with fibrosis, we performed qPCR analyses. The results showed that antifibrotic genes, including IL-10 (Fig. 7i),^40^ MMP13 (Fig. 7j),^41^ and HGF (Fig. 7k),^42^ were upregulated after hUCMSC-EV treatment. Additionally, SPP1, which has been reported to be upregulated in macrophages during fibrosis progression,^43,44^ was downregulated after hUCMSC-EV treatment (Fig. 7l). These findings indicate that BLM-induced lung injury leads to macrophages recruitments to the alveoli, and that hUCMSC-EV treatment promotes polarization of these macrophages toward an M2 phenotype. In conclusion, nebulized hUCMSC-EVs effectively mitigate established pulmonary fibrosis by reducing fibrotic lesions, restoring lung volume, and modulating macrophage polarization. These results highlight the potential of hUCMSC-EVs as a novel therapeutic strategy for treating pulmonary fibrosis even after its onset.

### Clinical safety of nebulized hUCMSC-EVs on patients with pulmonary fibrosis

**Fig. 8:**
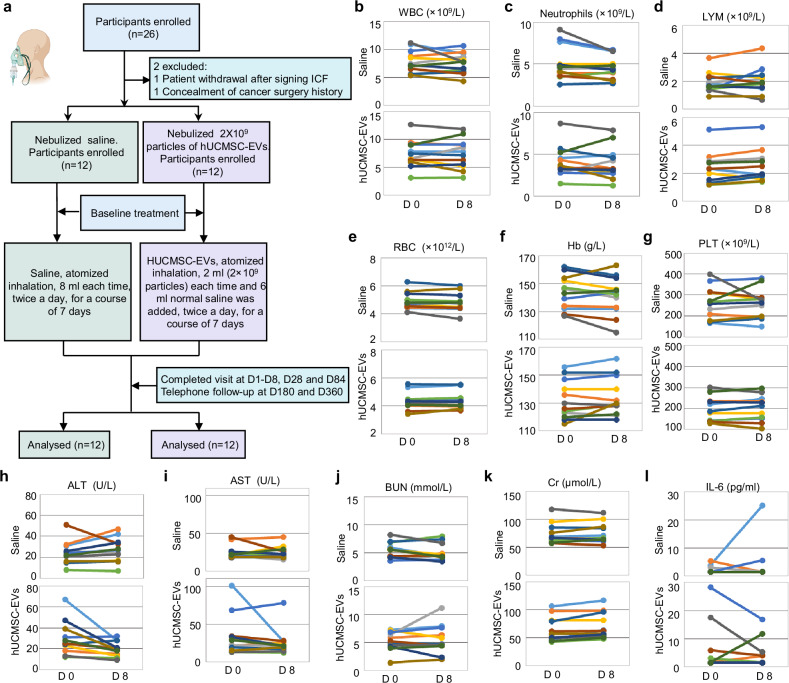
Clinical safety of nebulized hUCMSC-EVs on patients with pulmonary fibrosis. **a** A flow diagram for a phase I clinical trial. The image was created using MedPeer. Laboratory examinations of WBC (**b**), Neutrophils (**c**), LYM (**d**), RBC (**e**), Hb (**f**), PLT (**g**), ALT (**h**), AST (**i**), BUN (**j**), Cr (**k**), IL-6 (**l**) before and after the treatment with nebulized saline and hUCMSC-EVs in the clinical trial were performed. WBC White Blood Cell. LYM Lymphocyte. RBC: red blood cell. Hb Hemoglobin. PLT Platelet. ALT Alanine Aminotransferase. AST Aspartate Aminotransferase. BUN Blood Urea Nitrogen. Cr Creatinine. IL-6 Interleukin-6

To address whether nebulized hUCMSC-EVs have any effect on patients with pulmonary fibrosis, we initiated a clinical trial (MR-46-22-004531, ChiCTR2300075466) from April 20, 2023, to May 2, 2025. A total of 26 participants were screened for eligibility; two were excluded-one due to withdrawal and the other for concealing a history of cancer surgery following the inclusion and exclusion criteria (Supplementary Text). Ultimately, 24 participants, diagnosed with pulmonary fibrosis lesions by HRCT (high-resolution CT), were enrolled in this single-blind trial. These participants were randomized into two groups: one receiving routine treatment plus nebulized hUCMSC-EVs (experimental group, 12 patients), and the other receiving saline nebulization (control group, 12 patients) (Fig. 8a). The baseline characteristics of these 24 patients were summarized and no significant difference was observed between the experiment group and saline group (Table 2, Supplementary Table 1). All patients maintained their original treatment regimens throughout the study (Supplementary Table 2). Nebulization consisted of 6 ml saline combined with 2 ml of hUCMSC-EVs (2 × 10^9^ particles) or saline solution (control group). Nebulization was administered twice daily, with the treatments given at 09:00 ± 30 minutes and 20:00 ± 30 minutes for 7 days. The mesh nebulizer used ensured particle diameters < 5 μm. Follow-up assessments were performed on days 1, 8, 28, and 84, with telephone follow-ups at days 180 and 360 (Fig. 8a, Supplementary Table 3).

**Table 2:** Baseline characteristics of the participants

Serial number	Age	Gender	BMI	Allergy history	Smoking history	Disease duration	Comorbidities	Diagnosis
**001**	66	Male	26.675	None	1000	5 y +	None	COPD (Chronic Obstructive Pulmonary Disease.) with pulmonary fibrosis
**002**	65	Male	21.231	None	none	1 y +	Hyperlipidemia	Post-inflammatory pulmonary fibrosis
**003**	65	Female	27.055	None	none	10 y +	Bronchiectasis, hyperlipidemia	Emphysema with pulmonary fibrosis
**004**	69	Male	23.588	None	1040	9 m +	Rheumatic heart disease, post-mitral valve replacement, cerebral infarction, chronic gastritis	Emphysema with pulmonary fibrosis
**005**	71	Male	19.047	None	80	5 y +	None	COPD with pulmonary fibrosis
**006**	65	Male	24.655	None	300	1 y +	Inactive pulmonary tuberculosis	Idiopathic Pulmonary Fibrosis (IPF)
**007**	55	Male	21.755	None	none	5 y	Splenectomy	Interstitial lung disease (ILD)
**008**	60	Female	20.202	None	none	3 y	Bronchiectasis, type 2 diabetes, hyperlipidemia, hypertension, gallstones	COPD with pulmonary fibrosis
**009**	69	Male	20.703	None	none	9 m +	Bronchiectasis, non-tuberculous mycobacterial lung disease, COVID-19	Post-inflammatory pulmonary fibrosis
**010**	63	Male	24.167	None	600	2 y +	Bronchiectasis, hypertension, hyperlipidemia	COPD with pulmonary fibrosis
**011**	58	Female	29.997	None	none	3 y +	Type 2 diabetes, hypertension, hyperlipidemia, hyperuricemia, arteriosclerosis, fatty liver, thyroid nodules	Interstitial lung disease (ILD)
**012**	76	Male	27.513	None	600 +	6 y +	Hyperlipidemia, fatty liver, pulmonary nodules	COPD with pulmonary fibrosis
**013**	58	Female	21.228	None	none	1 y +	Anti-synthetase syndrome	Interstitial lung disease (ILD)
**014**	53	Female	24.524	None	none	2 y +	Anti-synthetase syndrome	Interstitial lung disease (ILD)
**015**	79	Male	24.741	None	800	1 y +	COPD	Post-inflammatory pulmonary fibrosis
**016**	69	Female	20.408	None	none	8 y	Scleroderma	ILD
**017**	65	Male	21.231	None	400 +	1 y +	None	COPD with pulmonary fibrosis
**018**	64	Male	23.384	None	600 +	1 y +	COPD	Post-inflammatory pulmonary fibrosis
**019**	69	Male	18.508	None	1000	1 y +	None	COPD with pulmonary fibrosis
**020**	65	Female	22.959	None	none	3 y +	ANCA-associated vasculitis	ILD
**021**	68	Female	23.873	None	none	5 y +	Polymyositis	ILD
**022**	56	Male	22.833	None	800	1 y	Hypertension	COPD with pulmonary fibrosis
**023**	49	Male	18.218	None	800	1 y +	Arrhythmia, coronary artery sclerosis, alcoholic liver disease, cholestasis, chronic gastritis, duodenal bulb inflammation	COPD with pulmonary fibrosis
**024**	57	Male	15.822	None	1000	2 y +	History of left upper lobe pulmonary bullae surgery	COPD with pulmonary fibrosis

The priority objective was to evaluate the safety of nebulized hUCMSC-EVs. All participants tolerated the treatment well, with no significant changes observed in cardiac function, hematological parameters, liver or renal function, acute allergic reactions, or immune responses. During the initial investigation, body temperature, electrocardiograms (ECG), dermatological assessments, and oxygen saturation (data kept in the hospital) were monitored. These parameters remained constant within normal ranges, with no allergic skin reactions, arrhythmias, or ST-T segment abnormalities during the trial. Oxygen saturation remained stable throughout the treatment period (Supplementary Fig. 3a, Supplementary Table 4).

Next, we performed a serum examination for the participants on day 0 and 8 after the inhalation. The results showed that the hematological index, including white blood cell count (WBC), neutrophils (N), lymphocytes (LYM), red blood cells (RBC), hemoglobin (Hb), and platelets (PLT), remained of no significant change from day 0 to day 8 during the treatment (Figs. 8b–g, Supplementary Table 5). Liver function markers, including alanine aminotransferase (ALT) and aspartate aminotransferase (AST), as well as renal function markers like blood urea nitrogen (BUN) and creatinine (Cr), remained stable (Figs. 8h–k, Supplementary Table 5). Constantly, we observed that blood lipids (TG, triglycerides; TC, total cholesterol; HDL, high-density lipoprotein; and LDL, low-density lipoprotein) (Supplementary Fig. 3c–e, Supplementary Table 5) and blood glucose (Glu) (Supplementary Fig. 3f) remained within the normal range after the first week of inhalation. Furthermore, we found that IL-6 was not altered after the application of nebulized hUCMSC-EVs (Fig. 8l, Supplementary Table 6), suggesting that the treatment induces no inflammation. All these observations consistently indicate that the treatment with nebulized hUCMSC-EVs is safe in humans.

In a long-term observation after the treatment, we further recorded the potential adverse events for the patients. Reassuringly, no allergic reactions to hUCMSC-EVs were reported, and no adverse events were observed during the follow-up visits (for 12 months). Overall, we concluded that the nebulized hUCMSC-EV treatment for patients with pulmonary fibrosis was well tolerated and demonstrated a robust safety profile.

### Clinical efficacy of nebulized hUCMSC-EVs in the therapy of pulmonary fibrosis

**Fig. 9:**
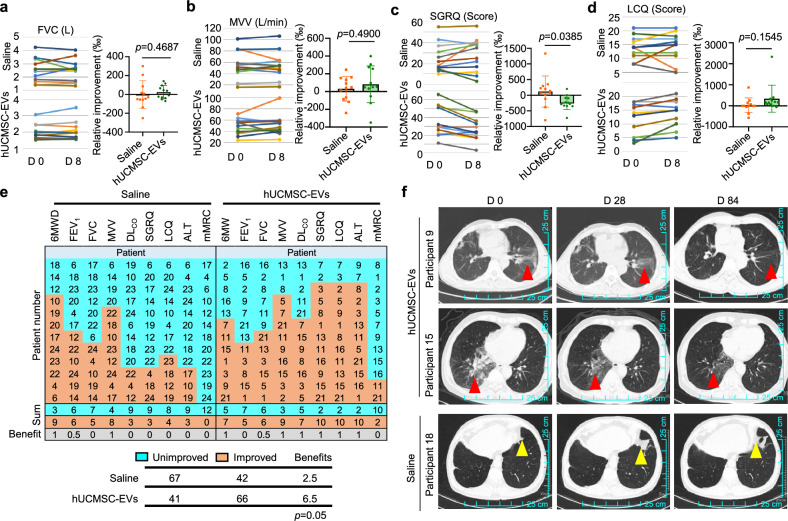
Clinical efficacy of nebulized hUCMSC-EVs in the therapy of pulmonary fibrosis. Levels and statistical analyses of FVC (**a**), MVV (**b**), SGRQ (**c**), and LCQ (**d**) before and after the treatment with nebulized saline and hUCMSC-EVs in the clinical trial. **e** A heatmap displaying the benefit cases from the treatment with nebulized saline and hUCMSC-EVs. Orange color indicates improvement, while blue color indicates no improvement. The numbers correspond to the case identifiers. (1: improved > unimproved; 0.5: improved = unimproved; 0: improved < unimproved) **f** Representative HRCT images of the lungs of three participants. FVC Forced Vital Capacity, MVV Maximal Voluntary Ventilation, SGRQ St. George’s Respiratory Questionnaire, LCQ Leicester Cough Questionnaire

To demonstrate the efficacy of the therapy in patients with pulmonary fibrosis, we analyzed the lung function indexes. The results showed that the forced vital capacity (FVC) (Fig. 9a, Supplementary Table 7) and maximal voluntary ventilation (MVV) (Fig. 9b, Supplementary Table 7) were significantly improved in the patients treated with nebulized hUCMSC-EVs compared with the patients treated with saline. However, other indexes showed no difference between the experimental group and control group but with no trend of aggravation after the treatments (Supplementary Fig. 4, Supplementary Tables 7, 8). Furthermore, we used widely recognized questionnaires to assess lung function recovery.^45^ The results showed that the St. George’s Respiratory Questionnaire (SGRQ) scores were significantly decreased in patients in the experimental group compared to that in the control group, indicating that the patients were recovered with reduced activity limitations after the additional nebulized hUCMSC-EV therapy (Fig. 9c, Supplementary Table 9). On the other hand, we observed that the Leicester Cough Questionnaire (LCQ) scores, which reflect improved quality of life for patients when increased, were increased in the patients under the nebulized hUCMSC-EV treatment (Fig. 9d, Supplementary Table 9). To evaluate the overall effect of the therapy, we summarized all the indexes with alterations after the treatment. These indexes included 6 min walk distance (6MWD), FEV_1_, FVC, MVV, DL_CO_, SGRQ, LCQ, ALT, and mMRC. We categorized the patients into improved or unimproved group by comparing the alteration of the indexes before and after the nebulized hUCMSC-EV treatment. Finally, we calculated the benefit events which were defined by the improved over unimproved parameter (1: improved > unimproved; 0.5: improved = unimproved; 0: improved < unimproved). We performed a paired T-test based on the indexes and obtained p = 0.05 (Fig. 9e). This result suggests that the overall responses of the nebulized hUCMSC-EV therapy based on the routine treatment is significantly beneficial for the patients.

Finally, we analyzed the HRCT images for all the patients examined at day 0, 28 and 84 after the treatment and compared the alterations during the observation. The result showed that most of the patients remained of no significant improvement and with no aggregation (data not shown but kept in the information center of the hospital). This is consistent with the routine clinic treatment for the patients.^46^ Interestingly, we observed that two patients had a significant improvement in the HRCT images, as observed on day 28 after the nebulized hUCMSC-EV treatment. These two patients showed a further improvement, as observed on day 84 after the treatment with nebulized hUCMSC-EVs (Fig. 9f, top two panels, red arrows). In detail, participant 9 exhibited patchy opacities and ground-glass density in the left lung with subpleural band-like blurry shadows on day 0 and this CT image feature was ameliorated partially at day 28 but significantly at day 84, as demonstrated by largely resolved lesions of the left lung (Fig. 9f, top row). Participant 15 demonstrated an extensive consolidation and uneven density in the right lower lobe at day 0, accompanied by scattered patchy ground-glass density, linear shadows, and linear ground-glass density in the left lung oblique fissure. On day 28, the right lower lobe consolidation had mainly resolved, with the disappearing surrounding ground-glass density and only residual and delicate shadows in reticular, linear, and ground-glass shapes. The ground-glass shadow in the left lung oblique fissure had also mostly resolved. On day 84, the consolidation in the right lower lobe was predominantly resolved in this patient (Fig. 9f, middle row). As a control, we showed that patient 18 in the control group (routine treatment plus saline) had an exacerbated progression of fibrosis during these periods (Fig. 9f, bottom panel). In summary, all these observations suggest that treatment with nebulized hUCMSC-EVs is beneficial for patients with pulmonary fibrosis during their routine treatment. Therefore, we conclude that nebulized hUCMSC-EV therapy showed preliminary efficacy in clinical application.

## Discussion

The therapy of pulmonary fibrosis remains a significant challenge as this disease is still arising, particularly in the case of pandemic infections. The current clinic treatment is to manage the disease with multiple strategies to maintain the patients in a better condition without progression or aggravation. In this study, we reported to use nebulized hUCMSC-EVs, which have emerged as a promising avenue for cell-free therapy,^47,48^ as a new strategy for the therapy of pulmonary fibrosis patients. We have established a comprehensive system to ensure compliance with Good Manufacturing Practice (GMP) criteria at each stage of manufacturing and proposed 4 Critical Quality Control Points (CQCPs) to guarantee the quality. Our results showed that nebulized hUCMSC-EV treatment was safe, and the patients benefited from the treatment as evaluated by several clinical indexes. Because our trial was based on routine baseline treatment, we concluded that the nebulized hUCMSC-EV therapy further improved the patient status and could be combined with current treatments. This opens a new approach to the therapy of pulmonary fibrosis and brings a hope for pulmonary fibrosis patients to have a better recovery in the future.

Several studies have delved into the utilization of nebulized EVs for the treatment of pulmonary diseases. Weixia Xuan et al. reported that nebulized platelet-derived extracellular vesicles effectively mitigated chronic cigarette smoke-induced emphysema in mice.^49^ Meng-meng Shi et al. reported preclinical efficacy and clinical safety of clinical-grade nebulized allogenic adipose mesenchymal stromal cells-derived extracellular vesicles.^31^ Phuong-Uyen C. Dinh et al. presented a study utilizing lung spheroid cell-secretome (LSC-Sec) and exosomes (LSC-Exo) by inhalation to treat lung injury and fibrosis in mouse models.^32^ In this study, we systematically investigated the therapeutic effects of hUCMSC-EVs at different concentrations using a micro-mist nebulizer in preclinical animal experiments. All results demonstrated significant improvements in lung tissue architecture and functions following nebulized administration of hUCMSC-EVs in a preclinical mouse model of pulmonary fibrosis. Micro-CT analysis revealed a reduction in fibrotic lesions, bronchiectasis, septal thickening, and fibrotic dash area in mice treated with hUCMSC-EVs compared to those receiving saline or DMEM alone. Our results in the animal model consolidated the role of hUCMSC-EVs in the therapy of pulmonary fibrosis. Consistently, our study validated the previous studies for the use of nebulized EVs in treating other diseases such as COVID-19 pneumonia.^33,50,51^ However, as the content of the exosomes or extracellular vesicles varied quite differently, a critical question for successfully translating these particles into clinical practice remains challenging. In this study, we established a rigorous procedure to produce hUCMSC-EVs using a GMP standard and proposed several quality control criteria using MS, RNA-deep seq, and metabolite analyses. Our study also matched the requirements as others addressed that the production process of hUCMSC-EVs should be monitored during the isolation and purification from mesenchymal stem cells, as well as the formulation for therapeutic usage.^52^ As there is no way to avoid the heterogeneity,^53^ we considered that analyzing the contents is a method to guarantee the batch-to-batch stability.

For the mechanism, we analyzed the contents of RNAs, proteins, and metabolites. We observed that 10 miRNAs with an individual amount of over 2% were dominant and accounted for 62% of the total miRNAs. Our results echoed the study from Scott W Ferguson et al., where, miR-451a, miR-221-3p, miR-21-5p, miR-100-5p, and let-7a-5p were mutually identified, which accounts for 32% of the total mRNAs in our analysis and 22.7% in their report.^54^ These 5 miRNAs might indicate the main functions of the hUCMSC-EVs. Indeed, miRNA-451a, the most enriched miRNA in hUCMSC-EVs, has been reported as a transcriptional regulator of several fibrosis-associated genes.^55^ Our study supports the fact that MiR-451a-transfected EVs significantly alleviated fibrogenesis in PHMG-p-exposed lungs,^55^ highlighting a potential therapeutic mechanism of hUCMSC-EVs in fibrosis treatment. On the other hand, the protein contents of hUCMSC-EVs were quite different in our results from others. Recently, Youkun Bi et al.^56^ and Xiao Xu et al.^57^ reported the proteins in hUCMSC-EVs. Although the GO analysis revealed several overlapping pathways from the proteins, there were rare common proteins in the hUCMSC-EVs between their results and ours. To our surprise, we only observed COL6A3, MYH9, and FN1 mutually presented ours and the result from Xiao Xu et al.^57^ In this context, we consider that the protein types and amounts might be more sensitive, as affected by environmental factors such as temperature, pH, and freeze-thaw cycles,^58^ and storage conditions.^59^ In addition, we found that about 30 components of the metabolites in our analyses were reported by others.^60^ Our study demonstrated that the purified hUCMSC-EVs maintained relatively high consistency and stability among three batches of productions, purifications, and storages in the metabolites. We speculate that the different proteins and metabolites reported by different groups might be due to the different cells isolated from different donors. Taken together, we used proteins, metabolites, and miRNAs to evaluate the consistency and stability of the purified hUCMSC-EVs.

Another concern about the application of the hUCMSC-EVs is the dosage. Unlike chemical drugs,^61^ the dosing strategies of biological agents typically have a broader therapeutic window.^62^ In our study, we used 2.5 × 10^7^ particles per mouse as a basic dosage. We then increased the dosages to 2.25 × 10^8^ particles per mouse, with a 9-fold increase, as the maximal administration amount. We observed that this maximal amount of hUCMSC-EVs had no adverse effect in mice. On the other hand, we observed that the amount of 2.5 × 10^7^ particles per mouse remained the best therapeutic effect in the mouse model among other higher dosages. Considering all these observations, we determined to use 2 × 10^9^ particles per person, calculated according to the minimal amount of 2.5 × 10^7^particles per mouse.

Furthermore, our data support the clinical efficacy of nebulized hUCMSC-EV therapy in pulmonary fibrosis. In our clinical trial, we applied nebulized hUCMSC-EVs for patients under a routine baseline treatment. To our satisfaction, we observed that the patients who accepted the hUCMSC-EV treatment obtained more benefits in several indexes, particularly FEV_1_, FVC, MVV, and DL_CO_, than those under the routine treatment. These results suggest that hUCMSC-EVs have a complementary effect under the routine treatment for pulmonary fibrosis patients. Last but not least, we enrolled patients with four types of pulmonary fibrosis, including COPD with fibrosis, IPF, ILD, and post-inflammatory pulmonary fibrosis. Although no intergroup differences were observed across pulmonary fibrosis subtypes, two patients with post-inflammatory pulmonary fibrosis exhibited significant radiologic improvement following nebulized hUCMSC-EV therapy. In contract, we found that one patient in the control group suffered from the radiological progression of fibrosis in the lung. Interestingly, these alterations of HRCT were associated with changes in the parameters, as the two patients with HRCT improvement showed more benefit parameters than the patient with fibrosis progression. These results indicate that the treatment had an overall benefit for patients. Since the two patients with the HRCT improvement were the post-inflammatory pulmonary fibrosis, it is interesting to decipher if the hUCMSC-EV therapy is more suitable for such a population of pulmonary fibrosis patients. Overall, our results demonstrate that nebulized hUCMSC-EVs exhibit both safety and efficacy in treating patients with pulmonary fibrosis, suggesting a promising therapeutic strategy for this stubborn disease.

## Materials and methods

### Ethics approval and consent to participate

All experimental designs and protocols involving animals were approved by the Institutional Animal Care and Use Committee of Tsinghua University (Title of the approved project: Umbilical cord-derived mesenchymal stem cells preferentially modulate macrophages to alleviate pulmonary fibrosis. Approval Form ID: THU-LARC-2024-008. Date of IACUC approved: 2020/3/20) and complied with the recommendations of the academy’s animal research guidelines.

The experimental protocol was established, according to the ethical guidelines of the Helsinki Declaration and was approved by the Ethics Committee of Seventh Medical Center of Chinese PLA General Hospital (Title of the approved project: Umbilical cord and placental tissues and derivatives used in scientific studies of chronic inflammation in laboratory animals. Approval Form ID: 202200016. Date of IACUC approved: Jan 31, 2020). The patient(s) provided written informed consent for the use of samples.

The clinic trial was approved by the Ethics Committee of the First Affiliated Hospital of Hainan Medical University, Haikou 570102, Hainan, China (2022-KY-131, Title: A randomized, single-blind, placebo-controlled, phase I clinical study of the safety and efficacy of nebulized exosomes of hum an umbilical mesenchymal stem cells in the treatment of pulmonary fibrosis manifested by HRCT. Approved date: April 21, 2022), with registration numbers (MR-46-22-004531, and ChiCTR2300075466, Registration date September 6, 2023). Informed consent form was given to patients for participation in this trial.

### Primary cells and culture

Primary hUCMSCs were isolated from a healthy full-term human umbilical cord, which were collected following the guidelines of the Ethics Committee of the Seventh Medical Center of Chinese PLA General Hospital in Beijing, China, with a written informed consent from the donor. One donor was selected according to age, non-infection healthy status, firstborn and passage ability (over 10 passages). The hUCMSCs at passage 4 were expanded in a culture system with DMEM in an environment with 5% CO_2_ at 37 °C and passaged for 4 times when they reached about 80% confluence in dishes. The hUCMSCs were transferred into 10 cm^2^ culture flasks containing DMEM supplemented with 5% KOSR (knockout serum replacement), 1% Ultroser G, 1× L-glutamine, 1× NEAA (non-essential amino acid), 10 ng/mL bFGF (basic fibroblast growth factor), and 10 mg/L L-ascorbic acid for mass expansion.

### Mice

Male and female C57BL/6 mice of specific pathogen-free (SPF), aged 6 to 8 weeks, were obtained in Laboratory Animal Resources Center, Tsinghua University, Beijing, China. To ensure uniformity, both experimental and control mice were weight-matched within the range of 20 to 25 grams per mouse. The mice were housed at the Laboratory Animal Resources Center, Tsinghua University, maintained under SPF conditions with a room temperature between 20–24 °C and humidity levels between 35–55%, following a 12 h light and 12 h dark cycle. Mice had *ad libitum* access to food and water and underwent regular monitoring for overall health, fur condition, activity levels, and weight, in accordance with institutional protocols. Humane euthanasia via CO_2_ inhalation was administered at specified time points when necessary. The laboratory animal facility holds accreditation from the AAALAC (Association for Assessment and Accreditation of Laboratory Animal Care International), and all animal protocols utilized in this study were approved by the Institutional Animal Care and Use Committee (IACUC) of Tsinghua University. All samples were utilized in compliance with the approved standard experimental protocols set forth by the Animal and Medical Ethics Committee of Tsinghua University, Beijing, China.

### Isolation and characterization of hUCMSC-EVs

The hUCMSC-EVs were obtained from a cultural system we established according to a previous report.^63^ In brief, 20 pieces of microcarriers (3D TableTrix microcarriers W01 Cytoniche, Huankan, Beijing, China) were used and 8 × 10^6^ cells were inoculated to a reactor with 160 ml of serum-free culture medium. The reactor was set to rotate at 35 rpm for cultivation. Additional 80 ml of serum-free culture medium were added, and the reactor speed was increased to 40 rpm for cultivation after the second day of inoculation. A new set of 200 ml of serum-free culture medium was added, and the reactor speed was increased to 45 rpm at day 4. Another set of 100 ml of culture medium was added and the reactor speed was maintained at 45 rpm at day 5. Finally, 1.2 ×10^8^ cells and 540 ml culture supernatant were harvest after the bioreactor culture at day 7.

hUCMSC-EVs were isolated from the cell culture supernatant by a series of differential centrifugation. First, the collected cell culture supernatant was centrifuged at a low speed (800 *g*) for 5 min to pellet cells. The supernatant was then carefully removed to eliminate sediment. Subsequently, the supernatant was centrifuged at 2000 *g* for 10 min to remove dead cells. Finally, the supernatant was centrifuged at 10,000 *g* for 30 min to eliminate cell debris. After each centrifugation step, the sediment was discarded. The resulting supernatant contained hUCMSC-EVs, which were further purified by super-high speed centrifugation. Initially, centrifugation at 110,000 *g* for 1.5 h was conducted, followed by resuspension in 50 ml of PBS. Subsequently, another centrifugation at 110,000 *g* for 1.5 h was carried out to remove the supernatant. The hUCMSC-EVs were collected by resuspension in 10 ml of PBS. As a control, EVs from a mouse fibroblast cell line (NCTC clone 929) (L929-EVs) were prepared following the similar procedure.

Protein and RNA analyses were conducted on the obtained hUCMSC-EVs. Before use, all hUCMSC-EV samples underwent analyses for sizes using nanoparticle tracking protocol (Malvern Panalytical NTA Nanosight LM10) and for morphology using transmission electron microscopy (TEM). Subsequently, the samples were adjusted to the desired working concentrations for animal and clinical applications. A comprehensive system was established to guarantee compliance with Good Manufacturing Practice (GMP) criteria at each manufacturing stage, with certificated facility checking, personnel training, material control, environmental monitoring, and meticulous documentation management. The detailed critical quality control points (CQCP) at the critical process step were described in the text and the results were listed in supplementary information.

### Lung function assessment

Pulse distention, breath distention, and oxygen saturation levels were measured by the MouseOx Small Animal Vital Signs Monitor (STARR, USA) following the manufacturer’s instructions^64^ (https://www.starrlifesciences.com/).

### Micro-CT for mice

Micro-CT imaging was performed using a Quantum GX scanner (USA) according to the manufacturer’s instructions.^65^ The scans were conducted with a resolution of 4.5 μm, enabling high-precision imaging of the mouse lung structure. Imaging was carried out under a flow rate of 300–500 mL/min of oxygen, with 1–1.5% isoflurane concentration, to ensure minimal movement during scanning. The lung volume and other morphological features were quantified using 3D segmentation. Image processing was performed using the Caliper microCT Analyis Tools by Analyze (USA), where the lungs were manually or semi-automatically segmented from the surrounding tissues. The segmentation threshold was set based on standard protocols, with the analysis focused on identifying bone, airway, and alveolar regions to ensure accuracy. The analysis was performed for each mouse, with gas exchange lung volume calculated to assess the extent of injury and fibrosis.

### Design of a randomized, single-blind, and placebo-controlled phase l clinical trial

The safety and tolerance of nebulized hUCMSC-EVs were observed among pulmonary fibrosis participants in a phase I clinical trial, conducted as a single-center, 1:1 randomized, single-blinded, and placebo-controlled clinical trial. The trial was approved and conducted in the Clinical Research Center of the First Affiliated Hospital of Hainan Medical University (MR-46-22-004531) (ChiCTR2300075466), with scientific and ethical reviews following the Helsinki Declaration. All patients were enrolled at the First Affiliated Hospital of Hainan Medical University, China. The dosage was determined to be 2 × 10^9^ particles per person according to the optimal therapeutic dosage in mice (10^9^ particles per kilogram) estimated in our animal experiment. As we concluded the optimal dose was 2.5 × 10^7^ per mouse, we calculated the equivalence according to the scale transformation in small molecule drugs, which deduces the human dose to 1/40 of the mouse per kg. We took the average body weight of a human as 70 kg and a mouse as 0.025 kg. Therefore, the amount for human = 70 (kg, human body weight) x 2.5 × 10^7^ (the maximal amount per mouse (body weight: 25 g) ÷ 0.025 (kg/per mouse) ÷ 40 (factor from mouse to human) = 1.75 × 10^9^. Considering the practical operation, we set up the amount to 2 × 10^9^ particles per person. A total of 24 patients were randomly allocated into an experimental group (12 patients, nebulized hUCMSC-EVs) and a control group (12 patients, nebulized saline). The assessment includes patient vital signs, clinical symptoms and signs (St. George’s respiratory questionnaire, SGRQ; Leicester cough questionnaire, LCQ; modified medical research council dyspnea scale, mMRC), routine safety examinations (complete blood cell count, urinalysis, pregnancy test, infectious disease screening, blood biochemistry, tumor markers, electrocardiogram), physiological indicators (finger oxygen saturation, pulmonary function testing, 6MWD, 6-min walk distance), anatomical indicator (chest HRCT, high-resolution CT), acute exacerbation events (frequency and severity), and adverse events. All the examinations were performed in the hospital and the patient follow-up was scheduled as listed in Supplementary Table 9.

### Clinical trial procedures

The informed consent form was obtained after discussion with volunteers. The inclusion and exclusion criteria were set up before the trial. The patients’ basal information was recorded. hUCMSC-EVs were prepared by Shanghai Golden Well Cell Tissue Storage Company located in Beijing, following the protocol as described aforementioned and transported to the clinical site using a validated carrier (Huaxin Logistics Carriers, China) with a temperature of 2-8 °C. The solution of hUCMSC-EVs underwent a release inspection and was stored at 4 °C upon receipt. Before administration, the solution underwent another visual inspection at the clinical site. A safety assessment was conducted for the first volunteer in the first group before recruiting subsequent volunteers.

The trial was performed by patient screening, treatments, and follow-up observations. All patients were allowed to maintain their original routine treatment regimen and dosages. Patients in the experimental group received hUCMSC-EVs, and patients in the control group received saline. The total nebulization volume was prepared by diluting 2 ml of hUCMSC-EVs or saline solution (blank) with 6 ml of saline. The solutions were inhaled via nebulization twice daily (at 09:00 ± 30 min and 20:00 ± 30 min) for 7 days. The mesh nebulizer used was selected with a feature of nebulizing particles in a diameter of < 5 μm (TONETi Titanium YS38E nebulizer, China).

## Supplementary information

Supplementary Materials

Study protocol

Informed Consent Form 001

Informed Consent Form 002

Informed Consent Form 003

Informed Consent Form 004

Informed Consent Form 005

Informed Consent Form 006

Informed Consent Form 007

Informed Consent Form 008

Informed Consent Form 009

Informed Consent Form 010

Informed Consent Form 011

Informed Consent Form 012

Informed Consent Form 013

Informed Consent Form 014

Informed Consent Form 015

Informed Consent Form 016

Informed Consent Form 017

Informed Consent Form 018

Informed Consent Form 019

Informed Consent Form 020

Informed Consent Form 021

Informed Consent Form 022

Informed Consent Form 023

Informed Consent Form 024

Consent Form for Perinatal Tissue Donation

Ethics Approval for EVs

Phase I clinical registration

Ethics Committee of Seventh Medical Center of Chinese PLA General Hospital

INSTITUTIONAL ANIMAL CARE AND USE COMMITTEE FORM OF TSINGHUA UNIVERSITY

Original WB

RNA proportion of hUCMSC-EVs

All expressed miRNA

hUCMSC-EVs Protein

hUCMSC-EVs metablite

Mouse complete blood count and serum biochemical

## Data Availability

The data and materials supporting the findings of this study are available within the article or from the corresponding author upon reasonable request. The miRNA sequencing data have been deposited in the NCBI SRA with accession number SRR33363873. The protein sequencing data have been deposited in the ProteomeXchange with accession number PXD063385.
