# Correction: Clinical investigation on nebulized human umbilical cord MSC-derived extracellular vesicles for pulmonary fibrosis treatment

**DOI:** 10.1038/s41392-025-02293-w

**Published:** 2025-07-17

**Authors:** Meng Li, Huaping Huang, Xiaofei Wei, Huajuan Li, Jun Li, Bingchen Xie, Yuze Yang, Xingyue Fang, Lei Wang, Xiaona Zhang, Heyu Wang, Mengdi Li, Yuting Lin, Dezhi Wang, Yinyin Wang, Tongbiao Zhao, Jianqiu Sheng, Xinbao Hao, Muyang Yan, Lu Xu, Zhijie Chang

**Affiliations:** 1 State Key Laboratory of Membrane Biology, School of Medicine, Institute of Precision Medicine, Tsinghua University, Beijing, China; 2 Department of Respiratory Diseases, The First Affiliated Hospital of Hainan Medical University, Haikou, Hainan China; 3 Beijing cord blood bank, Beijing, China; 4 Jinfeng Laboratory, High-tech Zone, Chongqing, China; 5 School of Biomedical Engineering, Capital Medical University, Beijing, China; 6 Department of Hematology, The First Affiliated Hospital, Hainan Medical University, Haikou, Hainan China; 7 Medical School of Chinese PLA, Chinese PLA General Hospital, Beijing, China; 8 Beijing Institute for Stem Cell and Regenerative Medicine, Beijing, China; 9 First Medical Center, Chinese PLA General Hospital, Beijing, China; 10 Precision medicine institute, Changgeng Hospital, Tsinghua University, Beijing, China

**Keywords:** Mesenchymal stem cells, Respiratory tract diseases

Correction to: *Signal Transduction and Targeted Therapy* (2025) **10**:179; 10.1038/s41392-025-02262-3, published online 04 June 2025

In this article^1^ co-authors Meng Li and Huaping Huang should have been denoted as equally contributing authors.

The original article has been corrected.
