# Supplementary material for: Clinical investigation on nebulized human umbilical cord MSC-derived extracellular vesicles for pulmonary fibrosis treatment
Source: Signal Transduct Target Ther. 2025 Jun 4;10:179. doi: 10.1038/s41392-025-02262-3 (PMC12134356; doi:10.1038/s41392-025-02262-3)
Supplement: Supplementary file 1 — Supplementary Materials [file 41392_2025_2262_MOESM1_ESM.docx]

Supplementary Materials for

**Clinical investigation on nebulized human umbilical cord MSC-derived extracellular vesicles for pulmonary fibrosis treatment**

**Meng Li^1, #^, Huaping Huang^2, #^,** **Xiaofei Wei^3^, Huajuan Li^2^, Jun Li^4^, Bingchen Xie^10^, Yuze Yang^1^, Xingyue Fang^8^, Lei Wang^3^, Xiaona Zhang^1^, Heyu Wang^1^, Mengdi Li^1^, Yuting Lin^1^, Dezhi Wang^6^, Yinyin Wang^1^, Tongbiao Zhao^7^, Jianqiu Sheng^6^, Xinbao Hao^1,8^, Muyang Yan^5*^, Lu Xu^1,8*^, Zhijie Chang^1,9*^**

Zhijie Chang E-mail: [zhijiec@mail.tsinghua.edu.cn](mailto:zhijiec@mail.tsinghua.edu.cn).

Lu Xu E-mail: [xulu2324@163.com](mailto:xulu2324@163.com).

Muyang Yan E-mail:yanmy301@sina.com

**This PDF file includes:**

Materials and Methods

Supplementary Text

Supplementary Figures. 1 to 4

Supplementary Tables 1 to 10

**Other Supplementary Materials for this manuscript include the following:**

Supplementary Data 1 to 5

Materials and Methods

**Tandem mass tag (TMT)-based quantitative proteomic analysis**

Bicinchoninic acid (BCA) assay was performed to quantify the protein concentration. Proteins were subject to tandem mass tag (TMT)-based quantitative proteomic analysis by Wayen Biotechnologies, Inc. (Shanghai, China). The abundance of the proteins was presented as a read number according to the analysis from the company (Wayen Biotechnologies, Inc., Shanghai, China). The differentially expressed proteins (DEPs) were regarded as significantly expressed proteins based on fold change >1.20 or <0.80 as well as p < 0.05.

**Small RNA library construction and sequencing**

Small RNA library construction and sequencing were performed according to a stander protocol. In brief, RNAs of hUCMSC-EVs were isolated using a commercially available total EV RNA isolation kit (Qiagen’s exo RNeasy Serum Plasma Kit, China). The RNA quantity was assessed by 260/280 and 260/230 ratios using a Nanodrop (ThermoFisher, USA) and its integrity was verified on a Bioanalyzer 2100 using an Agilent RNA 6000 Nano assay. Small RNA libraries were prepared according to the manufacturer’s protocol using a NEBNext® Multiplex Small RNA Library Prep Set for Illumina (New England Biolabs, USA). The libraries were size-selected using a Pippen Prep (Sage Science, USA) and quality-checked by Bioanalyzer (Agilent, USA) using an Agilent High Sensitivity DNA Kit (ThermoFisher, USA). Library quantification was performed using a Quant-iT™ dsDNA High Sensitivity Assay Kit (ThermoFisher, USA) and sequencing was carried out on an Illumina NextSeq500 using a mid-output V2 kit in the company (OBiO Technology Company, Shanghai, China).

**Metabolomics analysis of hUCMSC-EVs**

The supernatant from hUCMSCs was centrifuged at 110,000 g for 1.5 h for collecting the EVs in the pellet. The hUCMSC-EVs were re-suspended in 50 mL of saline (0.9% NaCl) for purification under a centrifuge at 110,000 g for 1.5 h. The hUCMSC-EVs were resolved in 110 μL of saline and transfer into a 1.5 ml centrifuge tube for storage. All the operation was kept on ice. An amount of hUCMSC-EV solution (10 μl) was used for EV counting and the remaining solution (100 μl) was added with 400 μL of MeOH for extraction and then storage at -80°C. Different batches of extracted hUCMSC-EVs after the storage were subject to a preparation of metabolic solution by vortexing (1 min) and centrifuging (15,000 rpm and 4°C for 15 min). The amount of the metabolic solution (450 μl of supernatant) was transferred into a new 1.5 ml centrifuge tube for vacuum concentration in a freezing spin drier. Another amount of the metabolic solution (50 ul) was used for protein concentration measurement. The metabolic pellet was resolved in a normalized volume according to the EV numbers and then analyzed using Waters ACQUITY UPLC I-Class and Sciex QTrap 6500+ LC-MS for the metabolic substances (Sciex, USA). The data was analyzed with Sciex MultiQuant 3.0.2.

**Modeling murine lung injury and treatment with nebulized hUCMSC-EVs**

To establish the BLM-induced pulmonary fibrosis model, mice were subjected to intratracheal instillation of bleomycin sulfate (BLM, 2 mg/kg, Syno, Cat: NSC125066) dissolved in saline under light anesthesia. The mice were nebulized with either saline (200 μl) or different amounts of hUCMSC-EVs (2.5 × 10^7^, 7.5 × 10^7^, 2.25 × 10^8^ particles) diluted in saline (200 μl) for 2 h after BLM intratracheal instillation. After nebulization inhalation for 14 times during day 1 to 14 and micro-CT assessment on day 18, mice were euthanized at day 21 following the BLM-induced injury. The left lung underwent morphometric analyses, while the right lungs were excised for further examinations.

hUCMSC-EVs were fluorescently labeled with DiR (1,1-dioctadecyl-3,3,3,3-tetramethylindotricarbocyanine iodide), a cell membrane dye (D12731, Invitrogen, USA) for the study on distributions in mice. To prepare hUCMSC-EVs for *in vivo* imaging, the pellet obtained after centrifugation at 100,000 g for 2 h was re-suspended in 5 ml of 10 μM DiR in PBS. After thorough mixing, the hUCMSC-EVs were incubated in the DiR/PBS solution for 15 min at room temperature in the dark, followed by ultracentrifugation at 100,000 g for 1 h. The final pellet was re-suspended in 50 μl of PBS and stored at -80℃.

The nebulization process started from the morning of the experimental day after BLM challenge in mice. Inhalation of hUCMSCs-EVs was administered using a vibrating mesh nebulizer set (YAN30010, Penn Century, USA) with a total volume of 200 μl solution. C57BL/6 mice were administered with hUCMSC-EVs via nebulization at a concentration of 1×10^8^ particles per mouse, and the bio-distribution was continuously monitored for up to 28 days.

**Western blot**

Proteins from hUCMSC-EVs were harvested in RIPA lysis buffer (strong) supplemented with protease inhibitors (Roche, 4693124001, Switzerland). A total of 40 µg proteins were then separated using SDS-PAGE gels and subsequently transferred onto PVDF membranes, which were blocked with milk at room temperature for 1 h and subsequently incubated overnight at 4°C with primary antibodies, including CD9 (Catalogue No. AHS0902), CD81 (Catalogue No. 10630D, Thermo, USA), CD63 (Catalogue No. 10628D, Thermo, USA), and CANX (Catalogue No. A15631, Abclonal, USA). After incubation, the membranes were washed three times with TBST and then incubated for 1 h with an anti-rabbit or anti-mouse IgG antibody.

**Flow cytometry**

Cells were harvested and blocked with 2% bovine serum albumin (BSA; Sigma-Aldrich, B2064) for 20 min at room temperature. Then, the cells were stained with fluorescein-conjugated antibodies for 40 min at room temperature in 1% BSA. After incubation, cells were washed 3 times and analyzed with MoFlo Cytometry (Beckman, USA) and associated software (CytExpert, Beckman, USA). The antibodies used for flow cytometry were as follows: FITC-conjugated anti-mouse CD11b (Cat. 101205), APC-conjugated anti-mouse CD206 (Cat. 141707), PE-conjugated anti-mouse Siglec-F (Cat. 155505) APC-conjugated mouse IgG1 (Cat. 405308), PE-conjugated mouse IgG1 (Cat. 405307), FITC-conjugated mouse IgG1 (Cat. 406001), (all from Biolegend, San Diego, CA, USA). The dilution ratio of the antibody for flow cytometry is 1:40.

**Histology**

Mouse tissues were fixed using a 4% paraformaldehyde solution, and subsequently embedded in paraffin. Sections of 4 μm thickness were de-paraffinized using xylene and then gradually hydrated with alcohol. For Hematoxylin and Eosin (H&E) and Masson staining, sections embedded in paraffin were employed. The H&E-stained sections were utilized for Ashcroft scoring, which was determined by averaging the scores assigned by one blinded and one non-blinded evaluators.

**Real-time quantitative polymerase chain reaction (RT-qPCR)**

Total RNA was extracted with TRIzol (Invitrogen, USA) and reverse-transcribed using the Quantscript RT Kit (TIANGEN Biotech, China). For quantitative polymerase chain reaction (RT-qPCR) analysis, the Talent qPCR PreMix (SYBR Green) Kit (TIANGEN Biotech, China) was utilized on a Roche instrument under the specified condition: initial denaturation at 95°C for 5 s, followed by annealing at 60°C for 10 s, and extension at 72°C for 15 s. The primer sequences employed for the RT-qPCRs are detailed in supplementary Table S10.

**Stem-Loop RT-qPCR**

Total RNA from each lung tissue sample was obtained using MolPure Cell/Tissue miRNA kit (YEASEN, China) according to the manufacturer's recommendations. 1 μg of total RNA was used to synthesize first-strand cDNA with the miRNA 1st Strand cDNA Synthesis Kit (by stem-loop) (Vazyme) for RT-qPCR detection of miRNA -486. Genomic DNA (gDNA) was removed by adding 2 μl of 5 × gDNA Wiper Mix to a total reaction volume of 10 μl, followed by incubation at 42 °C for 2 minutes. First-strand cDNA was synthesized by adding stem-loop primer, 10 × RT mix, and HiScript II Enzyme Mix to a total volume of 20μl, followed by incubation at 25 °C for 5 min, 50 °C for 15 min, and 85 °C for 5 min. Stem-loop RT-qPCR was performed to quantify miRNA-486. The reaction mixture contained 2 × miRNA Universal SYBR qPCR Master Mix, a specific forward primer (Primer F), the universal reverse primer (mQ Primer R), and 2 μl of first-strand cDNA as the template, with H₂O added to a final volume of 20 μl. The qPCR conditions were as follows: initial denaturation at 95 °C for 5 minutes, followed by 40 cycles of 95 °C for 10 seconds and 60 °C for 30 seconds, concluding with a melting curve analysis. The primers were designed using miRNA Primer Design V1.01 software (Vazyme, China), and listed in Supplementary Table S16

**Blinding procedure**

A dedicated research coordinator was tasked with the responsibilities of randomizing the codes confidentially and decoding, preparing nebulization solutions with hUCMSC-EVs or saline, and facilitating information exchange among the team members. An assigned research physician monitored the patient status and recorded pertinent information, and a designated research nurse administered nebulization solutions and conducted safety monitoring. Expertly trained research physicians were entrusted with patient follow-up and additional diagnostic procedures. Importantly, to uphold data objectivity and ensure the credibility of the study, these professionals operated independently, maintaining the confidentiality of the respective findings throughout the study duration.

**Statistics**

The data were presented as mean ± SEM. Survival curves were constructed using the Kaplan-Meier method and evaluated using the generalized Wilcoxon test. Statistical evaluations were conducted using GraphPad Prism 8.0 software (San Diego, CA, USA). For comparisons among multiple groups, Tukey's multiple comparison test in ANOVA was employed. A p-value less than 0.05 was deemed statistically significant. The overall index a Pearson test was conducted. A paired t test was performed for the patient benefits among different indexes.

Supplementary Text

**Inclusion Criteria**

1. Age between 18 and 80 years (inclusive), no gender restriction.
2. Meets HRCT criteria for pulmonary fibrotic lesions:
   1. Manifestations include linear, rigid high-density shadows or nodular high-density shadows in the lungs.
   2. Manifestations include diffuse reticular, linear, honeycomb-like shadows or reticulonodular shadows in both lungs.
3. Typical HRCT imaging features of pulmonary fibrotic lesions within the past 12 weeks, including idiopathic pulmonary fibrosis, COPD with fibrosis, and chronic cough caused by organizing pneumonia post-COVID-19 infection.
4. Able to understand and cooperate with the pulmonary function test procedures.
5. Fully informed about the purpose, methods, and possible discomfort of the trial, agrees to participate, and voluntarily signs the informed consent form.
6. Good compliance, willing to follow the medication regimen as required by the protocol and attend follow-up examinations on time.

**Exclusion Criteria:**

1. Previous stem cell therapy.
2. Intolerance to nebulized inhalation therapy.
3. Allergic constitution or history of potentially life-threatening drug allergies.
4. Pregnant or planning to become pregnant soon, or breastfeeding women.
5. Male participants with reproductive potential and female participants of childbearing age unwilling to use effective contraception during the treatment period and for 12 months following the end of the follow-up.
6. History of malignant tumors or systemic anti-cancer treatment within 5 years prior to the screening period.
7. Active hepatitis B or C virus infection, or HIV infection.
8. History of organ transplantation or currently awaiting organ transplantation.
9. Underwent surgery (excluding diagnostic surgery) within 8 weeks prior to enrollment, planning to undergo surgery during the study period, or with an unhealed surgical wound prior to enrollment.
10. Taking or planning to take nintedanib or pirfenidone within the past month.
11. Any of the following lung diseases: bronchial asthma, active pulmonary tuberculosis, pulmonary embolism, pneumothorax, pneumoconiosis, idiopathic pulmonary arterial hypertension, obliterative bronchiolitis, or other active lung diseases.
12. Current or recent (within 4 weeks) pneumonia.
13. Previous lung resection surgery.
14. Currently requiring oxygen therapy for more than 15 hours per day.
15. History of mental illness, epilepsy, or other central nervous system diseases.
16. Severe other systemic diseases such as myocardial infarction, unstable angina, heart failure, liver cirrhosis, acute glomerulonephritis, etc.
17. Participation in any other clinical trial within 3 months prior to screening.
18. Currently participating in another clinical trial.
19. Poor compliance, making it difficult to complete the study.
20. Any condition that the researcher believes may increase the risk to the participant or interfere with the study results.

Treatment Discontinuation Criteria:

Regardless of the reason for discontinuation, any patient should consult the study physician before deciding to discontinue treatment.

1. Subject Termination Criteria

(1) The subject withdraws informed consent and requests to withdraw.

(2) The subject exhibits severe non-compliance.

(3) Pregnancy, serious adverse events, death, or loss to follow-up occur.

(4) Use of prohibited concomitant medication.

(5) The subject does not return for visits.

Researchers should make every effort to ensure that each subject can continue follow-up observations unless discontinuing participation is in the best interest of the subject. After withdrawing from the study, subjects should enter the follow-up period and will undergo periodic follow-ups to determine survival status until death or the end of the study.

1. Investigator-Initiated Early Termination

The clinical study will be terminated if any of the following issues are discovered:

(1) Violation of inclusion criteria or meeting exclusion criteria.

(2) Due to adverse events, the investigator determines the trial should not continue.

(3) Serious safety issues occur during the study (e.g., serious adverse reactions, severe complications, or rapid deterioration of the subject's condition).

(4) The study is found to be ineffective or have poor outcomes, lacking clinical value, and continuing would delay treatment for the subjects.

(5) Major errors or significant deviations in the clinical study protocol are found during the study, making it difficult to evaluate if continued.

(6) Other reasons deemed necessary by the investigator to discontinue medication.

Supplementary Fig.1


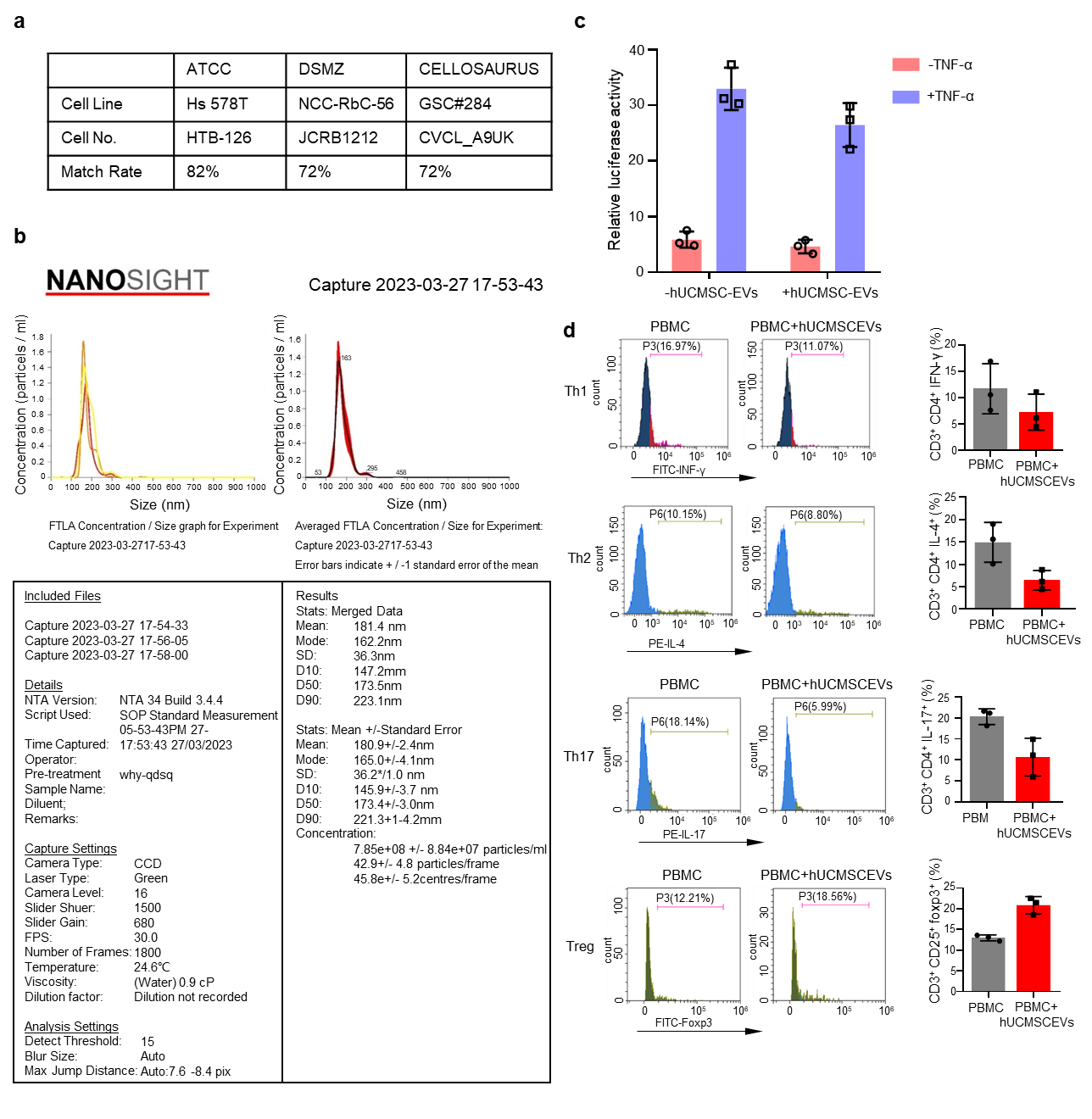


Supplementary Fig.1. Quality administration at each critical control point

**a** Cell STR analysis by Short Tandem Repeats alignment. **b** Representative particle analysis by NTA. **c** Luciferase equal volumes from aliquots were evaluated for TNF-α decoy in a reporter cell assay responsive to TNF-α induced NF-κB activation. Data were normalized to cells treated with TNF-α only. **d** FACS analysis of EVs co-cultured with T cells reveals the proportions of different subsets of Th1, Th2, Th17, and Treg.

Supplementary Fig.2


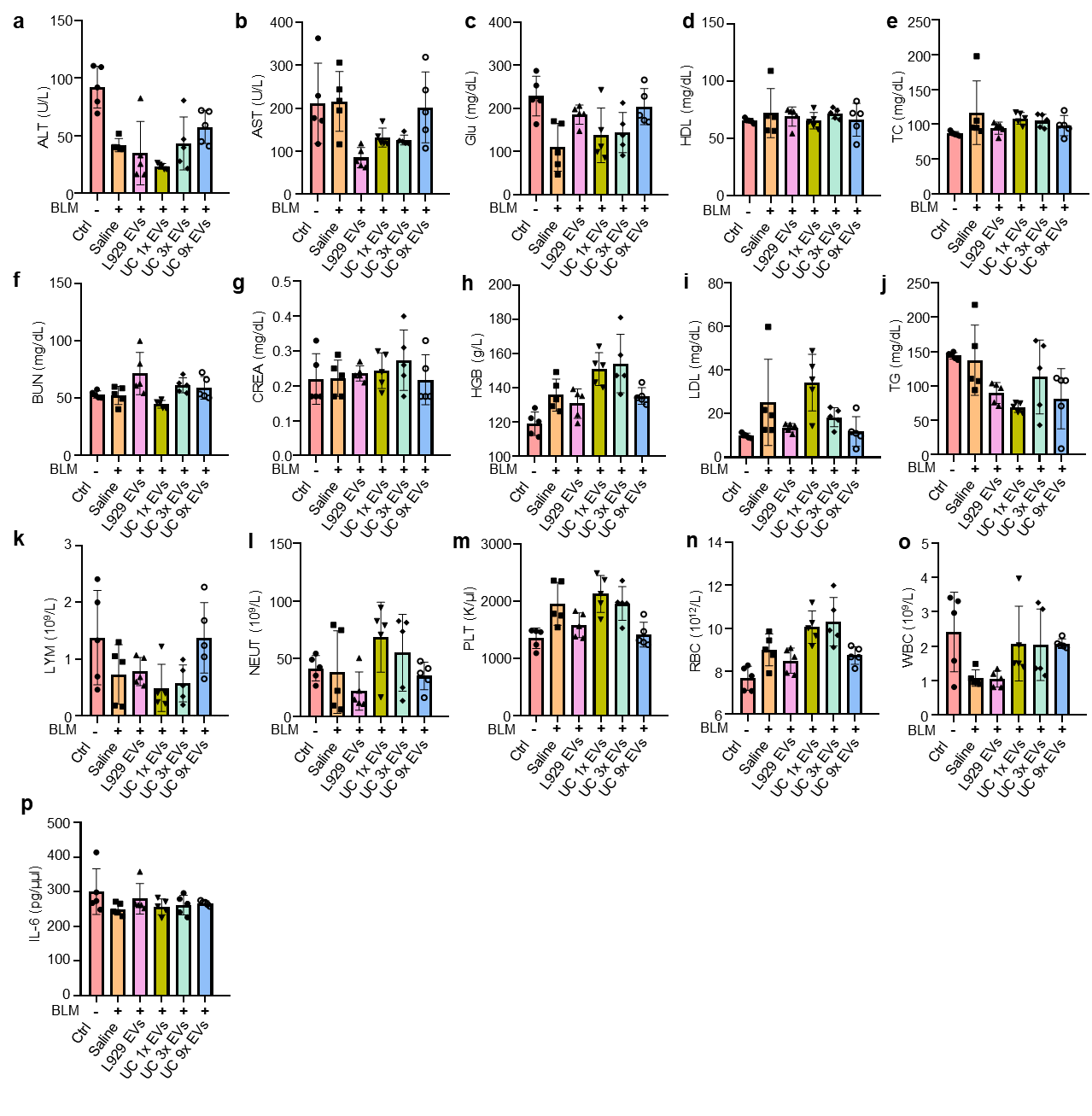


Supplementary Fig.2. Safety of nebulized hUCMSC-EVs in treating injury-induced lung fibrosis in mice.

Laboratory indicators of ALT (**a**), AST (**b**), Glu (**c**), HDL (**d**), TC (**e**), BUN (**f**), CREA (**g**), HGB (**h**), LDL (**i**), TG (**j**), LYM (**k**), NEUT (**l**), PLT (**m**), RBC (**n**), WBC (**o**), and IL-6 (**p**) before and after hUCMSC-EVs nebulization in mice.

ALT: Alanine aminotransferase. AST: Aspartate aminotransferase. Glu: Glucose. HDL: High-density lipoprotein. TC: Total cholesterol. BUN: Blood urea nitrogen. CREA: Creatinine. HGB: Hemoglobin. LDL: Low-density lipoprotein. TG: Triglycerides. LYM: Lymphocyte. NEUT: Neutrophil. PLT: Platelet. RBC: Red blood cell. WBC: White blood cell. IL-6: Interleukin 6.

Supplementary Fig.3.


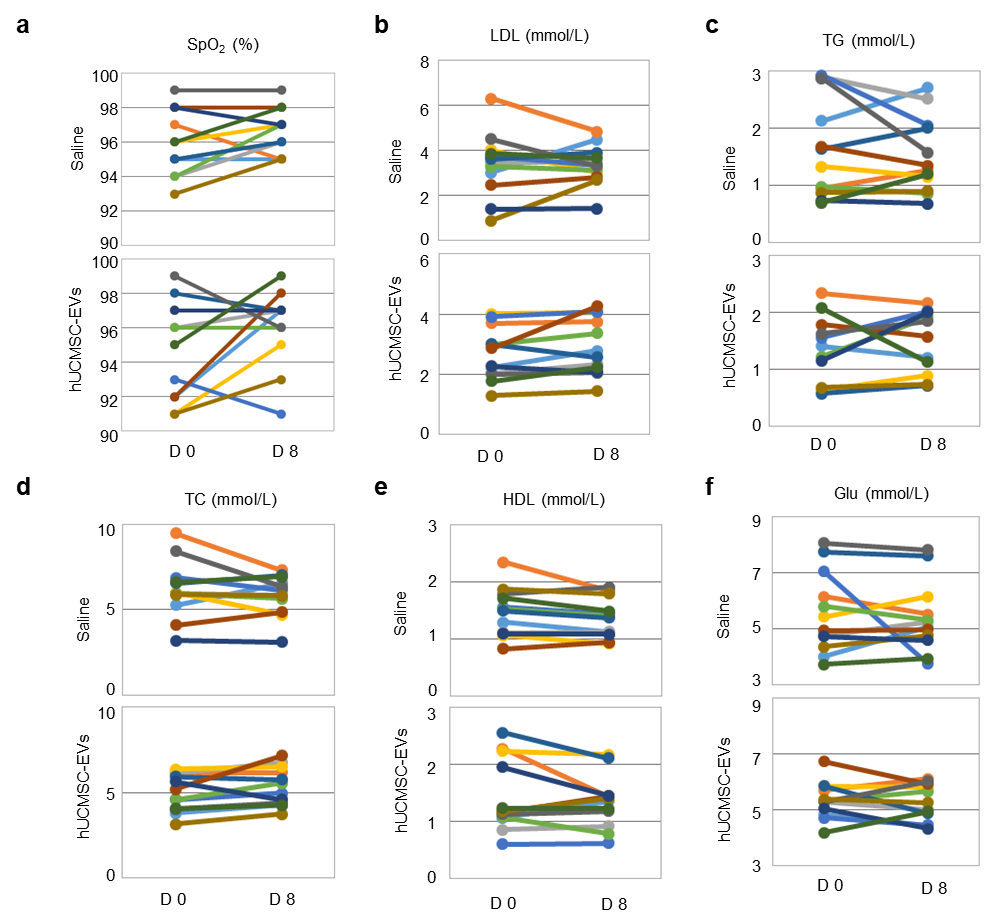


Supplementary Fig.3. Clinical safety of nebulized hUCMSC-EVs on pulmonary fibrosis

Laboratory indicators including SpO_2_ (**a**), LDL (**b**), TG (**c**), TC (**d**), HDL (**e**), Glu (**f**) before and after hUCMSC-EVs nebulization in the clinical trial.

SpO_2_: Saturation of Peripheral Oxygen. LDL: Low-density lipoprotein. TG: Triglyceride. TC: Serum total cholesterol. HDL: high-density lipoprotein. Glu: Glucose.

Supplementary Fig.4


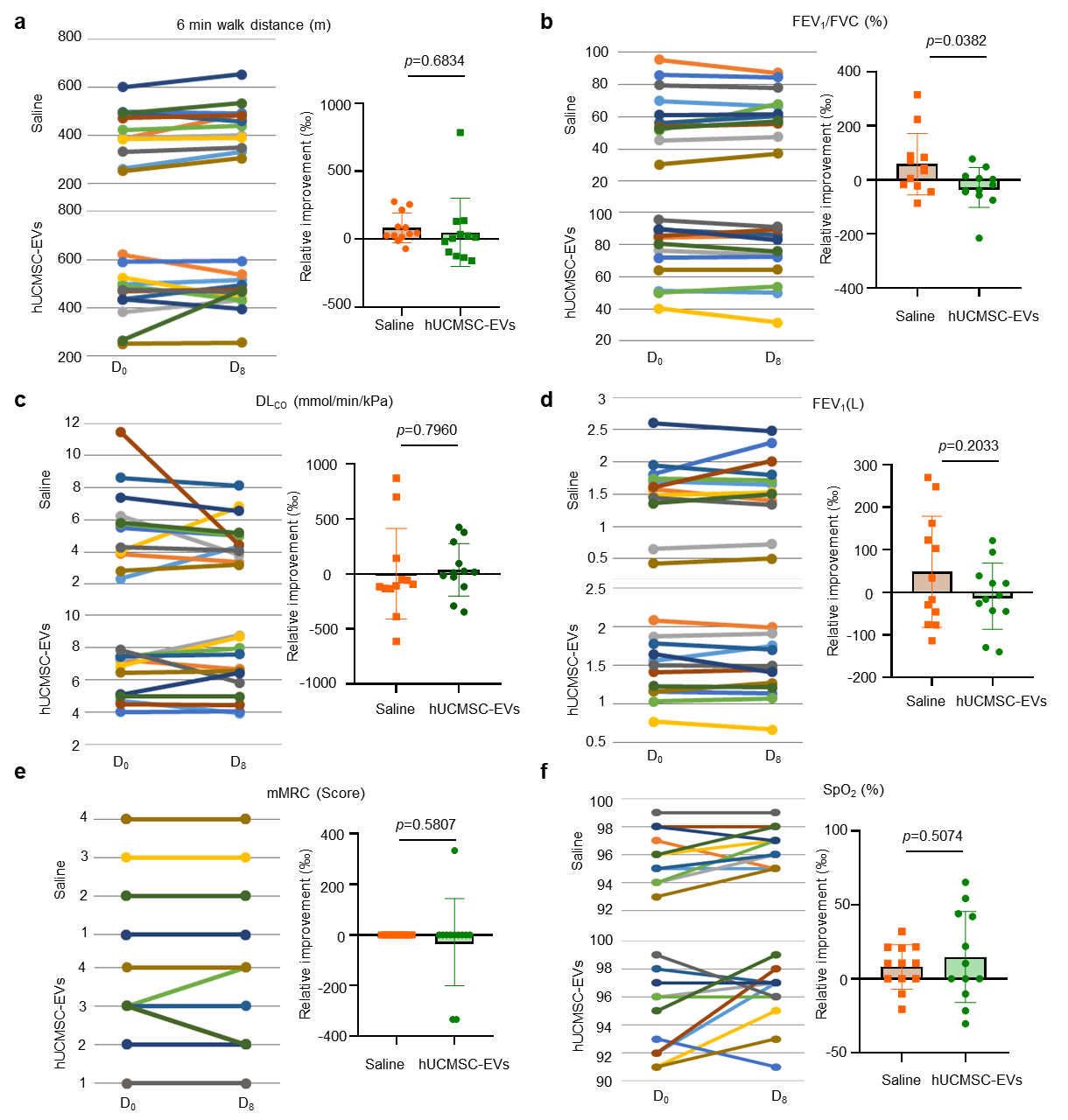


Supplementary Fig.4. Clinical efficacy of nebulized hUCMSC-EVs on pulmonary fibrosis

Levels and statistical analysis of 6 min walk distance (**a**), FEV_1_/FVC (**b**), DL_CO_ (**c**), and FEV_1_(**d**), mMRC (**e**), and SpO_2_ (**f**) before and after saline and hUCMSC-EVs nebulization in the clinical trial.

FEV_1_: Forced Expiratory Volume in One Second. FVC: Forced Vital Capacity. DL_CO_: Diffusing Capacity of the Lung for Carbon Monoxide. mMRC: modified Medical Research Council. SpO_2_: Saturation of Peripheral Oxygen..

Supplementary Table 1.

Table S1. Comparison of baseline characteristics

|  | Experimental group  (n=12) | Control group  (n=12) | *P*-value |
| --- | --- | --- | --- |
| Age(years),median+IQR | 65.50[59.50-69.00] | 64.5[56.75-66.00] | 0.372 |
| Female, n(%) | 6(50) | 2(16.7) | 0.424 |
| FVC%predicted, median+IQR | 73.50  (47.24-120.22) | 80.10 (41.00-112.21) | 0.493 |
| MVV%predicted, median+IQR | 51.10  (20.94-70.95) | 55.10 (15.42-87.33) | 0.620 |
| DLco%predicted, median+IQR | 57.50  (24.80-82.60) | 70.80 (16.40-145.90) | 0.255 |
| 6MWD(m),  median+IQR | 452.80  (498.00-621.00) | 415.50 (261.00-600.00) | 0.407 |
| mMRC, median+IQR | 1[1-2] | 1[1-3] | 0.505 |

FVC= forced vital capacity; MVV=Maximal Voluntary Ventilation; DL_CO_=diffusing capacity for carbon monoxide; 6MWD= 6-min walk distance; mMRC=modified Medical Research Council; IQR= interquartile range.

Supplementary Table 2. Baseline treatments of Participants in the study

| Experimental group | Treatment received | Oxygen support | Control group | Treatment received | Oxygen support |
| --- | --- | --- | --- | --- | --- |
| 001 | Ultibro | None | 004 | Warfarin | None |
| 002 | None | None | 006 | None | None |
| 003 | None | None | 010 | Budesonide | None |
| 005 | Budesonide | None | 012 | Ultibro | None |
| 007 | None | None | 014 | Methylprednisolone+ Cyclophosphamide | None |
| 008 | Budesonide | None | 017 | Ultibro | None |
| 009 | None | None | 018 | None | None |
| 011 | None | None | 019 | Ultibro | None |
| 013 | Methylprednisolone+ Cyclophosphamide | None | 020 | Prednisone+ Cyclophosphamide | None |
| 015 | Budesonide | None | 022 | Budesonide | None |
| 016 | None | None | 023 | Ultibro | None |
| 021 | None | None | 024 | Ultibro | None |

Supplementary Table 3. Schedule of Assessments

| Visit | Screening  -2d～0d | Baseline  D0 | D1 | D2 | D3 | D4 | D5 | D6 | D7 | D8 | D28  ±3d | D84  ±3d | D180  ±5d | D360  ±7d |
| --- | --- | --- | --- | --- | --- | --- | --- | --- | --- | --- | --- | --- | --- | --- |
| **Basic medical history** | | |  |  |  |  |  |  |  |  |  |  |  |  |
| informed consent | × |  |  |  |  |  |  |  |  |  |  |  |  |  |
| Demographic data | × |  |  |  |  |  |  |  |  |  |  |  |  |  |
| General clinical data | × |  |  |  |  |  |  |  |  |  | × | × |  |  |
| **Security assessment** | | |  |  |  |  |  |  |  |  |  |  |  |  |
| Vital sign | × | × | × | × | × | × | × | × | × | × | × | × |  |  |
| Physical examination | × | × | × | × | × | × | × | × | × | × | × | × |  |  |
| Blood routine examination |  | × |  |  |  |  |  |  |  | × |  | × |  |  |
| urinalysis |  | × |  |  |  |  |  |  |  | × |  | × |  |  |
| Urine pregnancy test (women of gestational age only) |  | × |  |  |  |  |  |  |  | × |  | × |  |  |
| Infectious disease screening |  | × |  |  |  |  |  |  |  |  |  | × |  |  |
| Blood biochemical examination |  | × |  |  |  |  |  |  |  | × |  | × |  |  |
| Tumor marker |  | × |  |  |  |  |  |  |  |  |  | × |  |  |
| IL-6 |  | × |  |  |  |  |  |  |  | × |  | × |  |  |
| Peripheral blood lymphocyte subsets |  | × |  |  |  |  |  |  |  | × |  | × |  |  |
| ECG |  | × |  |  |  |  |  |  |  | × |  | × |  |  |
| Finger oxygen saturation |  | × | × | × | × | × | × | × | × | × | × | × |  |  |
| **Effectiveness evaluation** | | |  |  |  |  |  |  |  |  |  |  |  |  |
| Chest HRCT |  | × |  |  |  |  |  |  |  |  | × | × |  |  |
| Lung function test |  | × |  |  |  | × |  |  |  | × | × | × |  |  |
| Lung diffusion function test |  | × |  |  |  | × |  |  |  | × | × | × |  |  |
| 6MWD |  | × |  |  |  |  |  |  |  | × | × | × |  |  |
| mMRC |  | × |  |  |  |  |  |  |  | × | × | × |  |  |
| SGRQ |  | × |  |  |  |  |  |  |  | × | × | × |  |  |
| LCQ |  | × |  |  |  |  |  |  |  | × | × | × |  |  |
| Acute aggravating event |  |  | × | × | × | × | × | × | × | × | × | × | × | × |
| AE/SAE |  |  | × | × | × | × | × | × | × | × | × | × | × | × |

IL-6=interleukin 6; ECG=electrocardiogram; HRCT=High Resolution Chest Tomography; 6MWD=Six - Minute Walk Distance；SGRQ=St. George's Respiratory Questionnaire；LCQ=**Cough-specific Quality of Life Questionnaire；**mMRC=Modified Medical Research Council；AE/SAE=**Adverse Event/Serious Adverse Event**

Supplementary Table 4. Changes in blood oxygen saturation before and after EVs

| EVs | D0 (SpO_2_%) | D8 (SpO_2_%) | Saline | D0 (SpO_2_%) | D8 (SpO_2_%) |
| --- | --- | --- | --- | --- | --- |
| 001 | 92 | 97 | 004 | 95 | 95 |
| 002 | 97 | 97 | 006 | 97 | 95 |
| 003 | 96 | 97 | 010 | 94 | 96 |
| 005 | 91 | 95 | 012 | 96 | 97 |
| 007 | 93 | 91 | 014 | 95 | 96 |
| 008 | 96 | 96 | 017 | 94 | 97 |
| 009 | 98 | 97 | 018 | 95 | 96 |
| 011 | 92 | 98 | 019 | 98 | 98 |
| 013 | 99 | 96 | 020 | 99 | 99 |
| 015 | 91 | 93 | 022 | 93 | 95 |
| 016 | 97 | 97 | 023 | 98 | 97 |
| 021 | 95 | 99 | 024 | 96 | 98 |

Supplementary Table 5. Changes in laboratory indicators before and after hUCMSC-EVs treatment

|  | 001 | | 002 | | 003 | | 004 | | 005 | | 006 | |
| --- | --- | --- | --- | --- | --- | --- | --- | --- | --- | --- | --- | --- |
|  | Day0 | Day8 | Day0 | Day8 | Day0 | Day8 | Day0 | Day8 | Day0 | Day8 | Day0 | Day8 |
| WBC (×10^9^ /L) | 7.84 | 7.76 | 9.41 | 8.94 | 6.58 | 8.58 | 10.93 | 9.31 | 6.41 | 5.21 | 8.73 | 9.51 |
| N  (×10^9^ /L) | 4.54 | 4.92 | 4.30 | 3.27 | 2.78 | 4.24 | 7.69 | 6.56 | 3.54 | 2.76 | 3.98 | 3.98 |
| LYM (×10^9^ /L) | 2.31 | 1.90 | 3.19 | 3.67 | 2.88 | 3.08 | 1.84 | 1.47 | 1.99 | 1.59 | 3.64 | 4.35 |
| RBC (×10^12^/L) | 5.35 | 5.52 | 4.06 | 3.97 | 4.20 | 4.05 | 4.42 | 4.38 | 4.44 | 4.41 | 4.45 | 4.41 |
| Hb  (g/L) | 156 | 162 | 136 | 132 | 123 | 121 | 132 | 132 | 140 | 140 | 134 | 133 |
| PLT (×10^9^ /L) | 221 | 247 | 235 | 234 | 290 | 281 | 166 | 149 | 179 | 178 | 209 | 196 |
| ALT (U/L) | 67 | 28 | 18 | 15 | 25 | 21 | 31 | 42 | 23 | 13 | 32 | 47 |
| AST (U/L) | 101 | 27 | 21 | 26 | 22 | 21 | 19 | 22 | 30 | 23 | 42 | 45 |
| ALP (U/L) | 82 | 74 | 70 | 79 | 65 | 69 | 38 | 34 | 63 | 57 | 44 | 43 |
| BUN (mmol/L) | 7.34 | 7.94 | 5.94 | 6.38 | 6.46 | 11.26 | 5.95 | 4.40 | 7.24 | 5.88 | 4.13 | 4.85 |
| Cr (μmol/L) | 106.8 | 117.0 | 97.9 | 98.8 | 52.3 | 53.4 | 68.5 | 70.6 | 81.1 | 81.3 | 66.3 | 64.5 |
| GLU (mmol/L) | 4.84 | 4.46 | 5.71 | 6.12 | 5.30 | 5.03 | 3.99 | 5.05 | 5.86 | 5.81 | 6.13 | 5.50 |
| TG (mmol/L) | 1.41 | 1.21 | 2.34 | 2.17 | 8.57 | 9.46 | 2.12 | 2.70 | 0.63 | 0.90 | 0.95 | 1.26 |
| TC (mmol/L) | 3.80 | 4.28 | 6.14 | 6.17 | 6.17 | 6.77 | 5.25 | 6.55 | 6.39 | 6.53 | 9.46 | 7.29 |
| HDL (mmol/L) | 1.07 | 1.36 | 2.28 | 1.45 | 0.86 | 0.92 | 1.29 | 1.11 | 2.24 | 2.18 | 2.34 | 1.84 |
| LDL (mmol/L) | 2.21 | 2.78 | 3.70 | 3.74 | 2.01 | 2.33 | 3.01 | 4.48 | 4.01 | 4.06 | 6.29 | 4.83 |

|  | 007 | | 008 | | 009 | | 010 | | 011 | | 012 | |
| --- | --- | --- | --- | --- | --- | --- | --- | --- | --- | --- | --- | --- |
|  | Day0 | Day8 | Day0 | Day8 | Day0 | Day8 | Day0 | Day8 | Day0 | Day8 | Day0 | Day8 |
| WBC (×10^9^ /L) | 9.03 | 9.06 | 3.1 | 3.13 | 7.43 | 7.16 | 7.42 | 8.62 | 6.27 | 6.28 | 8.48 | 8.10 |
| N  (×10^9^ /L) | 2.80 | 2.70 | 1.48 | 1.27 | 5.62 | 4.60 | 4.50 | 4.60 | 3.39 | 3.26 | 4.99 | 5.01 |
| LYM (×10^9^ /L) | 5.13 | 5.32 | 1.17 | 1.39 | 1.30 | 1.80 | 1.92 | 2.13 | 2.33 | 2.50 | 2.59 | 2.24 |
| RBC (×10^12^/L) | 4.33 | 4.45 | 4.50 | 4.59 | 5.57 | 5.53 | 5.00 | 4.70 | 3.62 | 3.69 | 4.68 | 4.48 |
| Hb  (g/L) | 147 | 150 | 124 | 129 | 152 | 152 | 146 | 140 | 126 | 128 | 152 | 146 |
| PLT (×10^9^ /L) | 143 | 152 | 141 | 158 | 187 | 213 | 233 | 251 | 136 | 129 | 313 | 280 |
| ALT (U/L) | 31 | 32 | 12 | 11 | 24 | 28 | 26 | 24 | 25 | 20 | 15 | 17 |
| AST (U/L) | 68 | 78 | 13 | 12 | 19 | 17 | 21 | 15 | 34 | 28 | 21 | 33 |
| ALP (U/L) | 189 | 192 | 66 | 70 | 96 | 94 | 65 | 63 | 118 | 119 | 62 | 64 |
| BUN (mmol/L) | 6.88 | 7.74 | 4.90 | 4.68 | 4.98 | 4.73 | 3.50 | 4.55 | 5.33 | 4.46 | 5.52 | 4.68 |
| Cr (μmol/L) | 47.2 | 64.1 | 43.1 | 47.8 | 79.5 | 96.1 | 65.7 | 59.8 | 61.8 | 63.0 | 95.4 | 100.2 |
| GLU (mmol/L) | 4.73 | 4.46 | 5.32 | 5.67 | 5.87 | 4.89 | 4.75 | 5.25 | 6.74 | 5.93 | 5.41 | 6.14 |
| TG (mmol/L) | 1.56 | 2.03 | 1.23 | 1.92 | 0.58 | 0.72 | 2.88 | 2.51 | 1.79 | 1.58 | 1.32 | 1.15 |
| TC (mmol/L) | 4.59 | 4.98 | 4.61 | 5.56 | 5.93 | 5.78 | 5.95 | 5.80 | 5.20 | 7.18 | 5.99 | 4.69 |
| HDL (mmol/L) | 0.61 | 0.63 | 1.08 | 0.79 | 2.56 | 2.12 | 1.10 | 1.12 | 1.17 | 1.45 | 1.06 | 0.91 |
| LDL (mmol/L) | 3.91 | 4.08 | 2.99 | 3.36 | 2.99 | 2.57 | 3.45 | 3.45 | 2.86 | 4.26 | 4.00 | 3.01 |

|  | 013 | | 014 | | 015 | | 016 | | 017 | | 018 | |
| --- | --- | --- | --- | --- | --- | --- | --- | --- | --- | --- | --- | --- |
|  | Day0 | Day8 | Day0 | Day8 | Day0 | Day8 | Day0 | Day8 | Day0 | Day8 | Day0 | Day8 |
| WBC (×10^9^ /L) | 12.64 | 11.79 | 9.69 | 10.67 | 5.93 | 4.21 | 5.18 | 5.56 | 5.44 | 6.46 | 5.55 | 5.98 |
| N  (×10^9^ /L) | 8.69 | 7.87 | 8.00 | 6.71 | 3.70 | 2.02 | 3.21 | 3.09 | 3.51 | 4.08 | 2.61 | 2.72 |
| LYM (×10^9^ /L) | 2.77 | 2.89 | 1.33 | 2.85 | 1.18 | 1.45 | 1.51 | 1.96 | 1.43 | 1.83 | 2.24 | 2.42 |
| RBC (×10^12^/L) | 4.21 | 4.29 | 4.69 | 4.80 | 3.42 | 3.82 | 4.36 | 4.34 | 4.99 | 4.87 | 6.28 | 6.02 |
| Hb  (g/L) | 130 | 128 | 139 | 144 | 115 | 130 | 118 | 118 | 147 | 142 | 162 | 156 |
| PLT (×10^9^ /L) | 301 | 277 | 368 | 380 | 130 | 104 | 233 | 229 | 268 | 282 | 171 | 187 |
| ALT (U/L) | 13 | 9 | 24 | 27 | 39 | 19 | 47 | 21 | 8 | 7 | 15 | 16 |
| AST (U/L) | 13 | 14 | 20 | 22 | 15 | 19 | 33 | 21 | 25 | 19 | 18 | 18 |
| ALP (U/L) | 47 | 49 | 54 | 63 | 55 | 64 | 77 | 68 | 64 | 55 | 52 | 46 |
| BUN (mmol/L) | 4.53 | 4.64 | 3.55 | 3.74 | 1.40 | 2.01 | 4.31 | 2.32 | 6.82 | 7.91 | 6.98 | 7.38 |
| Cr (μmol/L) | 49.6 | 52.6 | 61.6 | 60.4 | 57.0 | 57.2 | 50.6 | 56.9 | 62.4 | 66.0 | 85.0 | 83.7 |
| GLU (mmol/L) | 5.31 | 6.04 | 7.04 | 3.74 | 5.39 | 5.27 | 5.05 | 4.33 | 5.78 | 5.29 | 7.73 | 7.60 |
| TG (mmol/L) | 1.63 | 1.85 | 2.92 | 2.04 | 0.69 | 0.74 | 1.16 | 2.01 | 0.97 | 0.85 | 1.63 | 2.00 |
| TC (mmol/L) | 4.05 | 4.39 | 6.86 | 6.13 | 3.16 | 3.74 | 5.66 | 4.61 | 5.92 | 5.64 | 6.54 | 7.02 |
| HDL (mmol/L) | 1.13 | 1.19 | 1.55 | 1.44 | 1.19 | 1.41 | 1.96 | 1.46 | 1.52 | 1.40 | 1.49 | 1.37 |
| LDL (mmol/L) | 2.00 | 2.04 | 3.71 | 3.33 | 1.29 | 1.44 | 2.26 | 2.06 | 3.28 | 3.12 | 3.61 | 3.89 |

|  | 019 | | 020 | | 021 | | 022 | | 023 | | 024 | |
| --- | --- | --- | --- | --- | --- | --- | --- | --- | --- | --- | --- | --- |
|  | Day0 | Day8 | Day0 | Day8 | Day0 | Day8 | Day0 | Day8 | Day0 | Day8 | Day0 | Day8 |
| WBC (×10^9^ /L) | 6.52 | 5.69 | 11.17 | 7.75 | 8.96 | 10.98 | 5.41 | 4.32 | 7.13 | 6.56 | 7.02 | 7.75 |
| N  (×10^9^ /L) | 3.61 | 3.19 | 9.11 | 6.55 | 5.22 | 7.00 | 4.14 | 3.01 | 4.88 | 4.36 | 4.65 | 4.81 |
| LYM (×10^9^ /L) | 2.33 | 1.84 | 1.34 | 0.64 | 2.74 | 2.85 | 0.89 | 0.90 | 1.64 | 1.55 | 1.59 | 1.90 |
| RBC (×10^12^/L) | 4.54 | 4.42 | 4.12 | 3.65 | 4.07 | 4.04 | 5.59 | 5.80 | 5.44 | 5.32 | 4.78 | 4.81 |
| Hb  (g/L) | 128 | 124 | 127 | 115 | 120 | 122 | 154 | 163 | 160 | 154 | 143 | 145 |
| PLT (×10^9^ /L) | 315 | 288 | 399 | 275 | 280 | 297 | 177 | 199 | 259 | 262 | 271 | 369 |
| ALT (U/L) | 51 | 33 | 21 | 23 | 28 | 18 | 16 | 16 | 26 | 34 | 22 | 28 |
| AST (U/L) | 45 | 26 | 18 | 21 | 29 | 21 | 19 | 18 | 26 | 23 | 22 | 29 |
| ALP (U/L) | 77 | 75 | 74 | 71 | 55 | 62 | 79 | 95 | 84 | 83 | 95 | 105 |
| BUN (mmol/L) | 4.37 | 4.42 | 8.18 | 6.72 | 4.01 | 4.46 | 5.62 | 4.21 | 4.18 | 3.40 | 5.57 | 4.25 |
| Cr (μmol/L) | 57.1 | 52.8 | 117.8 | 111.3 | 44.4 | 50.7 | 75.4 | 86.2 | 66.4 | 63.2 | 57.6 | 63.6 |
| GLU (mmol/L) | 4.93 | 4.97 | 8.05 | 7.81 | 4.20 | 4.96 | 4.34 | 4.75 | 4.72 | 4.58 | 3.72 | 3.93 |
| TG (mmol/L) | 1.67 | 1.34 | 2.87 | 1.57 | 2.08 | 1.13 | 0.87 | 0.89 | 0.73 | 0.67 | 0.69 | 1.19 |
| TC (mmol/L) | 4.10 | 4.86 | 8.41 | 6.33 | 4.06 | 4.28 | 5.87 | 5.80 | 3.17 | 3.10 | 6.60 | 6.91 |
| HDL (mmol/L) | 0.82 | 0.94 | 1.78 | 1.90 | 1.24 | 1.23 | 1.87 | 1.79 | 1.09 | 1.08 | 1.71 | 1.48 |
| LDL (mmol/L) | 2.46 | 2.81 | 4.49 | 3.29 | 1.76 | 2.23 | 0.87 | 2.67 | 1.38 | 1.41 | 3.84 | 3.66 |

Reference range: WBC (×10^9^ /L)：4.00～10.00；N (×109 /L)：2.00～7.00；LYM (×10^9^ /L)：1.32～3.57；RBC (×10^12^/L)：4.00～5.50；Hb (g/L)：120～160；PLT (×10^9^ /L)：100～300；ALT (U/L)：9～50；AST (U/L)：15～40；ALP (U/L)：45～125；BUN (mmol/L)：3.1～8.0；Cr (μmol/L)：57～97；GLU (mmol/L)：3.90～8.00；TG (mmol/L)：﹤1.70；TC (mmol/L)：﹤5.18；HDL (mmol/L)：1.00～1.60；LDL (mmol/L)：≤3.30.

Conclusion: No significant changes were observed in the complete blood count, liver and kidney function, blood glucose, and blood lipids before and after exosomal treatment.

Supplementary Table 6. Changes of IL-6 before and after EVs

| EVs | D 0（IL-6） | D 8（IL-6） | Saline | D 0（IL-6） | D 8（IL-6） |
| --- | --- | --- | --- | --- | --- |
| 001 | <1.5 | <1.5 | 004 | 3.85 | 25.1 |
| 002 | <1.5 | 3.9 | 006 | 5.44 | <1.5 |
| 003 | 303.31 | 281.94 | 010 | 3.06 | 1.52 |
| 005 | <1.5 | <1.5 | 012 | <1.5 | <1.5 |
| 007 | 28.32 | 16.94 | 014 | <1.5 | 5.6 |
| 008 | 2.99 | <1.5 | 017 | <1.5 | <1.5 |
| 009 | <1.5 | <1.5 | 018 | <1.5 | <1.5 |
| 011 | 5.88 | 4.03 | 019 | <1.5 | <1.5 |
| 013 | 17.57 | 5.27 | 020 | <1.5 | <1.5 |
| 015 | <1.5 | <1.5 | 022 | <1.5 | <1.5 |
| 016 | <1.5 | <1.5 | 023 | <1.5 | <1.5 |
| 021 | <1.5 | 11.74 | 024 | <1.5 | <1.5 |

Reference interval：IL-6<7pg/ml

Supplementary Table 7. Data table of changes in pulmonary function before and after hUCMSC-EVs treatment.

| EVs | Day | FVC  （L） | FEV_1_  （L） | FEV_1_/FVC  (%) | MVV  （L/min） | DL_CO_  _（mmol/min/kPa）_ | Saline | Day | FVC  （L） | FEV_1_  （L） | FEV_1_/FVC  (%) | MVV  （L/min） | DL_CO_  _（mmol/min/kPa）_ |
| --- | --- | --- | --- | --- | --- | --- | --- | --- | --- | --- | --- | --- | --- |
| 001 | D 0 | 3.06 | 1.56 | 51.08 | 63.63 | 2.71 | 004 | D 0 | 2.43 | 1.70 | 69.74 | 55.14 | 2.34 |
|  | D 8 | 3.49 | 1.75 | 50.15 | 55.35 | 1.92 |  | D 8 | 2.47 | 1.65 | 66.66 | 62.42 | 4.38 |
| 002 | D 0 | 2.46 | 2.08 | 84.29 | 70.95 | 5.27 | 006 | D 0 | 1.65 | 1.58 | 95.49 | 83.82 | 3.88 |
|  | D 8 | 2.33 | 1.99 | 85.43 | 99.17 | 4.66 |  | D 8 | 1.60 | 1.40 | 87.25 | 63.59 | 3.36 |
| 003 | D 0 | 2.44 | 1.87 | 76.32 | 36.11 | 5.23 | 010 | D 0 | 1.42 | 0.65 | 45.51 | 22.60 | 6.24 |
|  | D 8 | 2.60 | 1.91 | 73.34 | 46.00 | 6.76 |  | D 8 | 1.54 | 0.73 | 47.51 | 24.65 | 3.81 |
| 005 | D 0 | 1.90 | 0.77 | 40.30 | 24.15 | 4.85 | 012 | D 0 | 2.62 | 1.48 | 56.29 | 43.82 | 4.01 |
|  | D 8 | 2.12 | 0.67 | 31.62 | 25.43 | 6.69 |  | D 8 | 2.50 | 1.53 | 60.99 | 50.88 | 6.82 |
| 007 | D 0 | 1.61 | 1.16 | 71.79 | 57.94 | 2.00 | 014 | D 0 | 2.10 | 1.81 | 85.96 | 48.38 | 5.52 |
|  | D 8 | 1.56 | 1.13 | 72.19 | 59.87 | 2.02 |  | D 8 | 2.73 | 2.30 | 84.44 | 42.76 | 5.00 |
| 008 | D 0 | 2.06 | 1.03 | 50.00 | 45.00 | 5.46 | 017 | D 0 | 3.37 | 1.74 | 51.70 | 43.21 | 5.77 |
|  | D 8 | 1.98 | 1.07 | 53.89 | 42.56 | 5.98 |  | D 8 | 2.52 | 1.71 | 67.97 | 53.83 | 5.01 |
| 009 | D 0 | 2.00 | 1.78 | 89.25 | 49.80 | 5.45 | 018 | D 0 | 3.46 | 1.95 | 56.32 | 82.79 | 8.62 |
|  | D 8 | 1.98 | 1.70 | 85.91 | 54.62 | 5.59 |  | D 8 | 2.98 | 1.80 | 60.36 | 83.92 | 8.13 |
| 011 | D 0 | 1.65 | 1.41 | 85.35 | 55.51 | 2.50 | 019 | D 0 | 2.99 | 1.61 | 53.82 | 51.79 | 11.46 |
|  | D 8 | 1.61 | 1.44 | 89.47 | 58.13 | 2.43 |  | D 8 | 3.61 | 2.01 | 55.66 | 47.36 | 4.42 |
| 013 | D 0 | 1.57 | 1.50 | 95.32 | 55.47 | 5.86 | 020 | D 0 | 1.82 | 1.45 | 79.66 | 58.56 | 4.30 |
|  | D 8 | 1.64 | 1.49 | 91.11 | 36.05 | 3.83 |  | D 8 | 1.72 | 1.34 | 77.90 | 54.64 | 4.08 |
| 015 | D 0 | 1.80 | 1.16 | 64.29 | 37.58 | 4.46 | 022 | D 0 | 1.42 | 0.43 | 30.49 | 16.17 | 2.82 |
|  | D 8 | 1.97 | 1.27 | 64.46 | 47.20 | 4.54 |  | D 8 | 1.34 | 0.50 | 37.31 | 16.80 | 3.22 |
| 016 | D 0 | 1.83 | 1.64 | 89.71 | 30.61 | 3.10 | 023 | D 0 | 4.24 | 2.60 | 61.28 | 101.79 | 7.39 |
|  | D 8 | 1.70 | 1.41 | 83.00 | 39.97 | 4.42 |  | D 8 | 4.03 | 2.48 | 61.60 | 106.64 | 6.54 |
| 021 | D 0 | 1.53 | 1.23 | 80.55 | 39.99 | 2.99 | 024 | D 0 | 2.60 | 1.36 | 52.65 | 47.05 | 5.82 |
|  | D 8 | 1.59 | 1.21 | 75.96 | 41.98 | 2.95 |  | D 8 | 2.62 | 1.50 | 57.36 | 54.23 | 5.19 |

Conclusion: Patients showed improvements in FEV_1_, FVC, MVV, and DL_CO_ before and after hUCMSC-EVs treatment.

Supplementary Table 8. Changes in 6 min walk distance before and after hUCMSC-EVs treatment

| EVs | D 0 (m) | D 8 (m) | Saline | D 0 (m) | D 8 (m) |
| --- | --- | --- | --- | --- | --- |
| 001 | 498 | 516 | 004 | 261 | 333 |
| 002 | 621 | 537 | 006 | 387 | 486 |
| 003 | 381 | 431 | 010 | 390 | 400 |
| 005 | 525 | 441 | 012 | 384 | 390 |
| 007 | 591 | 594 | 014 | 498 | 492 |
| 008 | 492 | 431 | 017 | 423 | 439 |
| 009 | 435 | 494 | 018 | 495 | 460 |
| 011 | 468 | 474 | 019 | 471 | 483 |
| 013 | 474 | 465 | 020 | 333 | 348 |
| 015 | 249 | 255 | 022 | 252 | 306 |
| 016 | 435 | 394 | 023 | 600 | 654 |
| 021 | 264 | 471 | 024 | 492 | 534 |

6 min walk distance grading criteria: <150 m indicates severe heart failure; 150–450 m indicates moderate heart failure; >450 m indicates mild heart failure.

Supplementary Table 9. Changes in the mMRC dyspnea scale, St. George's Respiratory Questionnaire (SGRQ), and Leicester Cough Questionnaire before and after hUCMSC-EVs treatment

| Grouping | Rando-mization number | Visit | mMRC Score | SGRQ Score | | | | Leicester score | | | |
| --- | --- | --- | --- | --- | --- | --- | --- | --- | --- | --- | --- |
|  |  |  |  | Part 1 | Part 2 | Part 3 | Total score | Physio-  logical | Psycho-  logical | Social | Total score |
| Treatment group | 001 | D0 | 1 | 7 | 20 | 2 | 29 | 4 | 4 | 5 | 13 |
|  |  | D8 | 1 | 7 | 14 | 1 | 22 | 5 | 5 | 5 | 15 |
|  | 002 | D0 | 0 | 14 | 14 | 1 | 29 | 5 | 5 | 4 | 14 |
|  |  | D8 | 0 | 14 | 14 | 1 | 29 | 5 | 5 | 5 | 15 |
|  | 003 | D0 | 1 | 7 | 13 | 1 | 22 | 6 | 6 | 6 | 18 |
|  |  | D8 | 1 | 7 | 12 | 1 | 20 | 6 | 6 | 6 | 18 |
|  | 005 | D0 | 1 | 7 | 14 | 1 | 22 | 4 | 5 | 5 | 14 |
|  |  | D8 | 1 | 2 | 14 | 1 | 17 | 4 | 5 | 6 | 15 |
|  | 007 | D0 | 1 | 8 | 11 | 1 | 20 | 5 | 6 | 6 | 17 |
|  |  | D8 | 1 | 11 | 12 | 1 | 24 | 5 | 5 | 6 | 16 |
|  | 008 | D0 | 2 | 26 | 23 | 2 | 51 | 1 | 2 | 2 | 5 |
|  |  | D8 | 3 | 19 | 24 | 3 | 46 | 1 | 3 | 3 | 7 |
|  | 009 | D0 | 2 | 15 | 33 | 3 | 51 | 2 | 1 | 1 | 4 |
|  |  | D8 | 2 | 11 | 21 | 1 | 33 | 3 | 1 | 1 | 5 |
|  | 011 | D0 | 2 | 13 | 13 | 0 | 26 | 5 | 5 | 6 | 16 |
|  |  | D8 | 1 | 8 | 9 | 0 | 17 | 6 | 6 | 7 | 19 |
|  | 013 | D0 | 0 | 4 | 7 | 0 | 11 | 6 | 6 | 6 | 18 |
|  |  | D8 | 0 | 1 | 2 | 0 | 3 | 7 | 7 | 7 | 21 |
|  | 015 | D0 | 3 | 24 | 28 | 2 | 54 | 3 | 3 | 3 | 9 |
|  |  | D8 | 3 | 9 | 25 | 2 | 36 | 4 | 5 | 3 | 12 |
|  | 016 | D0 | 1 | 14 | 17 | 1 | 32 | 3 | 5 | 5 | 13 |
|  |  | D8 | 1 | 6 | 16 | 1 | 23 | 5 | 5 | 5 | 15 |
|  | 021 | D0 | 2 | 29 | 35 | 2 | 66 | 1 | 1 | 1 | 3 |
|  |  | D8 | 1 | 20 | 25 | 2 | 47 | 4 | 3 | 3 | 10 |
| Control group | 004 | D0 | 3 | 13 | 27 | 3 | 43 | 5 | 6 | 6 | 17 |
|  |  | D8 | 3 | 13 | 22 | 3 | 38 | 5 | 6 | 6 | 17 |
|  | 006 | D0 | 1 | 9 | 8 | 1 | 18 | 5 | 5 | 4 | 14 |
|  |  | D8 | 1 | 16 | 25 | 1 | 42 | 4 | 1 | 1 | 6 |
|  | 010 | D0 | 1 | 13 | 23 | 1 | 37 | 4 | 4 | 4 | 12 |
|  |  | D8 | 1 | 19 | 20 | 1 | 40 | 4 | 4 | 4 | 12 |
|  | 012 | D0 | 2 | 4 | 3 | 3 | 10 | 5 | 4 | 7 | 16 |
|  |  | D8 | 2 | 2 | 7 | 3 | 12 | 6 | 7 | 7 | 20 |
|  | 014 | D0 | 1 | 3 | 13 | 1 | 17 | 7 | 7 | 7 | 21 |
|  |  | D8 | 1 | 10 | 12 | 0 | 22 | 7 | 7 | 7 | 21 |
|  | 017 | D0 | 1 | 10 | 20 | 1 | 31 | 4 | 4 | 4 | 12 |
|  |  | D8 | 1 | 19 | 18 | 1 | 38 | 4 | 4 | 4 | 12 |
|  | 018 | D0 | 0 | 8 | 5 | 0 | 13 | 4 | 4 | 6 | 14 |
|  |  | D8 | 0 | 4 | 4 | 0 | 8 | 5 | 5 | 6 | 16 |
|  | 019 | D0 | 0 | 8 | 12 | 2 | 22 | 3 | 3 | 2 | 8 |
|  |  | D8 | 0 | 8 | 16 | 1 | 25 | 6 | 5 | 4 | 15 |
|  | 020 | D0 | 3 | 8 | 17 | 1 | 26 | 3 | 2 | 3 | 8 |
|  |  | D8 | 3 | 8 | 26 | 1 | 35 | 2 | 1 | 2 | 5 |
|  | 022 | D0 | 3 | 24 | 29 | 2 | 55 | 3 | 4 | 4 | 11 |
|  |  | D8 | 3 | 27 | 27 | 2 | 56 | 4 | 3 | 4 | 11 |
|  | 023 | D0 | 0 | 4 | 11 | 1 | 16 | 4 | 6 | 4 | 14 |
|  |  | D8 | 0 | 9 | 7 | 1 | 17 | 5 | 5 | 5 | 15 |
|  | 024 | D0 | 1 | 7 | 7 | 1 | 15 | 6 | 6 | 7 | 19 |
|  |  | D8 | 1 | 2 | 1 | 0 | 3 | 6 | 6 | 6 | 18 |

Supplementary Table 10. Primer

| Col3a1 | Forward Primer | CTGTAACATGGAAACTGGGGAAA |
| --- | --- | --- |
|  | Reverse Primer | CCATAGCTGAACTGAAAACCACC |
| Col1a1 | Forward Primer | GCTCCTCTTAGGGGCCACT |
|  | Reverse Primer | CCACGTCTCACCATTGGGG |
| Fibronectin | Forward Primer | ATGTGGACCCCTCCTGATAGT |
|  | Reverse Primer | GCCCAGTGATTTCAGCAAAGG |
| miRNA-486-5P | stem-loop primer | GTCGTATCCAGTGCAGGGTCCGAGGTATTCGCACTGGATACGACCTCGGG |
|  | Forward Primer | CGCGTCCTGTACTGAGCTGC |
|  | mQ Primer R | AGTGCAGGGTCCGAGGTATT |
| IL-10_F | Forward Primer | GCTCTTACTGACTGGCATGAG |
| IL-10_R | Reverse Primer | CGCAGCTCTAGGAGCATGTG |
| Mmp13_F | Forward Primer | CTTCTTCTTGTTGAGCTGGACTC |
| Mmp13_R | Reverse Primer | CTGTGGAGGTCACTGTAGACT |
| Hgf_F | Forward Primer | ATGTGGGGGACCAAACTTCTG |
| Hgf_R | Reverse Primer | GGATGGCGACATGAAGCAG |
| Spp1_F | Forward Primer | AGCAAGAAACTCTTCCAAGCAA |
| Spp1_R | Reverse Primer | GTGAGATTCGTCAGATTCATCCG |

Supplementary Data 1. (separate file)

RNA proportion of hUCMSC-EVs

Supplementary Data 2.

All expressed miRNA of hUCMSC-EVs

Supplementary Data 3.

hUCMSC-EVs Protein

Supplementary Data 4.

hUCMSC-EVs metabolite

Supplementary Data 5.

Mouse complete blood count and serum biochemical
