# Supplementary material for: Clinical investigation on nebulized human umbilical cord MSC-derived extracellular vesicles for pulmonary fibrosis treatment
Source: Signal Transduct Target Ther. 2025 Jun 4;10:179. doi: 10.1038/s41392-025-02262-3 (PMC12134356; doi:10.1038/s41392-025-02262-3)
Supplement: Supplementary file 2 — Study protocol [file 41392_2025_2262_MOESM2_ESM.docx]

The protocol for the clinical study

# 1. Title

A Randomized, Single-Blind, Placebo-Controlled Phase I Clinical Trial to Evaluate the Safety and Efficacy of Inhaled Extracellular Vesicle Therapy for Pulmonary Fibrotic Lesions

# 2. Objective

This study aims to assess the safety and efficacy of inhaled extracellular vesicle therapy in the treatment of patients with pulmonary fibrotic lesions.

**2.1. Primary Objective**

To evaluate the safety of inhaled extracellular vesicle therapy in patients with pulmonary fibrotic lesions as identified by high-resolution computed tomography (HRCT).

1. to monitor vital signs, clinical symptoms and signs, and the incidence of adverse events on Days 1–8, Week 4, and Week 12 after enrollment.
2. to assess changes in laboratory and clinical parameters on Day 8 and Week 12, including complete blood count, urinalysis (plus pregnancy test), blood biochemistry, electrocardiogram (ECG), peripheral oxygen saturation (SpO₂), tumor markers, levels of IL-6 and IL-10, immune cell function in peripheral blood, and lymphocyte subsets.

**2.2. Secondary Objectives**

1. to evaluate changes from baseline in pulmonary function on Days 4 and 8, Week 4, and Week 12;
2. to assess HRCT imaging changes from baseline at Week 4 and Week 12;
3. to evaluate changes from baseline in six-minute walk distance (graded and actual distance) on Days 4 and 8, Week 4, and Week 12 for patients meeting criterion 5.1.2 (2) for HRCT-defined fibrotic lesions;
4. to assess changes from baseline in the St. George’s Respiratory Questionnaire (SGRQ) on Days 4 and 8, Week 4, and Week 12;
5. to assess changes from baseline in the Leicester Cough Questionnaire on Days 4 and 8, Week 4, and Week 12;
6. to assess changes from baseline in the modified Medical Research Council (mMRC) Dyspnea Scale on Days 4 and 8, Week 4, and Week 12;
7. to monitor the frequency and severity of acute exacerbations in patients with HRCT-defined pulmonary fibrotic lesions from Days 1–8, Week 4, and Week 12.

# 3. Expected Outcomes Based on the previous results from cellular and animal studies and human safety evaluations from our group, and existing literature on the therapeutic effects of stem cells and extracellular vesicles, this study is expected to demonstrate that inhalation therapy with aerosolized extracellular vesicles derived from human umbilical cord mesenchymal stem cells can improve symptoms, quality of life, and pulmonary function in patients with pulmonary fibrotic lesions, alleviate lung fibrosis, and reduce the frequency of acute exacerbation events.

# 4. Study Design

**4.1. Overview of Study Design**

This is a phase I clinical trial, designed as a single-center, randomized (1:1), single-blind, placebo-controlled clinical study.

**4.2. Dose Selection**

Based on previous animal studies and clinical data, the selected dose for this study is designed as 2×10^9^ particles per nebulization, with administration twice daily for 7 consecutive days.

**4.3. Observation Items and Indicators**

4.3.1. Screening or Baseline Data

This includes medical records of the participants prior to the enrollment. After obtaining informed consent from the participants or their legal representative, investigators will review medical records to collect the following data：

- Basic information: demographic characteristics, height, and weight;
- Pre-enrollment diagnosis, medical history, and previous treatments;
- Treatment-related information: medications;
- Vital signs and comprehensive physical examination;
- Routine tests: complete blood count (CBC), urinalysis + pregnancy test, blood biochemistry, infectious disease screening, electrocardiogram (ECG), fingertip oxygen saturation, tumor markers, IL-6 and IL-10 levels, peripheral blood immune cell function, and lymphocyte subpopulations;
- Pulmonary function tests: percentage predicted of carbon monoxide diffusion capacity (DLco), forced vital capacity (FVC), and DLCO/VA;
- Six-minute walk test (graded and distance);
- SGRQ: St. George’s Respiratory Questionnaire;
- LCQ: Leicester Cough Questionnaire;
- mMRC Dyspnea Scale: Modified Medical Research Council Dyspnea Scale;
- Chest HRCT: Chest High-Resolution Computed Tomography.

4.3.2. Post-enrollment Observation Indicators

1. Specific indicators after enrollment (see Table 1):
   - Vital signs and comprehensive physical examination;
   - Routine tests: CBC, urinalysis, blood biochemistry, ECG, fingertip oxygen saturation, tumor markers, IL-6 and IL-10 levels, peripheral blood immune cell function, lymphocyte subpopulations;
   - Pulmonary function tests: percentage predicted of DLco, FVC, and DLCO/VA;
   - Six-minute walk test (graded and distance);
   - SGRQ;
   - LCQ;
   - mMRC Dyspnea Scale;
   - Chest HRCT;
   - Acute exacerbation events (frequency and severity).
2. Adverse events during the administration period of the investigational drug. If any adverse event occurs, the patient will be followed up until resolution or end of treatment. The occurrence time and severity of complications involving various organ systems (e.g., cardiac events, cerebrovascular events, infections, etc.) will be recorded;
3. For participants discharged or deceased after enrollment, record the death event, length of hospital stay, duration of mechanical ventilation, and total treatment cost;
4. Long-term observation indicators post-enrollment (up to 1 year):
   Considering the long-term safety concerns of exosome therapy, follow-up calls will be conducted at weeks 24 and 48 to record any adverse events for long-term safety assessment.

(see next page for Table 1)

Table 1. Schedule of Assessments

| Visit | Screening  -2d～0d | Baseline  D0 | D1 | D2 | D3 | D4 | D5 | D6 | D7 | D8 | D28  ±3d | D84  ±3d | D180  ±5d | D360  ±7d |
| --- | --- | --- | --- | --- | --- | --- | --- | --- | --- | --- | --- | --- | --- | --- |
| **Basic medical history** | | |  |  |  |  |  |  |  |  |  |  |  |  |
| informed consent | × |  |  |  |  |  |  |  |  |  |  |  |  |  |
| Demographic data | × |  |  |  |  |  |  |  |  |  |  |  |  |  |
| General clinical data | × |  |  |  |  |  |  |  |  |  | × | × |  |  |
| **Security assessment** | | |  |  |  |  |  |  |  |  |  |  |  |  |
| Vital sign | × | × | × | × | × | × | × | × | × | × | × | × |  |  |
| Physical examination | × | × | × | × | × | × | × | × | × | × | × | × |  |  |
| Blood routine examination |  | × |  |  |  |  |  |  |  | × |  | × |  |  |
| urinalysis |  | × |  |  |  |  |  |  |  | × |  | × |  |  |
| Urine pregnancy test (women of gestational age only) |  | × |  |  |  |  |  |  |  | × |  | × |  |  |
| Infectious disease screening |  | × |  |  |  |  |  |  |  |  |  | × |  |  |
| Blood biochemical examination |  | × |  |  |  |  |  |  |  | × |  | × |  |  |
| Tumor marker |  | × |  |  |  |  |  |  |  |  |  | × |  |  |
| IL-6 |  | × |  |  |  |  |  |  |  | × |  | × |  |  |
| Peripheral blood lymphocyte subsets |  | × |  |  |  |  |  |  |  | × |  | × |  |  |
| ECG |  | × |  |  |  |  |  |  |  | × |  | × |  |  |
| Finger oxygen saturation |  | × | × | × | × | × | × | × | × | × | × | × |  |  |
| **Effectiveness evaluation** | | |  |  |  |  |  |  |  |  |  |  |  |  |
| Chest HRCT |  | × |  |  |  |  |  |  |  |  | × | × |  |  |
| Lung function test |  | × |  |  |  | × |  |  |  | × | × | × |  |  |
| Lung diffusion function test |  | × |  |  |  | × |  |  |  | × | × | × |  |  |
| 6MWD |  | × |  |  |  |  |  |  |  | × | × | × |  |  |
| mMRC |  | × |  |  |  |  |  |  |  | × | × | × |  |  |
| SGRQ |  | × |  |  |  |  |  |  |  | × | × | × |  |  |
| LCQ |  | × |  |  |  |  |  |  |  | × | × | × |  |  |
| Acute aggravating event |  |  | × | × | × | × | × | × | × | × | × | × | × | × |
| AE/SAE |  |  | × | × | × | × | × | × | × | × | × | × | × | × |

IL-6=interleukin 6; ECG=electrocardiogram; HRCT=High Resolution Chest Tomography; 6MWD=Six - Minute Walk Distance；SGRQ=St. George's Respiratory Questionnaire；LCQ=**Cough-specific Quality of Life Questionnaire；**mMRC=Modified Medical Research Council；AE/SAE=**Adverse Event/Serious Adverse Event.**

The terms used in this section:

mMRC Dyspnea Scale

The Modified Medical Research Council (mMRC) Dyspnea Scale (Table 2) is a single-rating scale used to assess the degree of breathlessness. This 5-grade scale considers activities that may trigger dyspnea in patients, such as walking or climbing stairs. Patients are asked to select the grade that best describes their current level of breathlessness.

Table 2. mMRC Dyspnea Scale

| **Grade** | **Description of Dyspnea** |
| --- | --- |
| 0 | Not troubled by breathlessness except during strenuous exercise. |
| 1 | Short of breath when hurrying on level ground or walking up a slight hill. |
| 2 | Walks slower than people of the same age on level ground due to breathlessness, or has to stop for breath when walking at own pace. |
| 3 | Stops for breath after walking about 100 yards or after a few minutes on level ground. |
| 4 | Too breathless to leave the house or breathless when dressing or undressing. |

Leicester Cough Questionnaire (LCQ)

The LCQ is a 19-item questionnaire designed to evaluate cough-related quality of life. It uses a 7-point Likert scale to assess symptoms or their impact over the past 2 weeks. The LCQ covers three domains: physical (8 items), psychological (7 items), and social (4 items). Scores for each domain are calculated as the mean of the respective items (range: 1–7). A total score (range: 3–21) is obtained by summing domain scores. Higher scores indicate better quality of life.

St. George’s Respiratory Questionnaire (SGRQ)

The SGRQ is a 50-item patient-reported outcome (PRO) questionnaire developed to assess health status in patients with obstructive airway diseases. It has two parts: Part 1 includes 8 questions on respiratory symptom severity over the past 4 weeks; Part 2 includes 42 questions on daily activity limitations and psychosocial impact. The SGRQ generates a total score and three component scores (Symptoms, Activity, and Impacts). The total score reflects the overall impact of the disease on health status, expressed as a percentage from 0 (best health) to 100 (worst health). Similarly, component scores range from 0 to 100, with higher scores indicating greater impairment.

# 5. Eligibility Criteria and Group Allocation Method

**5.1.** **Inclusion Criteria**

1. Age between 18 and 80 years (inclusive), no gender restriction.
2. Meets HRCT criteria for pulmonary fibrotic lesions:
   - 1. Manifestations include linear, rigid high-density shadows or nodular high-density shadows in the lungs.
     2. Manifestations include diffuse reticular, linear, honeycomb-like shadows or reticulonodular shadows in both lungs.
3. Typical HRCT imaging features of pulmonary fibrotic lesions within the past 12 weeks, including idiopathic pulmonary fibrosis, COPD with fibrosis, and chronic cough caused by organizing pneumonia post-COVID-19 infection.
4. Able to understand and cooperate with the pulmonary function test procedures.
5. Fully informed about the purpose, methods, and possible discomfort of the trial, agrees to participate, and voluntarily signs the informed consent form.
6. Good compliance, willing to follow the medication regimen as required by the protocol and attend follow-up examinations on time.

**5.2. Exclusion Criteria**

1. Previous stem cell therapy.
2. Intolerance to nebulized inhalation therapy.
3. Allergic constitution or history of potentially life-threatening drug allergies.
4. Pregnant or planning to become pregnant soon, or breastfeeding women.
5. Male participants with reproductive potential and female participants of childbearing age unwilling to use effective contraception during the treatment period and for 12 months following the end of the follow-up.
6. History of malignant tumors or systemic anti-cancer treatment within 5 years prior to the screening period.
7. Active hepatitis B or C virus infection, or HIV infection.
8. History of organ transplantation or currently awaiting organ transplantation.
9. Underwent surgery (excluding diagnostic surgery) within 8 weeks prior to enrollment, planning to undergo surgery during the study period, or with an unhealed surgical wound prior to enrollment.
10. Taking or planning to take Nintedanib or Pirfenidone within the past month.
11. Any of the following lung diseases: bronchial asthma, active pulmonary tuberculosis, pulmonary embolism, pneumothorax, pneumoconiosis, idiopathic pulmonary arterial hypertension, obliterative bronchiolitis, or other active lung diseases.
12. Current or recent (within 4 weeks) pneumonia.
13. Previous lung resection surgery.
14. Currently requiring oxygen therapy for more than 15 hours per day.
15. History of mental illness, epilepsy, or other central nervous system diseases.
16. Severe other systemic diseases such as myocardial infarction, unstable angina, heart failure, liver cirrhosis, acute glomerulonephritis, etc.
17. Participation in any other clinical trial within 3 months prior to screening.
18. Currently participating in another clinical trial.
19. Poor compliance, making it difficult to complete the study.
20. Any condition that the researcher believes may increase the risk to the participant or interfere with the study results.

**5.3. Criteria for Early Withdrawal**

Subjects will be withdrawn from the study if they request to discontinue participation or withdraw informed consent (e.g., due to poor efficacy or adverse events), or are unable to comply with study requirements. Subjects may also be withdrawn at the discretion of the investigator if serious complications or comorbidities, or severe adverse events occur.

The reason for withdrawal must be thoroughly documented. The most recent major efficacy assessment before withdrawal will be used as the final result for statistical analysis. The subject’s CRF (Case Report Form) must be retained for future reference.

**5.4. Criteria for Exclusion from Analysis**

Subjects who have already been enrolled but meet any of the following criteria should be excluded from the final analysis:

1. The subject never received the investigational drug.
2. There are no valid assessment records.
3. The subject used a prohibited medication, making efficacy and safety evaluation impossible.

Reasons for exclusion must be stated clearly. Excluded cases will not be included in efficacy analyses. Their CRFs should still be preserved for future reference.

**5.5. Group Allocation Method**

5.5.1. Randomization Method

Randomization codes in a 1:1 ratio for the treatment and placebo groups will be generated using the SAS statistical software package by a statistician who is not involved in data management or analysis. A block randomization design will be used, with each block including one subject in the exosome treatment group and one in the placebo group.

Drug assignment codes will be created by a statistician unaffiliated with the study. The randomization system will be available 24 hours a day. Investigational drugs will be administered according to the order of enrollment using corresponding random numbers.

The randomization codes (sealed blind codes) will be prepared in duplicate and securely stored by the principal investigator and the clinical trial pharmacy at the study institution.

Randomization and blinding will be guided by the Biostatistics Department of Hainan Medical University.

5.5.2. Blinding

One research coordinator will be designated to maintain and distribute randomization numbers, prepare investigational drugs, and coordinate information among the study staff.

One study physician will be designated for patient management and data recording.
One study nurse will be responsible for administering the investigational product and monitoring safety.

Trained physicians will follow up with patients and perform additional diagnostics.

All members of the research team will remain blinded to each other’s assessments throughout the study.

5.5.3. Investigational Exosome Preparation (Derived from Human Umbilical Cord Mesenchymal Stem Cells)

Exosome nebulization solution: 2 ml/vial, containing 2×10⁹ particles.

The investigational exosome product is provided by Shanghai GenWay Cell & Tissue Storage Co., Ltd. It is derived from mesenchymal stem cells (MSCs) cultured and purified from donated neonatal umbilical cord tissue (see Section 5-0 for donor screening criteria and informed consent form, and Section 5-1 for perinatal tissue handover information). The MSC supernatant is further purified to obtain exosomes.

Informed consent is obtained from all healthy donors. Any remaining investigational products will be uniformly retrieved and destroyed after study completion.

A dedicated management system is established for the storage and distribution of investigational products. Products are delivered directly to designated personnel in the clinical research department by staff from Shanghai GenWay Cell & Tissue Storage Co., Ltd., with proper documentation for product handover.

# 6. Sample Size Requirement

This study is a randomized, placebo-controlled, exploratory clinical trial. A total of 24 subjects will be enrolled at the First Affiliated Hospital of Hainan Medical University, with 12 subjects assigned to the treatment group and 12 to the placebo group.

# Method of Administration, Dosage, Timing, and Course of Treatment for the Extracellular Vesicle (EV) Preparation

**7.1. Registration, Usage Records, Delivery, Distribution Method, and Storage Conditions of the EV Nebulized Solution**

1. After the EV product is fully prepared and passes quality control, laboratory personnel shall complete the **EV Nebulization Application Form** and submit it to the investigators. Both parties confirm relevant information about the EV product (including subject name, hospital admission number, EV product batch number, and quality inspection report), agree on the nebulization schedule, and sign for confirmation.
2. Laboratory personnel shall complete the EV Nebulized Solution Release Application Form, which must be signed by the quality control officer. The form is then submitted to the warehouse manager. Upon reconfirming the relevant EV nebulized solution information (including subject name, hospital admission number, EV nebulization batch number, and quality inspection report), both parties sign the EV Nebulized Solution Release and Handover Form. The refrigerated box containing the EV nebulized solution is then handed over to the laboratory personnel.
3. The laboratory personnel transport the refrigerated box containing the EV nebulized solution to the laminar flow ward. Together with the investigators, they reconfirm the EV nebulized solution information (including subject name, hospital admission number, batch number, and quality inspection report) and sign the EV Nebulized Solution Handover Record Form, after which the solution is handed over to the investigators.
4. **Storage condition:** 2°C–8°C.

**7.2. Dosage and Administration**

Based on results from animal studies, prior volunteer applications, and relevant literature, the administration method is as follows:

Investigational Product

- Name: Human umbilical cord mesenchymal stem cell-derived extracellular vesicle nebulized solution
- Specification: 2 ml: 2×10⁹ particles
- Storage condition: 2°C–8°C
- Dosage and administration: Inhalation twice daily, 2 ml each time, diluted with 6 ml of normal saline and administered via nebulization at 09:00 ± 30 min in the morning and 20:00 ± 30 min in the evening, for a 7-day treatment course.

Placebo Control

- Name: Normal saline
- Specification: 8 ml
- Storage condition: Store below 25°C, avoid direct sunlight, high temperature, or freezing
- Dosage and administration: Inhalation twice daily, 8 ml each time via nebulization at 09:00 ± 30 min in the morning and 20:00 ± 30 min in the evening, for a 7-day treatment course.

To ensure patient safety, the first 4 subjects will be enrolled sequentially. Each subject will be observed for 24 hours after the initial treatment. If no adverse reactions are noted, the next subject will begin treatment. After all 4 subjects have completed the treatment, a safety assessment will be conducted before full clinical trial rollout.

**7.3. Time of Use**

Nebulization is scheduled at 09:00 ± 30 min in the morning and 20:00 ± 30 min in the evening each day.

**7.4. Course of Treatment**

One course lasts 7 days, and it will not be repeated.

# 8 Criteria for Suspension and Termination of the Clinical Study

**8.1. Criteria for Participant Withdrawal or Study Termination**

1. The subject withdraws informed consent and requests to discontinue participation.
2. The subject exhibits significant non-compliance.
3. Occurrence of pregnancy, serious adverse events, death, or loss to follow-up.
4. Use of prohibited concomitant medications.
5. The subject fails to attend scheduled visits.
6. Investigators should make every effort to ensure that each subject completes the follow-up phase, unless discontinuation is clearly in the subject's best interest. Subjects who withdraw from the study should still enter the follow-up phase and will receive periodic follow-ups to monitor survival status until death or the end of the study.

**8.2. Early Study Suspension or Termination by Investigators**

The investigator should suspend or terminate the study early if any of the following conditions are met:

1. Violation of inclusion criteria or fulfillment of exclusion criteria.
2. The investigator determines that the trial should not continue due to adverse events.
3. Serious safety issues arise during the study (e.g., serious adverse reactions, severe complications, or rapid disease progression).
4. The treatment is found to be ineffective or of low efficacy during the study, lacking clinical value and potentially delaying appropriate treatment.
5. Major flaws are found in the study protocol or significant deviations occur during implementation, making evaluation unreliable.
6. Other reasons deemed necessary by the investigator to suspend or terminate treatment.

# 9. Efficacy Evaluation Criteria

**9.1. Efficacy Assessment**

(1) Primary Efficacy Endpoints

- Clinical symptoms and signs
- Pulmonary function
- High-resolution chest CT (HRCT)
- Frequency and severity of acute exacerbation events

(2) Secondary Endpoints

- 6-minute walk distance (6MWD), including grade and distance
- St. George's Respiratory Questionnaire (SGRQ)
- Leicester Cough Questionnaire
- Modified Medical Research Council Dyspnea Scale (mMRC)

(3) Other Observational Indicators

- Post-treatment changes in IL-6 levels, peripheral blood immune cell function, and peripheral blood lymphocyte subsets.

**9.2. Safety Assessment**

Both short-term and long-term safety assessments are included. All adverse events (AEs), including serious adverse events (SAEs), should be monitored and recorded. Detailed records should be maintained for vital signs, clinical symptoms and signs, routine blood tests, urinalysis with pregnancy test, infectious disease screening, blood biochemistry, electrocardiogram (ECG), peripheral oxygen saturation, tumor markers, IL-6, IL-10 levels, immune cell function, and lymphocyte subsets.

1. Short-term (In-Hospital) Safety Monitoring

- Physical examination: including BMI, heart rate, blood pressure, respiratory rate, fingertip oxygen saturation, and complete physical examination.
- Blood tests: including complete blood count (CBC), urinalysis with pregnancy test, infectious disease screening, blood biochemistry, tumor markers, and IL-6, IL-10 levels.
- Auxiliary tests: including ECG and chest HRCT.
- Monitor and record any adverse events during administration of the investigational product. If adverse events occur, follow up with the patient until resolution or end of treatment.

1. Long-term Safety Evaluation

Long-term safety will be evaluated by monitoring the occurrence of adverse events (AEs) or serious adverse events (SAEs) at 6 and 12 months after treatment with extracellular vesicles.

# 10. Recording Requirements for Adverse Events and Reporting/ Management of Serious Adverse Events

**10.1. Adverse Events (AE)**

1. An AE refers to any untoward medical occurrence in a participant during the course of the study, regardless of its causal relationship with the investigational product. It includes any unfavorable or unintended sign (including abnormal laboratory findings), symptom, or disease temporarily associated with the use of the investigational product, whether or not considered related to it.
2. All AEs, regardless of their relationship to the investigational product, must be recorded in the Case Report Form (CRF), including the diagnosis, onset and duration, interventions taken, treatment interruption status, corrective measures, outcome, and other possible causes.
3. All AEs occurring during the observation period—from the date of informed consent signing to study completion—must be collected, including:
4. Exacerbation of pre-existing symptoms or diseases prior to the trial (excluding the disease intended to be treated by the investigational extracellular vesicles).
5. Increased frequency or severity of pre-existing episodic events.
6. Abnormal changes detected or diagnosed after administration of the investigational extracellular vesicles, even if such abnormalities may have existed before treatment.
7. Not all abnormal lab results or other test findings (e.g., ECG or vital signs) are reported as AEs unless associated with clinical symptoms, result in treatment changes, require medical intervention, or are deemed clinically significant by the investigator. Such findings, if considered part of a clinical diagnosis or corresponding standard medical terminology (reported as an AE), should not be listed separately as AEs but reported as the diagnosis or term (e.g., anemia).

**10.2. Severity Grading of Adverse Events**

The severity of AEs should be graded using the Common Terminology Criteria for Adverse Events (CTCAE) Version 5.0 by the U.S. National Cancer Institute (NCI).

**10.3. Assessment of Relationship Between AE and Investigational Product**

1. Investigators should evaluate the potential association between the AE and the investigational product based on the following criteria:
   1. Time of onset and time to resolution.
   2. Grading per NCI-CTCAE v5.0 definitions.
   3. Causality categorized as: definitely related, probably related, possibly unrelated, unrelated, or indeterminate. The first three categories (definitely, probably, and indeterminate) are considered related to the investigational product and will be used for calculating incidence rates of adverse reactions, with the number of such events as numerator and the total number of participants as denominator for safety evaluation.
2. All AEs or other symptoms and signs occurring during the study must be recorded on the AE page of the CRF, regardless of their relationship to the investigational treatment.
3. Any participant who receives treatment must be evaluated for toxic reactions. The severity of any AEs should be graded according to NCI-CTCAE v5.0.

**10.4. Serious Adverse Events (SAE)**

1. An SAE is defined as an AE that meets any of the following criteria:
   1. Death not directly caused by the underlying disease or its complications.
   2. Life-threatening or potentially fatal.
   3. Requires hospitalization or prolongs existing hospitalization (complications occurring during hospitalization are considered AEs; complications prolonging hospitalization are considered SAEs).
   4. Results in persistent or significant disability or incapacity that affects the ability to work.
   5. Causes congenital anomaly or birth defect.
   6. Considered a medically important event by the investigator.
2. Special considerations for handling SAEs:
   1. Disease progression during the study or reporting period (including symptoms and signs of progression) should not be reported as SAEs.
   2. Death, hospitalization/prolongation, or disability caused by the disease itself or its direct complications are not considered SAEs.
   3. Hospitalizations due to AEs or prolonged hospital stays caused by AEs during the study are SAEs. Any initial admission to a medical institution (even if less than 24 hours) meets the criteria. Hospitalizations unrelated to AE worsening (e.g., routine workups for persistent pre-study lab abnormalities) are not considered SAEs.

**10.5. AE Documentation and Reporting**

All AEs should be reported to relevant institutional departments. If a serious, unexpected AE related to the study occurs that falls within the scope of the hospital’s clinical trial liability insurance, compensation for the patient will be sought through the insurer.

- All AEs: Interventions should be promptly taken and recorded in the CRF.
- SAEs: Immediate interventions are required. The event must be documented in the CRF and reported to the Ethics Committee, Clinical Trial Institution, and manufacturing unit. Additionally, it must be reported within 24 hours to national and provincial health authorities and the food and drug regulatory administration.

**10.6. AE Management and Follow-up**

All equipment, reagents, and techniques used in the preparation of the aerosolized EV solution follow national preparation standards and undergo strict quality control before patient administration.

Adverse reactions to aerosolized EV treatment are rare. A small number of patients may experience mild symptoms such as flushing, rash, low-grade fever, chills, nausea, or vomiting, requiring symptomatic treatment. In rare cases, patients may develop serious symptoms such as dyspnea, hypotension, tachycardia, or severe allergic reactions, necessitating immediate medical intervention.

Potential AEs and Management:：

1. Gastrointestinal reactions: 1) Use 5-HT3 receptor antagonists, corticosteroids, NK1 receptor antagonists to prevent/treat vomiting. 2) Use antidiarrheals, mucosal protectants, and intestinal antibiotics to control symptoms, promote healing, and prevent secondary infection.
2. Cardiotoxicity: 1) Avoid fluid overload; 2) Administer diuretics if needed; 3) Closely monitor ECG before, during, and 24 hours after treatment.
3. Lack of efficacy: If nebulized EVs do not alleviate symptoms or improve lung tissue repair, this has been clearly explained in the informed consent form.
4. Fever: Administer antipyretics such as acetaminophen. If persistent, investigate bacterial infection and treat accordingly.
5. Infection prevention: All EV preparation and nebulization must follow strict protocols to reduce infection risk.
6. Pain or discomfort during nebulization: Provide symptomatic relief as needed.
7. Dyspnea or mild pneumonia post-nebulization: Though rare, active treatment under close observation will be provided.
8. Cerebral embolism: Extremely rare but should remain on the alert.
9. Allergic reactions: For rash or itching: slow infusion or pause; administer 5–10 mg dexamethasone IV and/or 25–50 mg promethazine IM. If symptoms persist or worsen, discontinue nebulization and initiate anti-allergy treatment. For anaphylactic shock: stop nebulization immediately, lay the patient flat, and keep warm. Subcutaneously inject 0.5–1 ml of 0.1% epinephrine; repeat every 30 min if necessary. Administer oxygen therapy; if respiration is inhibited, use mechanical ventilation. For laryngeal edema, perform intubation or tracheotomy. Administer dexamethasone 5–10 mg IV or hydrocortisone 200 mg with 500 ml 5%/10% glucose IV. Use pressor agents like dopamine, norepinephrine, and antihistamines like methylene blue or promethazine IM. For cardiac arrest, initiate CPR and correct acidosis promptly. Closely monitor and document vital signs, urine output, and clinical changes. Patients in critical condition should not be moved.
10. Chills: Administer 25–50 mg promethazine IM during chill episodes.
11. General discomfort: Allow more rest and administer anxiolytics such as diazepam or chlorpromazine as appropriate.
12. All AEs must be followed up until resolution or loss to follow-up. AE resolution is defined as return to baseline health or when the investigator deems no further improvement or deterioration is expected. Follow-up methods may include hospitalization, outpatient visits, home visits, telephone calls, or written communication, depending on severity.

# 11. Statistical Analysis of Study Results

**11.1. Dataset Definitions**

- 1. Full Analysis Set (FAS)

All randomized subjects, regardless of whether they received aerosolized EV treatment are included. Analysis will be conducted according to the Intention-To-Treat (ITT) principle.

- 1. Per-Protocol Set (PPS)

Randomized subjects who received at least one dose of the assigned aerosolized EV treatment and had no major protocol deviations, as well as control subjects who did not receive EV aerosol treatment are included. Criteria for exclusion from the PPS will be defined prior to database lock and will be detailed in the statistical analysis plan.

- 1. Safety Analysis Set (SAS)

Randomized subjects who received at least one dose of aerosolized EVs are included. This set will be used for all safety evaluations. Subjects who were not randomized but received the study treatment will have their safety data listed separately and used only for overall safety evaluation of the study treatment.

**11.2. General Principles**

The efficacy report of this study, including both primary and secondary efficacy endpoints, will be based on the Intention-To-Treat (ITT) principle. The analysis strategies are as follows:

1. Quantitative variables will be described using mean (standard deviation), median (minimum, maximum, or interquartile range). Categorical variables will be presented as counts (percentages).
2. All statistical tests will be two-sided. A p-value ≤ 0.05 will be considered statistically significant.
3. Enrollment and Completion Status

The number of enrolled and completed cases will be summarized, and a list of dropout cases will be provided. The total dropout rates between groups will be compared using the chi-square test.

**11.3. General and Baseline Characteristics**

(1) Demographic information, prior medication history, and comorbidities will be descriptively summarized.

(2) For quantitative variables like age, baseline comparisons between groups will be performed using grouped t-tests or Wilcoxon rank-sum tests, depending on the characteristics of the variables. For categorical variables like gender or comorbidities, comparisons will be made using chi-square or Fisher's exact tests.

**11.4. Efficacy Evaluation**

(1) Primary Efficacy Evaluation:

The proportion of patients who experienced disease progression events within 84 days after enrollment or by the end of follow-up will be calculated and compared between groups using the chi-square test.

(2) Secondary Efficacy Evaluation:

Proportions of individual disease progression events will be compared using chi-square or Fisher's exact tests. Time to disease recovery and symptom improvement will be analyzed using grouped t-tests or Wilcoxon rank-sum tests.

(3) Other Observational Indicators:

Changes in IL-6 levels post treatment will be compared between groups using repeated measures analysis. Comparisons at different time points will be made using t-tests. Quality of life scores and total hospitalization costs will also be compared using t-tests or Wilcoxon rank-sum tests.

**11.5. Safety Analysis**

A detailed listing of all adverse events occurring during the trial will be provided. Changes in laboratory results (normal/abnormal) before and after the trial and their relationship to the study drug will be assessed. The incidence of adverse events in each group will be summarized. Safety analyses will be based on the Safety Analysis Set and conducted by treatment group. Treatment-emergent adverse events (TEAEs) and serious adverse events (SAEs) will be summarized. Descriptive statistics will be used to summarize laboratory findings, symptoms, vital signs, physical examinations, and ultrasound results. Abnormal values will be flagged.

# 12. Follow-up Plan and Implementation

All case follow-up personnel in this trial received standardized training and guidance prior to the start of the study.

**12.1. Treatment Observation Period and Follow-up Methods**

Safety and efficacy observations will be conducted according to the study schedule. Additional safety follow-ups will be conducted via telephone visits at 6 and 12 months.

**12.2. Follow-up Content**

Follow-up assessments will be conducted according to the items marked in the clinical study flowchart. Detailed test items and methods are described in Section 4 – Study Design of the clinical trial protocol.

**12.3. Withdrawal from Study During Follow-up**

Subjects or their legal representatives have the right to withdraw from the study at any time and for any reason. Investigators also have the right to terminate a subject’s participation in the study. If a subject withdraws, the reason must be recorded in the Case Report Form (CRF).
