# Supplementary material for: Clinical investigation on nebulized human umbilical cord MSC-derived extracellular vesicles for pulmonary fibrosis treatment
Source: Signal Transduct Target Ther. 2025 Jun 4;10:179. doi: 10.1038/s41392-025-02262-3 (PMC12134356; doi:10.1038/s41392-025-02262-3)
Supplement: Supplementary file 10 — Informed Consent Form 008 [file 41392_2025_2262_MOESM10_ESM.pdf]

Medical record No:

A randomized, single-blind, placebo-controlled, phase I clinical study of the safety and efficacy of nebulized exosomes of hum an umbilical mesenchymal stem cells in the treatment of pulmonary fibrosis manifested by HRCT

Informed Consent Form

Version number/version date：**V4.0/20231210**

Setting: The First Affiliated Hospital of Hainan Medical University

Collaborating institution: Tsinghua University and Shanghai Jinwei Cell and Tissue Storage Service Co., Ltd.

Informed Consent Form

Part one: Participant information

We are conducting a randomized, single-blind, placebo-controlled Phase I clinical study to assess the safety and efficacy of exosome nebulization therapy for pulmonary fibrotic lesions. Since your condition may meet the eligibility criteria for this study, we would like to invite you to participate. This informed consent form will provide you with information about the study’s purpose, procedures, potential benefits, risks, and costs. Please read it carefully before making a decision about whether or not to participate. During the explanation and discussion of the consent form, you are free to ask any questions and have the research staff clarify any points you do not understand. You may also discuss your decision with family, friends, or your doctor before making a final choice.

If you are currently participating in other clinical studies, please inform the research team.

The principal investigators of this study are Professors Huang Huaping and Hao Xinbao from the First Affiliated Hospital of Hainan Medical University. The collaborating research institutions include Tsinghua University and Shanghai GeneCell Bioscience Co., Ltd.

1. Why is this study being conducted?

Currently, there are no effective clinical treatments for pulmonary fibrotic lesions caused by infections and various other factors. Therefore, addressing the issue of lung tissue repair in pulmonary fibrotic lesions, improving patients' quality of life, and extending life expectancy are urgent challenges for clinicians.

At present, we need to conduct a small-sample, randomized controlled clinical study to assess the safety and efficacy of exosome nebulization therapy in patients with pulmonary fibrotic lesions.

2. Who will be invited to participate in this study?

If you decide to participate in this study, you will need to undergo a pre-specified screening assessment to confirm whether you meet the criteria for this clinical trial. The screening will include questions about your medical history, medication history, allergy history, as well as a physical examination, blood tests, electrocardiogram, and high-resolution chest CT scans. If you pass the screening and have no contraindications for participating in the study, you will be enrolled, with an observation period of 4 weeks/12 weeks, followed by phone follow-ups at 24 weeks and 48 weeks after enrollment.

This study involves a new treatment method, and its effects on pregnant women and fetuses are unknown. Therefore, participants must use contraception during the study if they engage in sexual activity.

Inclusion criteria

1. Age between 18 and 80 years (inclusive), no gender restriction.
2. Meets HRCT criteria for pulmonary fibrotic lesions:
   a) Manifestations include linear, rigid high-density shadows or nodular high-density shadows in the lungs.
   b) Manifestations include diffuse reticular, linear, honeycomb-like shadows or reticulonodular shadows in both lungs.
3. Typical HRCT imaging features of pulmonary fibrotic lesions within the past 12 weeks, including idiopathic pulmonary fibrosis, COPD with fibrosis, and chronic cough caused by organizing pneumonia post-COVID-19 infection.
4. Able to understand and cooperate with the pulmonary function test procedures.
5. Fully informed about the purpose, methods, and possible discomfort of the trial, agrees to

participate, and voluntarily signs the informed consent form.

6. Good compliance, willing to follow the medication regimen as required by the protocol and attend follow-up examinations on time.

Exclusion criteria:

1. Previous stem cell therapy.
2. Intolerance to nebulized inhalation therapy.
3. Allergic constitution or history of potentially life-threatening drug allergies.
4. Pregnant or planning to become pregnant soon, or breastfeeding women.
5. Male participants with reproductive potential and female participants of childbearing age unwilling to use effective contraception during the treatment period and for 12 months following the end of the follow-up.
6. History of malignant tumors or systemic anti-cancer treatment within 5 years prior to the screening period.
7. Active hepatitis B or C virus infection, or HIV infection.
8. History of organ transplantation or currently awaiting organ transplantation.
9. Underwent surgery (excluding diagnostic surgery) within 8 weeks prior to enrollment, planning to undergo surgery during the study period, or with an unhealed surgical wound prior to enrollment.
10. Taking or planning to take nintedanib or pirfenidone within the past month.
11. Any of the following lung diseases: bronchial asthma, active pulmonary tuberculosis, pulmonary embolism, pneumothorax, pneumoconiosis, idiopathic pulmonary arterial hypertension, obliterative bronchiolitis, or other active lung diseases.
12. Current or recent (within 4 weeks) pneumonia.
13. Previous lung resection surgery.
14. Currently requiring oxygen therapy for more than 15 hours per day.
15. History of mental illness, epilepsy, or other central nervous system diseases.
16. Severe other systemic diseases such as myocardial infarction, unstable angina, heart failure, liver cirrhosis, acute glomerulonephritis, etc.
17. Participation in any other clinical trial within 3 months prior to screening.
18. Currently participating in another clinical trial.
19. Poor compliance, making it difficult to complete the study.
20. Any condition that the researcher believes may increase the risk to the participant or interfere with the study results.

3. How many people will participate in this study?

This study plans to recruit 24 participants for a single-center, randomized, single-blind, placebo-controlled clinical trial to assess the safety and efficacy of exosome inhalation therapy in patients with pulmonary fibrotic lesions.

4. How will the study be conducted?

A total of 24 participants will be enrolled and treated with exosome nebulization. The placebo is a substance resembling the study drug in appearance but has no therapeutic effect.
Administration method: nebulization;

Dosage and frequency: 2 ml per dose (containing 2×10^9 particles), twice daily;
Treatment duration: 7 days.
This is a randomized controlled trial, with participants randomly assigned to the treatment and control groups at a 1:1 ratio. You will not know which group you are in until the study ends. The study consists of three phases: screening, treatment, and follow-up. The detailed process is shown in the clinical trial flowchart (Table 1).

5. How will participating in this study affect participants' daily lives?

- When deciding whether to participate in this study, please carefully consider the potential impact of the tests and follow-ups on your daily work, family life, and other responsibilities. Also, think about the time and transportation involved for each visit. If you have any questions about the tests or procedures involved, feel free to ask us.
- Please consult your study doctor before taking any new medications.
- For your safety and to ensure the validity of the study results, you cannot participate in any other clinical studies involving drugs or medical devices during the course of this study.

6. Risks and adverse reactions for participants in this study
The researchers will monitor for any side effects of exosome nebulization. During the trial, if you experience any side effects or discomfort, it is crucial that you report them immediately to the research team. The researchers may provide additional medications to alleviate these side effects or discomforts. If you or the researchers believe you cannot tolerate the side effects, the study medication may be discontinued, and you may withdraw from the study.

Possible risks of exosome therapy:
The exosomes used in this study are produced by Shanghai GeneCell Bioscience Co., Ltd., which has received certification for compliance with Good Laboratory Practice (GLP) for non-clinical drug studies. The equipment, reagents, and techniques used in the preparation of the exosome nebulization solution all meet national standards for drug preparation, and only products that pass rigorous quality testing will be administered to patients. During treatment, participants may experience the following adverse reactions:

1. A few patients may experience mild symptoms such as flushing, rash, low-grade fever, chills, nausea, and vomiting, which can be alleviated with symptomatic treatment.
2. A very small number of patients may experience more severe symptoms such as difficulty breathing, low blood pressure, tachycardia, or severe allergic reactions, in which case immediate and active treatment will be administered until the symptoms are relieved.
3. There is a risk of pregnancy during the study. The effects of exosome therapy on pregnant women and fetuses are currently unknown. In the case of accidental pregnancy, there may be risks of miscarriage or fetal malformation.

We will closely monitor you for any adverse events or reactions that may occur. If you experience any discomfort, changes in your condition, or unexpected situations during the study—whether or not they are related to the treatment—you should promptly inform your doctor, who will make an assessment and provide appropriate medical care.

There is also a possibility that the treatment may be ineffective, and your condition may continue to worsen due to treatment failure or the presence of other underlying diseases. If the doctor determines that the treatment used in this study is ineffective, the study will be immediately terminated, and other potentially effective treatments will be used.

Radiation risks:
During this clinical trial, you will undergo radiation-based examinations due to clinical testing requirements. Since radiation can produce biological effects on the body, the doctors will minimize your exposure to radiation while meeting the needs of the study. The radiation dose will remain within the allowable limits set by national and international regulations.

Electrocardiogram (ECG):
You will need to undergo a 12-lead electrocardiogram during this trial. The potential risk is skin irritation from the electrodes or discomfort when removing the electrodes.

Blood pressure:
The cuff used to measure blood pressure may cause discomfort or bruising on your arm.

Blood draw risks:
The risks associated with blood draws from the arm include temporary discomfort and/or bruising. Though unlikely, there is also a risk of infection, excessive bleeding, clotting, or fainting.

Reproductive risks:

- For female participants: If you are breastfeeding, pregnant, think you might be pregnant, or are planning to become pregnant, you cannot participate in this study. To participate, you must use contraception until the end of the study. If you are sexually active, you must use a contraceptive method that is acceptable to both you and the research team. If you become pregnant or think you might be pregnant during the study, it is crucial to inform the research team immediately. If you become pregnant, you will be withdrawn from the study, and the research team will discuss your next steps with you.
- For male participants: Participation in this study may harm your sperm, which could affect a child conceived during the study. This harm is currently unpredictable. Please inform your partner of this risk to any potential unborn children. She should be aware that if she becomes pregnant, you must immediately inform the research team, and she should inform her doctor as well.

Other risks:
Because the study involves completing the SGRQ quality of life questionnaire, it may cause some psychological discomfort. If any questions make you feel uncomfortable, you may choose not to answer them. Additionally, there may be risks, discomforts, drug interactions, or adverse reactions that are currently unforeseeable.

7. Possible benefits for participants in this study
By participating in this study, you may potentially benefit, but we cannot guarantee an improvement in

your health condition. You may not receive any direct benefits from participating. However, the information gained from this study may hold scientific value and contribute to the development of new treatments, possibly benefiting others with similar needs.

8. Are there alternative treatments if I do not participate in this study?

You can choose not to participate in this study, and this will not negatively affect your access to standard medical treatment. Currently, there are no effective treatments for HRCT-diagnosed pulmonary fibrosis.

9. Do I have to participate and complete this study?

Your participation in this study is entirely voluntary. If you do not wish to participate, you can refuse, and this will not affect your current or future healthcare in any way. Even if you agree to participate, you can change your mind and withdraw from the study at any time, without facing discrimination or retaliation, and this will not impact your access to regular medical services. If you decide to withdraw, please inform the research team promptly so they can provide guidance and support for your health.

You may also be withdrawn from the study under the following circumstances:

1. You voluntarily choose to leave the study.
2. You experience a Grade III or higher adverse event deemed related to the exosome treatment.
3. You exhibit severe non-compliance.
4. Pregnancy, a severe adverse event, death, or loss of contact occurs.
5. The researchers believe there are other necessary reasons for withdrawal.

For participants who withdraw, the research team will attempt to continue follow-up monitoring for safety reasons, but you have the right to decline further follow-ups. After withdrawal, you will still be in the follow-up phase and receive periodic check-ups to monitor your survival status.

The clinical researchers or regulatory authorities may also terminate the study during its course. If the study is terminated early, we will inform you promptly, and your research team will advise you on further treatment plans based on your health condition.

In principle, after you withdraw, your information will be securely stored until final destruction and will not be further used or disclosed. However, in rare cases, the researchers may need to continue using or disclosing your information, even after you have withdrawn or the study has ended. These cases include: removal of your information may compromise the scientific validity or safety assessment of the data, or limited information may be needed for research, teaching, or other activities (without revealing your name, ID number, or other personally identifiable information). If government regulators require oversight of the study, they may request to view all study-related information, including your data from when you participated.

10. Study participation costs

You will receive the study drug and any required tests free of charge as per the study protocol. There will be no additional charges for the study drug or any study-related procedures. You will not be

charged any other fees for participating in this study. Additionally, we will provide you with travel reimbursement for your visits, totaling ¥600. The specific breakdown is as follows:

- ¥200 for completing the screening visit.
- ¥200 for completing the baseline visit (random assignment).
- ¥200 for completing the end-of-treatment visit.

11. Handling of study-related injuries

If your health is harmed during your participation in this study, please inform the research team (Contact: [ ] and phone number: [ ]). According to Chinese regulations, if any study-related harm occurs, we will immediately take active medical measures for treatment, and compensation will be provided according to the current laws (medical and related costs will be covered by the sponsor). If unexpected serious adverse events occur due to the study, which align with the terms of the clinical trial liability insurance (which has been purchased by the sponsor), compensation will be provided by the insurance company.

12. What is required of me if I participate?

- Provide accurate information about your medical history and current health status.
- Inform the research team of any health issues you experience during the study.
- Inform the research team of any new medications, drugs, vitamins, or herbal remedies you take during the study.
- Do not take any medications or treatments, including prescription drugs and over-the-counter products (including vitamins and herbs), without the research team's approval.
- Use the study medication as directed and attend all required visits.
- Do not participate in other medical studies.
- Use appropriate contraception throughout the study.
- Follow the instructions of the research team.
- If you have any questions, feel free to ask at any time.

13. Will personal information of participants be kept confidential?

If you decide to participate in this study, your participation and personal data will be kept confidential. Your blood/urine samples will be labeled with a study ID number instead of your name. Identifiable information about you will not be disclosed to anyone outside the research team unless you give your permission. All members of the research team are required to keep your identity confidential. Your records will be stored in a locked file cabinet and will only be accessible to the research team. To ensure the study is conducted according to regulations, government authorities or ethics committee members may access your personal data at the study site if necessary. When the results of this study are published, no personal information about you will be disclosed.

14. Will you be informed of new information related to the study?

During the trial, we may learn new information about the treatment. We will notify you promptly, so you can decide whether to continue participating or withdraw from the study.

15. Who should be contacted if there are questions or issues?

If you have any questions related to this study, please contact Dr. [ ] at [ ].

If you have questions regarding your rights as a participant, you may contact the Ethics Committee of the First Affiliated Hospital of Hainan Medical University at 0898-66735891.

Table 1. Clinical research flowchart

Visit	Screening Period	Randomization	Treatment observation period							Post-treatment assessment period			Telephone follow-up period	
Time window	24h-48h	D_0	D_1	D_2	D_3	D_4	D_5	D_6	D_7	D_8	D_{4w}	D_{12w}	D_{6m}	D_{12m}
Number of visits	Visit 1	Visit 2	Visit 3	Visit 4	Visit 5	Visit 6	Visit 7	Visit 8	Visit 9	Visit 10	Visit 11	Visit 12	Visit 13	Visit 14
Informed consent form	√													
Inclusion/Exclusion Criteria	√	√												
Height/Weight	√										√	√		
Body temperature	√	√	√	√	√	√	√	√	√	√	√	√		
Respiratory rate	√	√	√	√	√	√	√	√	√	√	√	√		
Physical examination	√	√	√	√	√	√	√	√	√	√	√	√		
Blood routine examination		√								√		√		
Urine routine and pregnancy test		√								√		√		
Infectious disease screening		√										√		
blood biochemistry [a]		√								√		√		
tumor marker		√										√		
IL-6、IL-10		√										√		
Peripheral blood lymphocyte subsets		√										√		
Chest HRCT		√									√	√		

ECG		√								√		√		
Oxygen saturation at the fingertips		√	√	√	√	√	√	√	√	√	√	√		
lung function		√								√	√	√		
6 min walk distance		√								√	√	√		
St George's Respiratory Questionnaire （SGRQ）		√								√	√	√		
Leicester Cough Questionnaire		√								√	√	√		
modified Medical Research Council		√								√	√	√		
Acute exacerbation events			√	√	√	√	√	√	√	√	√	√		
Disease progression events [c]			√	√	√	√	√	√	√	√	√	√		
Disease outcomes			√	√	√	√	√	√	√	√	√	√		
AE/SAE			√	√	√	√	√	√	√	√	√	√	√	√

a) Including liver and kidney function, blood glucose, blood lipid

Informed consent ·Consent signature page

Statement of informed consent of the subjects

I have been informed about the background, objectives, procedures, risks, and benefits of the randomized, single-blind, placebo-controlled phase I clinical study on the safety and efficacy of aerosolized exosome therapy for fibrotic lung lesions. I have had ample time and opportunity to ask questions, and I am satisfied with the answers provided. I have also been informed about whom to contact if I have further questions or need more information. I have read this informed consent form and agree to participate in this study. I understand that I may withdraw from the study at any time without providing a reason. I am also aware that if I withdraw from the study, particularly due to drug-related reasons, informing the doctor of any changes in my condition and completing the necessary physical and laboratory examinations will be beneficial for both myself and the overall study. I agree to allow the drug regulatory authorities, the ethics committee, or representatives of the sponsor to review my study data.

Finally, I agree to participate in this study and follow the medical instructions. I have been informed that I will receive a copy of this informed consent form, which will include the signatures of both myself and the researchers.

Subject's signature: Date：

Signature of legal representative [if applicable]: Date：

Relationship with subjects:

Investigator informed statement

I have informed the subject (and their legal representative) about the background, objectives, procedures, risks, and benefits of the clinical study on the safety and efficacy of inhaled exosome aerosol therapy for patients with fibrotic lung lesions. I have given them sufficient time to read the informed consent form, discuss it with others, and answered any questions they had about the study. I have provided the subject with contact information in case they encounter any issues. I have also informed the subject (or their legal representative) that they may withdraw from the study at any time without providing a reason.

Signature of the researcher: Date:

9273011

病历号：008

外泌体雾化吸入治疗肺纤维性病变的安全性和有效性的随机、单盲、安慰剂对照I期临床研究

知情同意书

版本号/版本日期：V3.0/20230408

研 究 单 位 ：海南医学院第一附属医院

合作研究单位：清华大学

上海金卫细胞组织储备服务有限公司

知情同意书

第一部分 受试者须知

我们将要开展一项外泌体雾化吸入治疗肺纤维性病变的安全性和有效性的随机、单盲、安慰剂对照Ⅰ期临床研究。因为您的情况可能符合该项研究的入组条件，因此，我们想邀请您参加该项研究。本知情同意书将向您介绍该研究的目的、步骤、获益、风险、费用等，请您仔细阅读后慎重做出是否参加研究的决定。当研究人员向您说明和讨论知情同意书时，您可以随时提问并让研究人员向您解释其中您不明白的地方。您可以与家人、朋友以及您的医生讨论之后再做决定。

若您目前正参加其他临床研究，请告知研究人员。

本项研究的项目负责人是海南医学院第一附属医院的黄华萍教授、郝新宝教授，本项研究的合作研究单位为清华大学和上海金卫细胞组织储备服务有限公司。

1.为什么进行这项研究？

感染以及多种因素所致肺纤维性病变目前临床上尚无有效的治疗手段。因此，解决肺纤维性病变肺部损伤修复问题，提高患者生命质量，延长预期寿命，是目前我们临床医生亟待解决的问题。

目前，我们需要先进行一项小样本、随机对照临床研究，以评估外泌体雾化吸入治疗肺纤维性病变患者的安全性和有效性。

2.哪些人将被邀请参加这项研究？

如果您决定参加本研究，在入选前，您需要完成事先规定的筛选评估，以确认您是否符合标准参加本次临床研究。筛选内容包括您的病史、用药史、过敏史等有关问诊信息，以及体格检查，血液检验，心电图，胸部高分辨CT检查等。如果您筛查合格，且无参加本研究的禁忌，将可以纳入本研究，观察期为4周/12周，增加入组后24周及48周的电话随访。本研究采用的是一种新的治疗方法，该疗法对孕妇及胎儿的影响不明确，故要求研究期间如有性生活必须避孕。

入选标准：

患者只有在满足以下所有标准的情况下才有资格入选本研究：

1） 年龄18岁～75岁（含临界值），性别不限。

2） 符合HRCT肺纤维性病变：

a、表现为肺部条索状、僵直高密度影或结节状高密度影。

b、表现为双肺弥漫分布的网状、线状、蜂窝状影或网状结节影。

3） 过去12周内HRCT有肺纤维性病变的典型影像表现。包括特发性肺纤维化、COPD合并纤维化、新冠感染后机化性肺炎引起的慢性咳嗽患者。

4） 能够理解并配合完成肺功能检查操作。

5） 被充分告知试验目的、方法及可能出现的不舒适，同意参加试验，并自愿签署知情同意书。

6） 依从性好，愿意按照方案的要求用药并按时接受随访检查。

排除标准：

如果患者满足以下任何一项标准，不得参加本研究：

1）既往已经接受过干细胞及外泌体的治疗。

2）不能耐受雾化吸入治疗者。

3）过敏体质或有可能危及生命的药物过敏史。

4）妊娠或近期计划妊娠、哺乳期妇女。
5）具有生育能力的男性受试者和育龄期女受试者不愿意签署治疗期间直至随访结束共 12 个月内采取有效的避孕措施。
6）筛选期前 5 年内有恶性肿瘤病史者或接受过全身抗癌治疗者。
7）存在活动性乙型肝炎或丙型肝炎病毒感染，或有 HIV 感染。
8）有器官移植病史或正等待器官移植的患者。
9）入组前 8 周内接受过手术（诊断性外科手术除外）或研究期间计划行手术者，或者入组前手术伤口尚未完全愈合者。
10） 1 个月内在服用或计划服用尼达尼布或吡非尼酮药物治疗者。
11）患有以下任何一种肺部疾病：支气管哮喘、活动性肺结核、肺栓塞、气胸、特发性肺动脉高压、闭塞性细支气管炎或其他活动性肺脏疾病。
12）目前或近 4 周内患有肺炎。
13）既往行肺切除术。
14）目前需使用氧疗且氧疗时间>15h/d 者。
15）有精神病史者、有癫痫病病史或其他中枢神经系统疾病病史者。
16）患有严重的其他系统疾病，如心肌梗死，不稳定性心绞痛，心功能不全，肝硬化，急性肾小球肾炎等。
17）筛选前 3 个月内已经参与任何其他临床试验的受试者。
18）正在参加其他临床试验。
19）依从性差，难以完成研究者。
20）研究者认为可能增加受试者危险性或干扰试验结果的任何情况。

3.多少人将参与这项研究？

本研究计划招募 24 名受试者，进行单中心、随机、单盲、安慰剂对照的临床研究，以评估外泌体吸入治疗肺纤维性病变患者的安全性和有效性。

4.该研究是怎样进行的？

共需入组受试者 24 例，通过向受试者雾化吸入外泌体进行治疗，其中安慰剂是一种外形貌似研究药物的，但不具药效的物质。给药方法：雾化吸入；给药剂量和频次：2ml/次(含：2×10^9颗粒)，每日 2 次；给药疗程：7 天。本试验为随机对照研究，试验组与对照组以 1:1 的比例进行入组。在研究结束之前，您是不知道您的分组情况。本研究包含三个时期，即筛选期、治疗期和随访期。具体流程详见临床研究流程图（表 1）。

5.参加该研究对受试者日常生活的影响？

- 当您决定是否参加本研究时，请仔细考虑如上所列的检查和随访对您的日常工作、家庭生活等可能的影响。考虑每次回访的时间与交通问题。若您对试验涉及的检查和步骤有任何疑问，可以向我们咨询。
- 在服用任何新的药物前请咨询您的研究人员。
- 考虑到您的安全以及为确保研究结果的有效性，在研究期间您不能再参加其他任何有关药物和医疗器械的临床研究。

6.参加本研究受试者的风险和不良反应？

研究人员将会监控外泌体雾化吸入的副作用。试验期间，若您发生任何副作用或不适，您应立即向研究人员报告，这是至关重要的。研究人员可能会给您其他的药物来消除副作用或不适。如果您或您的研究人员认为您无法耐受这些副作用，研究药物可能会完全停用，您可能会退出本研究。

<u>外泌体治疗的可能风险：</u>

我们将使用由上海金卫细胞组织储备服务有限公司生产的外泌体（已获得药物非临床研究质量管理规范通过证书）进行研究。外泌体雾化液的制备过程所采用的设备、试剂及技术手段均符合国家制剂制备的标准，且经严格质量检测，合格后方给患者使用。在治疗过程中受试者可能出现以下不良反应：①少数患者可能出现潮红、皮疹、低热、寒战、恶心、呕吐等轻微症状，经对症处理可缓解；②极少数患者可能出现呼吸困难、低血压或心动过速、严重过敏反应等严重症状，将立即采取积极治疗措施直至缓解；③受试者研究期间有妊娠可能，目前外泌体治疗对孕妇及胎儿影响不明确，如意外妊娠，存在流产、胎儿畸形风险。

我们将密切观察您有可能发生的不良事件/不良反应。研究期间您一旦出现任何不适，或病情发生新的变化，或任何意外情况，不管是否与治疗有关，请及时向您的主管医生报告，医生将对此做出判断并给予恰当的医疗处理。

本次临床研究也可能出现治疗无效的情况，以及因治疗无效或者因合并其他疾病等原因而导致病情继续发展。在研究期间，如果医生发现本项研究所采取的治疗措施无效，将会立即中止研究，改用其他可能有效的治疗措施。

射线的风险：

您在参加本临床试验过程中，由于临床检查而需要接受射线照射检查。由于射线对人体能产生生物学效应，医生会在满足需要的前提下，最大程度的减少您接受射线的剂量。辐射剂量均在国家和国际规定的允许范围内。

心电图（ECG）：

本试验过程中，您需要接受12导联心电图的检查，可能的风险为电极片造成的皮肤刺激或除去电极片时造成的疼痛。

血压：

血压计的袖套也可能引起您的不适或上臂擦伤。

抽血的风险

从胳膊抽血的风险包括短暂的不适和/或青紫。尽管可能性很小，也可能出现感染、出血过多、凝血或晕厥的情况。

生殖风险

对女性受试者：如果您正在哺乳、妊娠，或认为自己可能妊娠或备孕，您不能参加本研究。为参加本研究，您必须持续避孕至研究结束。如果您有性生活，您应使用被您、研究人员都可接受的避孕方法。在参加本研究期间，如果您妊娠或认为可能妊娠，应立即告诉研究人员，这是至关重要的。如果您妊娠，您将被中止研究，研究人员会与您讨论您下一步的处理。

对男性受试者：参与本研究可能会损伤您的精子，而给您在研究期间孕育的孩子带来伤害。这种伤害目前是无法预测的。请您告知您的伴侣这种对未出生婴儿的风险。她应当了解到如果她怀孕了，您需要立刻告知您的研究人员，而她也应该立刻告知她的医生。

其他风险：

因研究过程中涉及进行 SGRQ 生活质量问卷调查，在问卷调查过程中可能引起您的心理不适，如果某些问题可能会让您感到不舒服，您可以拒绝回答。还可能存在一些目前无法预知的风险、不适、药物相互作用或不良反应。

7.参加本研究受试者可能的获益？

您参加本研究有可能获益，但我们不能保证改善您的健康状况。您也可能不会从参加本研究中得到任何直接利益。本研究所获得的信息可能具有科学价值，可能帮助开发出一种新治疗方法，因此可能帮助到具有相同需求的人群。

8. 如果不参加此研究，有没有其他备选治疗方案？

您可以选择不参加本项研究，这对您获得常规治疗不会带来任何不良影响。目前针对您的健康情况，对于HRCT肺纤维性病变患者，尚无有效的治疗方法。

9. 是否一定要参加并完成本项研究？

您是否参加这个研究完全是自愿的。如果您不愿意，可以拒绝，这对您目前或未来的卫生医疗不会有任何负面影响。即使您同意参加，您也可以在任何时间改变主意，告诉研究人员退出研究，您不会因退出试验而遭到歧视或报复，也不会影响您获得正常的医疗服务。当您决定不再参加本研究时，希望您及时告知您的研究人员，研究人员可就您的健康状况提供建议和指导。

当出现以下情形时，您可能会中止参加本项研究：

(1) 您自愿退出本项研究；
(2) 您出现III级及以上且经研究者判断为与外泌体相关的不良事件；
(3) 您出现严重不依从；
(4) 发生妊娠、严重不良事件、死亡或者失访；
(5) 研究者认为其它有必要退出研究的情况。

对于中途退出的受试者，出于安全性考虑，研究人员会尽最大努力继续完成对您的随访观察，您有权拒绝。您在退出研究后，也应进入随访期，您将接受周期性随访来了解生存状态。

临床研究者或者监管机构也可能在研究期间终止本研究。如果发生本研究提前终止的情况，我们将及时通知您，您的研究人员会根据您的健康状况为您下一步的治疗计划提供建议。

原则上，在您退出之后，研究人员将严密保存您的相关信息直至最终销毁，期间不会继续使用或透露这些信息。但在以下极少数情况下，研究人员将继续使用或透露您的相关信息，即使您已经退出研究或研究已经结束。这些情况包括：除去您的信息将影响研究结果的科学性或对数据安全的评价；为研究、教学或其他活动提供一些有限的信息（这些信息不会包括您的姓名、身份证号码、或者其他能识别您身份的个人信息）；当政府监管部门需要监督研究时，他们会要求查看所有的研究信息，其中也会包括您当时参与研究的相关信息。

10. 参加该项研究的费用

您将免费使用研究药物，并且研究方案规定的任何检查均是免费的，您不必为研究药物或任何研究相关的程序支付费用，参加本研究也不会向您收取其它费用。另外，在研究期间，我们将为您提供访视的交通费用补偿￥600元，具体标准如下：

- 完成筛选访视补偿￥200元。
- 完成基线访视（随机分配）补偿￥200元。
- 完成治疗结束访视补偿￥200元。

11. 发生研究相关伤害的处理？

当您的健康状况在参加本研究期间受到伤害时，请告知研究人员（联系人：及联系电话：[illegible] 13138899757）根据我国相关法规条例规定，发生与研究相关的伤害时，我们会立即采取积极的医疗措施进行救治，并依照国家现行法律给予赔偿（诊疗费及相关费用由资助方承担）。倘若发生与本研究相关的超出预期的造成后果的严重不良事件，且符合已投保的临床研究责任险条款（临床试验责任险由资助方购买），将由保险公司进行赔付。

12. 若参加研究，我需要做什么？

- 提供准确的既往病史和当前病情信息。
- 告诉研究人员您在研究期间出现的任何健康问题。
- 告诉研究人员您在研究期间服用的任何新药、药物、维生素或草药。
- 除非经过研究人员许可，否则不应服用任何药物或治疗，包括处方药和在药店柜台购买的药品（包括维生素和草药）。
- 按医嘱使用研究药物，按要求访视。
- 不要参加其它医学研究。
- 采取适当的避孕措施（整个研究期间）。
- 遵循研究人员的指导。
- 有任何不清楚的地方您可以随时询问。

13. 受试者的个人信息会得以保密吗？

如果您决定参加本项研究，您参加研究及在研究中的个人资料均属保密。您的血/尿标本将以研究编号数字而非您的姓名加以标识。可以识别您身份的信息将不会透露给研究小组以外的成员，除非获得您的许可。所有的研究成员都被要求对您的身份保密。您的档案将保存在有锁的档案柜中，仅供研究人员查阅。为确保研究按照规定进行，必要时，政府管理部门或伦理委员会的成员按规定可以在研究单位查阅您的个人资料。这项研究结果发表时，将不会披露您个人的任何资料。

14. 与研究相关的新信息？

在试验过程中我们可能会获知有关治疗的新的信息，我们会及时通知您，让您决定是否继续参加研究或退出。

15. 如果有问题或困难，该与谁联系？

如果您有与本研究相关的任何问题，请联系黄[illegible]医生，联系电话：13138117957。

如果您有与受试者自身权益相关的问题，可与海南医学院第一附属医院医学研究伦理委员会联系，联系电话：0898-66735891。

临床研究流程图

访视	筛选期	随机	治疗观察期							治疗后评估期			电话随访期	
时间窗	24h-48h	D_0	D_1	D_2	D_3	D_4	D_5	D_6	D_7	D_8	D_{4w}	D_{12w}	D_{6m}	D_{12m}
访视次数	访视1	访视2	访视3	访视4	访视5	访视6	访视7	访视8	访视9	访视10	访视11	访视12	访视13	访视14
知情同意书	√													
入选/排除标准	√	√												
身高/体重	√										√	√		
体温	√	√	√	√	√	√	√	√	√	√	√	√		
呼吸次数	√	√	√	√	√	√	√	√	√	√	√	√		
体格检查	√	√	√	√	√	√	√	√	√	√	√	√		
血常规		√								√		√		
尿常规+妊娠		√								√		√		

试验														
传染病筛查		√										√		
血生化[a]		√								√		√		
肿瘤标记物		√										√		
IL-6、IL-10		√								√		√		
外周血免疫细胞功能		√								√		√		
外周血淋巴细胞亚群		√								√		√		
胸部 HRCT		√									√	√		
心电图		√								√		√		
指端血氧饱和度		√				√				√	√	√		
肺功能		√				√				√	√	√		
6 分钟步行距离		√				√				√	√	√		
圣乔治呼吸问卷（SGRQ）		√				√				√	√	√		
莱斯特咳嗽问卷		√				√				√	√	√		
mMRC 呼吸困难量表		√				√				√	√	√		
急性加重事件			√	√	√	√	√	√	√	√	√	√		
病情进展事件[c]			√	√	√	√	√	√	√	√	√	√		
疾病结局			√	√	√	√	√	√	√	√	√	√		
AE/SAE			√	√	√	√	√	√	√	√	√	√	√	√

a)包括肝肾功能、血糖、电解质、血脂

研究人员告知声明

我已告知该受试者（和其法定代理人）<u>吸入外泌体雾化液治疗肺纤维性病变患者的安全性及有效性的临床研究</u>的研究背景、目的、步骤、风险及获益情况，给予他/她足够的时间阅读知情同意书、与他人讨论，并解答了其中有关研究的问题；我已告知该受试者当遇到问题时的联系方式；我已告知该受试者（或法定代理人）他/她可以在研究期间的任何时候无需任何理由退出本研究。

研究人员签名：[illegible]　　　　日期：2025-12-04-15:30

知情同意书·同意签字页

受试者知情同意声明

我已被告知外泌体雾化吸入治疗肺纤维性病变的安全性和有效性的随机、单盲、安慰剂对照I期临床研究的背景、目的、步骤、风险及获益情况。我有足够的时间和机会进行提问，问题的答复我很满意。我也被告知，当我有问题，或想进一步获得信息，应当与谁联系。我已经阅读这份知情同意书，并且同意参加本研究。我知道在研究期间任何时刻无需任何理由我都可以退出本研究。我同样清楚，如果我中途退出研究，特别是由于药物的原因使我退出研究时，我若将病情变化告诉医生，完成相应的体格检查和理化检查，这将对我本人和整个研究十分有利。我同意药品监督管理部门、伦理委员会或申办者代表查阅我的研究资料。

最后，我决定同意参加本项研究，并遵从医嘱。我被告知我将得到这份知情同意书的副本，上面包含我和研究人员的签名。

受试者签名：[illegible]　　日期：2023.12.4. 15:29分

法定代理人签名【如适用】：　　日期：

与受试者关系：
