# Supplementary material for: Clinical investigation on nebulized human umbilical cord MSC-derived extracellular vesicles for pulmonary fibrosis treatment
Source: Signal Transduct Target Ther. 2025 Jun 4;10:179. doi: 10.1038/s41392-025-02262-3 (PMC12134356; doi:10.1038/s41392-025-02262-3)
Supplement: Supplementary file 27 — Consent Form for Perinatal Tissue Donation [file 41392_2025_2262_MOESM27_ESM.pdf]

Informed Consent for Perinatal Tissue Donation

Dear Mother,

We invite you to join the Perinatal Tissue Donation Program (☑ Umbilical Cord ☑ Amniotic Membrane ☑ Placenta). Through this program, we will extract bioactive substances and regenerative stem cells from perinatal tissues for scientific research and clinical treatment.

In recent years, scientific research has discovered that perinatal tissues contain various bioactive substances and regenerative stem cells that may provide more effective treatments for conditions that are difficult to cure, such as neurodegenerative diseases, stroke, spinal cord injuries, autoimmune diseases, and organ failure. These advances could improve patients' quality of life, offering hope for recovery and a new beginning to thousands of patients.

Under normal circumstances, perinatal tissues are treated as medical waste after childbirth.

[Donation Process]

For research purposes, we will collect health information from both mother and baby, such as name, age, relevant test results, and the baby’s gender. Additionally, we will conduct infectious disease testing on the umbilical cord blood. We will not charge you for any of the tests performed on perinatal tissues. Only qualified and well-preserved perinatal tissues will be used to extract bioactive substances and prepare stem cells. Any tissues that do not meet the required standards will be disposed of as medical waste.

[Confidentiality]

All collected health information from mother and baby will be archived and will only be accessible to physicians and approved personnel. Under no circumstances will this information be disclosed.

[Risks]

Perinatal tissues will be collected only after you have fully understood and signed this informed consent form. The collection will be carried out by a physician after the

complete separation of the mother, baby, placenta, and umbilical cord, and will not cause any harm or impact to either mother or baby. The tissues will then be scientifically processed and stored by professional technicians.

[Benefits]

You will not directly benefit from this donation. However, your donated perinatal tissues will be used in scientific research and clinical exploration, leading to the development of new treatment strategies that will ultimately benefit the public.

[Rights]

You have the right to consult with the physician regarding any questions about the perinatal tissue donation. You may withdraw your consent before the tissues are collected and used, without affecting or harming any of your medical treatments or procedures.

We sincerely thank you for supporting the development of national biotechnology.

Signature Page for Informed Consent

Donor informed declaration	I, along with my spouse, have read the above informed consent form and had the opportunity to ask questions.<br>I voluntarily agree to the donation.<br>Donor's Signature (Block Letters):<br>Contact number: Date:
Stem cell bank statement	I hereby certify that I have explained the purpose, benefits, risks, and other relevant matters concerning perinatal tissue donation to the individual mentioned above and have thoroughly answered any related questions.<br>Stem Cell Bank Representative's Signature (Block Letters):<br>Contact number: Date:

围产期组织捐献知情同意书

亲爱的产妇，您好，现邀请您加入围产期组织捐献计划（☑脐带 ☑羊膜 ☑胎盘），我们将从围产期组织中提取生物活性物质以及具有再生功能的干细胞，用于科学研究和临床治疗。

近年来，科学研究发现围产期组织中含有多种生物活性物质和具有再生功能的干细胞，有可能对一些临床难以治愈的疾病如神经退行性病变、脑卒中、脊髓损伤、自发性免疫性疾病和脏器功能衰竭等提供更有效的治疗方式，从而提高患者的生活质量，为成千上万的患者带来康复的福音和重生的希望。

正常情况下，在新生儿出生后，围产期组织将作为医疗废弃物进行处理。

【捐献流程】

因研究需要，我们会采集母婴的健康信息如姓名、年龄、相关检查结果、胎儿性别等，并对脐带血进行传染病学检查。我们对围产期组织进行的各种检测项目，不会收取您的任何费用。只有经检查合格并保存完好的围产期组织才会用于提取生物活性物质及制备干细胞，对不符合要求的围产期组织将根据要求按医疗废弃物进行处理。

【保密】

所有采集的母婴健康信息将存档，只有医师和经批准同意的相关人员才能获取，不会在任何情况下泄露该信息。

【风险】

我们会在您完全知情同意并签署本知情同意书后采集围产期组织。围产期组织的采集将由医师在母婴与胎盘、脐带完全分离后进行，不会对母婴产生任何影响及损害。此后由专业技术人员对围产期组织进行科学操作后储存。

【收益】

您不会从本次捐献中直接受益，然而，您捐献的围产期组织将用于科学研究和探索临床治疗，开发新的治疗策略，最终造福百姓。

【权利】

您有权向医师咨询围产期组织捐献的相关问题；您可以在我们获取围产期组织并进行使用前撤销知情同意书，但不会影响或损害您的任何医疗行为和过程。

衷心感谢您对国家生物科技事业发展的支持！

知情同意书签字页

捐献者知情声明	本人及本人配偶已阅读上述知情同意书，并且有机会提出问题。<br>本人自愿同意捐献。<br>捐献者签名（正楷）：李若然<br>联系电话：15201325288 2020年 1 月26日
干细胞资源库声明	兹证明我已经向上述人士解释了围产期组织捐献的目的、利益、风险等相关问题，并认真回答了有关问题的咨询。<br>干细胞资源库代表签名（正楷）：任芳丽<br>联系电话：13693203208 2020年 1 月26日
