# Supplementary material for: Clinical investigation on nebulized human umbilical cord MSC-derived extracellular vesicles for pulmonary fibrosis treatment
Source: Signal Transduct Target Ther. 2025 Jun 4;10:179. doi: 10.1038/s41392-025-02262-3 (PMC12134356; doi:10.1038/s41392-025-02262-3)
Supplement: Supplementary file 28 — Ethics Approval for EVs [file 41392_2025_2262_MOESM28_ESM.pdf]

海南医学院第一附属医院医学伦理委员会审查意见（科研）

意见号	2022(科研）第(131)号				
会议日期	NA		会议地点	NA	
研究分类	■观察性研究		□干预性研究		□其它
项目名称	外泌体雾化吸入治疗肺纤维性病变的安全性和有效性的随机、单盲、安慰剂对照 I 期临床研究				
申办者	海南医学院第一附属医院				
承担科室	呼吸内科		主要研究者	黄华萍、郝新宝	
审查文件	1. 复审申请表<br>2. 临床研究项目不以注册为目的的声明<br>3. 临床研究项目利益冲突声明<br>4. 临床研究项目经费资助的声明<br>5. 研究者手册（版本号：V2.0，版本日期：2022 年 04 月 08 日）<br>6. 临床研究方案（版本号：V2.0，版本日期：2022 年 04 月 08 日）<br>7. 知情同意书（版本号：V2.0，版本日期：2022 年 04 月 08 日）<br>8. 招募广告（版本号：V2.0，版本日期：2022 年 04 月 08 日）<br>9. 病例报告表（版本号：V2.0，版本日期：2022 年 04 月 08 日）<br>10. 研究人员情况及分工表<br>11. 修订说明				
审查类别	修正案审查		审查方式		快速审查
投票情况（NA）	应到委员： 人		实到委员： 人		回避： 人
	批准 人，修改后批准 人，不批准 人，暂停或者终止研究 人。				
审查意见	批准<br>主任/副主任委员签字：[signature]<br>医学伦理委员会（盖章）		日期：2022 年 04 月 21 日		

注意事项：

1、研究开始前，请主要研究者完成国家医学研究登记备案信息系统登记。

2、凡涉及人类遗传资源出口或者按照国家规定必须经有关部门专项审批的研究内容，均须在获得人类遗传办公室批准/备案后开展本研究。

3、如研究中发生严重不良事件，请在获悉后 24 小时内报告科研科，伦理委员会。

4、研究过程中，请依规提交修正案申请，年度/研究进展报告，安全性报告，重大违背方案，终止或者暂停已同意的研究报告，研究完成报告。

5、研究过程如有研究纳入了不符合纳入标准或符合排除标准的受试者，符合中止试验而未让受试者退出试验，给予错误治疗或剂量，给予方案禁止的合并用药等没有遵从方案开展研究的情况，或者可能对受试者的权益/健康以及研究的科学性造成显著影响等违背 GCP 原则的情况等，请及时向伦理委员会提交违背方案报告。

6、重新启动中止/暂停的研究项目，请在启动前提交重新启动申请。

7、本项目的年度/定期跟踪频率：12 个月，本意见有效期：1 年。

声明：本伦理委员会是独立运行的，其组成和运行遵循 GCP 和相关法律法规。

地址：海南省海口市龙华路 31 号， 联系电话：0898-66735891； E-mail：hyfylunli@163.com
