# Supplementary material for: Clinical investigation on nebulized human umbilical cord MSC-derived extracellular vesicles for pulmonary fibrosis treatment
Source: Signal Transduct Target Ther. 2025 Jun 4;10:179. doi: 10.1038/s41392-025-02262-3 (PMC12134356; doi:10.1038/s41392-025-02262-3)
Supplement: Supplementary file 29 — Phase I clinical registration [file 41392_2025_2262_MOESM29_ESM.pdf]

外泌体雾化吸入治疗肺纤维性病变的安全性和有效性的随机、单盲、 安慰剂对照 I 期临床研究

下载XML文档

注册号：<br>Registration number：	ChiCTR2300075466
最近更新日期：<br>Date of Last Refreshed on：	2024-02-07
注册时间：<br>Date of Registration：	2023-09-06
注册号状态：	补注册
Registration Status：	Retrospective registration
注册题目：	外泌体雾化吸入治疗肺纤维性病变的安全性和有效性的随机、单盲、 安慰剂对照 I 期临床研究
Public title：	A randomized, single-blind, placebo-controlled, phase I clinical study of the safety and efficacy of nebulized exosomes of human umbilical mesenchymal stem cells in the treatment of pulmonary fibrosis manifested by HRCT
注册题目简写：	
English Acronym：	
研究课题的正式科学名称：	外泌体雾化吸入治疗肺纤维性病变的安全性和有效性的随机、单盲、 安慰剂对照 I 期临床研究
Scientific title：	A randomized, single-blind, placebo-controlled, phase I clinical study of the safety and efficacy of nebulized exosomes of human umbilical mesenchymal stem cells in the treatment of pulmonary fibrosis manifested by HRCT
研究课题代号(代码)：<br>Study subject ID：	
在二级注册机构或其它机构的注册号：<br>The registration number of the Partner Registry or other register：	MR-46-22-004531
