# Supplementary material for: Clinical investigation on nebulized human umbilical cord MSC-derived extracellular vesicles for pulmonary fibrosis treatment
Source: Signal Transduct Target Ther. 2025 Jun 4;10:179. doi: 10.1038/s41392-025-02262-3 (PMC12134356; doi:10.1038/s41392-025-02262-3)
Supplement: Supplementary file 30 — Ethics Committee of Seventh Medical Center of Chinese PLA General Hospital [file 41392_2025_2262_MOESM30_ESM.pdf]

中国人民解放军总医院第七医学中心医学伦理委员会
项目评审报告

项目编号： 20200016

经中国人民解放军总医院第七医学中心医学伦理委员会医学委员会专家评审后，认为下列项目医学研究伦理规范，捐赠者拥有该项目的知情权，取得知情同意书方式适当，并要求严格按照研究方案和知情同意原则开展工作。

项目名称： 脐带胎盘组织以及衍生物用于实验动物慢性炎症的科学研究

项目负责人：吴鸿雁

单　　位： 中国人民解放军总医院第七医学中心妇产科

资金来源： 横向课题

提交日期： 2020-1-11

评审日期： 2020-1-19

批准日期： 2020-1-31

中国人民解放军总医院第七医学中心医学伦理委员会

Ethics Committee of Seventh Medical Center of Chinese PLA General Hospital

Project No: 20200016

PROJECT TITLE: Umbilical cord and placental tissues and derivatives used in scientific studies of chronic inflammation in laboratory animals

PRINCIPAL INVESTIGATOR OF PROJECT: Hongyan Wu

INSTTUTE: Gynaecology and Obstetrics, Seventh Medical Center, Chinese PLA General Hospital, Beijing, China.

FUNDING AGENCY: Horizontal Topic Foundation

DATE SUBMITTED: Jan 11, 2020

DATE FOR WHICH REVIEWED: Jan 19, 2020

DATE APPROVED: Jan 31, 2020

The Ethics Committee of Seventh Medical Center of Chinese PLA General Hospital has reviewed the proposed donators in the above-mentioned project. The right and the welfare of the subject are adequately protected; the potential risks are outweighed by potential benefits.

The Ethics Committee of Seventh Medical Center of Chinese PLA General Hospital has approved the entirety of the project and finds it concurrent with all related protocols.

Ethics Committee of Seventh Medical Center of Chinese PLA General Hospital

jinjin sheng
