# Supplementary material for: Clinical investigation on nebulized human umbilical cord MSC-derived extracellular vesicles for pulmonary fibrosis treatment
Source: Signal Transduct Target Ther. 2025 Jun 4;10:179. doi: 10.1038/s41392-025-02262-3 (PMC12134356; doi:10.1038/s41392-025-02262-3)
Supplement: Supplementary file 31 — INSTITUTIONAL ANIMAL CARE AND USE COMMITTEE FORM OF TSINGHUA UNIVERSITY [file 41392_2025_2262_MOESM31_ESM.pdf]

INSTITUTIONAL ANIMAL CARE AND USE COMMITTEE FORM OF TSINGHUA UNIVERSITY

Form ID : THU-LARC-2024-008

1. Title of application: Umbilical cord-derived mesenchymal stem cells preferentially modulate macrophages to alleviate pulmonary fibrosis

2. Principal investigator: Zhijie Chang

3. Certification of institutional animal care and use committee (IACUC) or declaration of exemption:
This animal activity has been reviewed and approved by the IACUC.

The IACUC Identification Number: 20-CZJ1

Date of IACUC approved：
(if approval is pending, write "pending." Follow-up certification is required) 2020/3/20

Location of animal experiments: LARC Tsinghua

4. Each official signing below certifies that the information provided on this form is correct and that each institution assumes responsibility for assuring required future reviews, approvals, and submission of certification.

APPLICANT INSTITUTION	COOPERATING INSTITUTION
**Name, Address, and Telephone No.:** School of Medicine, Medical Science Building, Room A107, Tsinghua University, Beijing 100084, P. R. China	**Name, Address, and Telephone No.:**
**Signature of Official Listed Above (Date)** [signature] **2024/4/1**	**Signature of Official Listed Above (Date)**
**Hai Qi, Chair of IACUC**	
