# Supplementary figures and images for: Clinical investigation on nebulized human umbilical cord MSC-derived extracellular vesicles for pulmonary fibrosis treatment
Source: Signal Transduct Target Ther. 2025 Jun 4;10:179. doi: 10.1038/s41392-025-02262-3 (PMC12134356; doi:10.1038/s41392-025-02262-3)

## Slide 1
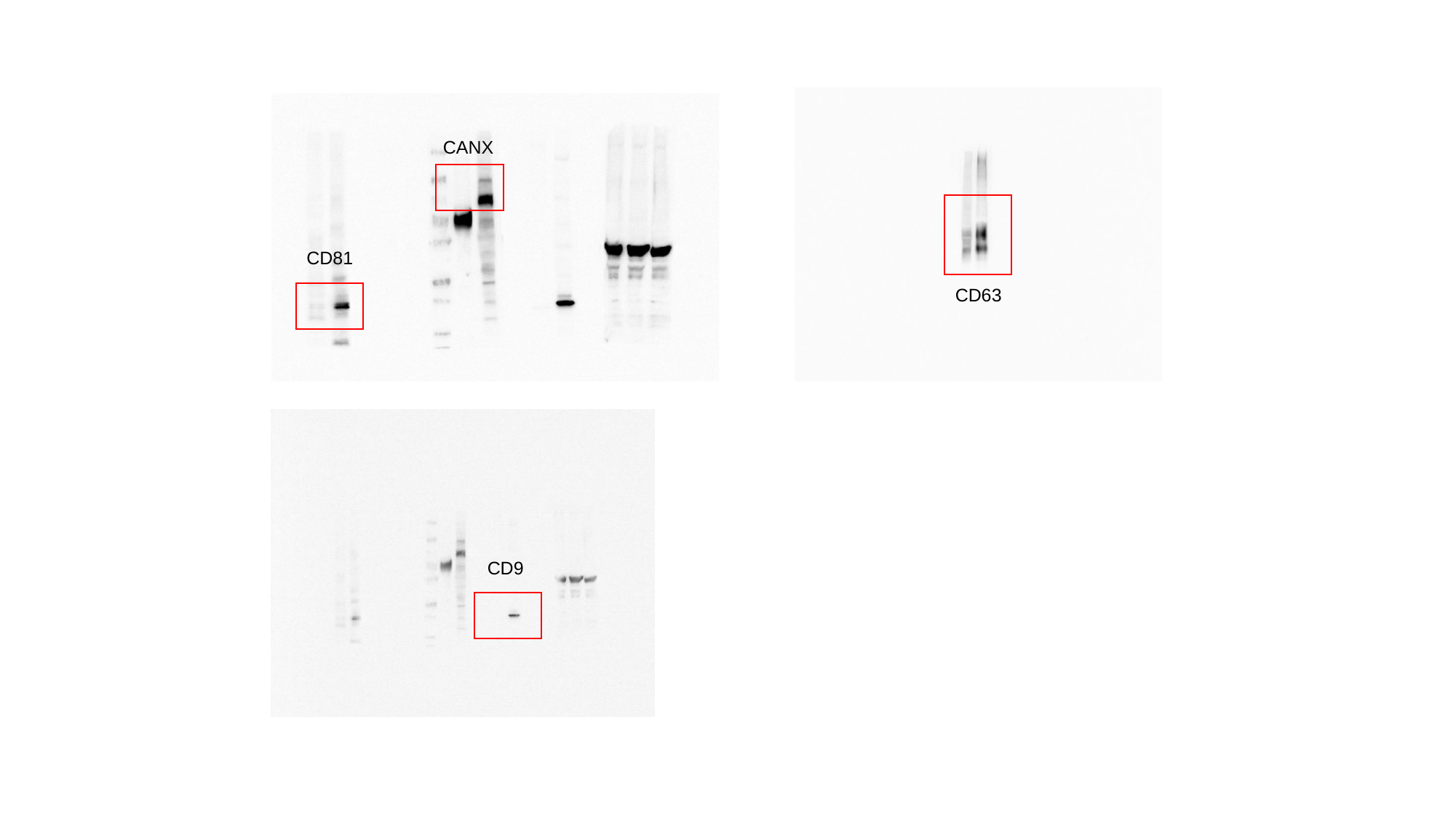

CANX
CD81
CD63
CD9

## Slide 2
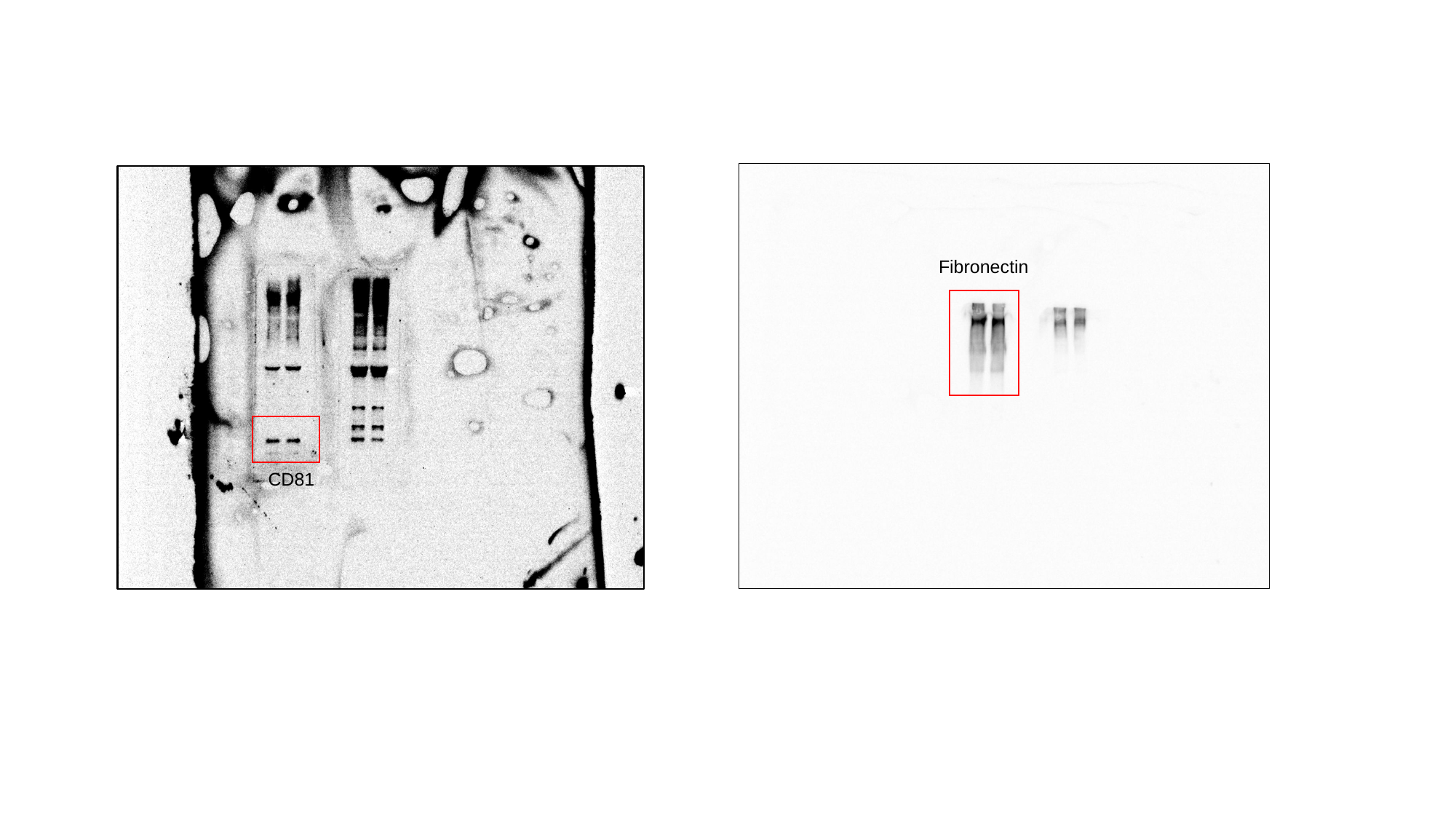

Fibronectin
CD81

## Slide 3
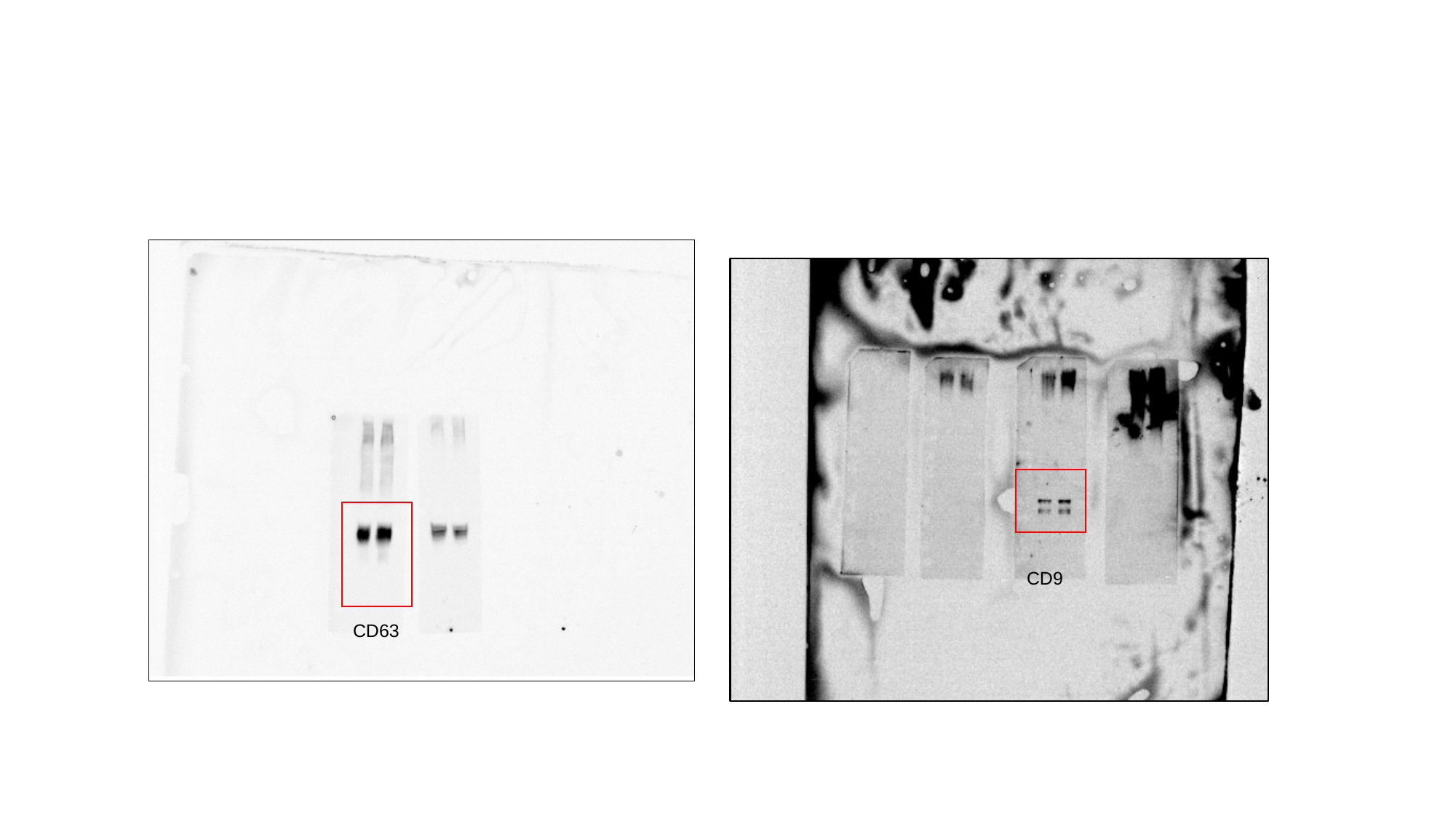

CD9
CD63

## Slide 4
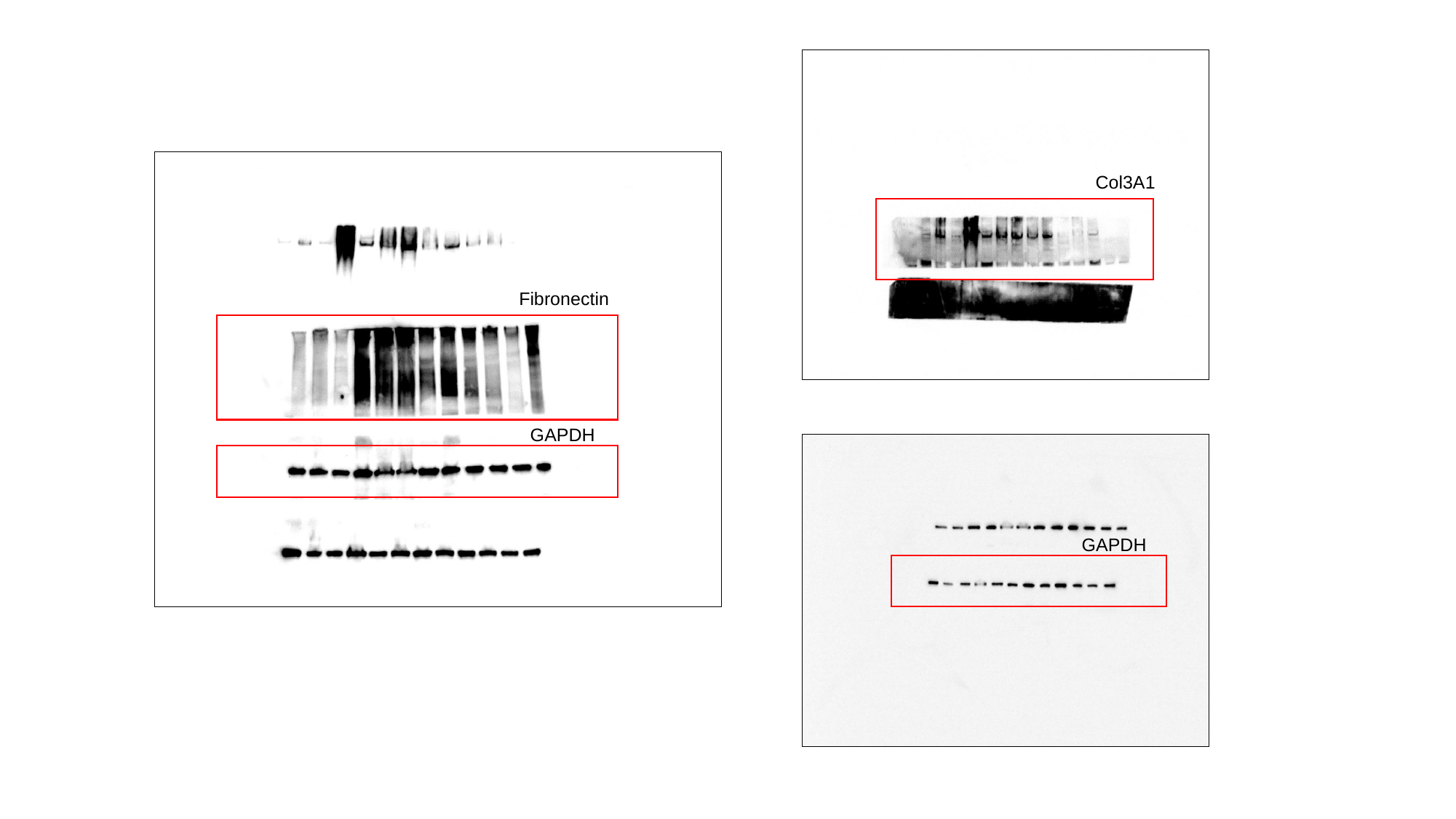

Col3A1
Fibronectin
GAPDH
GAPDH

Supplement: Supplementary file 32 — Original WB [file 41392_2025_2262_MOESM32_ESM.pptx]
